# Supplementary material for: The structural basis of N-acyl-α-amino-β-lactone formation catalyzed by a nonribosomal peptide synthetase
Source: Nat Commun. 2019 Jul 31;10:3432. doi: 10.1038/s41467-019-11383-7 (PMC6668435; doi:10.1038/s41467-019-11383-7)
Supplement: Supplementary file 1 — Supplementary Information [file 41467_2019_11383_MOESM1_ESM.pdf]

Supplementary Information

The structural basis of *N*-acyl-α-amino-β-lactone formation catalyzed by a nonribosomal peptide synthetase

D. F. Kreitler et al.

Table of Contents

Supplementary Figure 1. Operon and NRPS architecture for obafluorin biosynthesis.. S2
Supplementary Figure 2. Sequence Alignment of MLP Domains S3
Supplementary Figure 3. Size exclusion analysis of ObiF1 constructs S4
Supplementary Figure 4. Additional time points for obafluorin reconstitution................ S5
Supplementary Figure 5. Validation of thiols as trapping agents for β-lactones (I) S6
Supplementary Figure 6. Validation of thiols as trapping agents for β-lactones (II) S7
Supplementary Figure 7. Validation of reconstituted obafluorin biosynthesis S8
Supplementary Figure 8. DHB-AMP levels in the reconstitution assay......................... S9
Supplementary Figure 9. Scheme for acyl transfer and β-lactone cyclization............. S10
Supplementary Figure 10. Additional time points for substrate screen S11
Supplementary Figure 11. Benzoate-AMP levels in the substrate screen S12
Supplementary Figure 12. *B. diffusa obiF1* codon optimized sequence S13
Supplementary Figure 13. SDS PAGE analysis of purified BD-ObiF1........................ S15
Supplementary Figure 14. ObiF1 protein sequence and domain boundaries S16
Supplementary Figure 15. *B. diffusa obiF2* codon optimized sequence S17
Supplementary Figure 16. *B. diffusa obiH* codon optimized sequence....................... S18
Supplementary Figure 17. *B. diffusa obiD* codon optimized sequence....................... S19
Supplementary Table 1. Structural Alignments with BdObiF with prior structures S20
Supplementary Table 2. Data Collection and Refinement Statistics S21
Supplementary Table 3. PCR Primers for BD-ObiF1 Mutagenesis............................. S22
Supplementary References S23

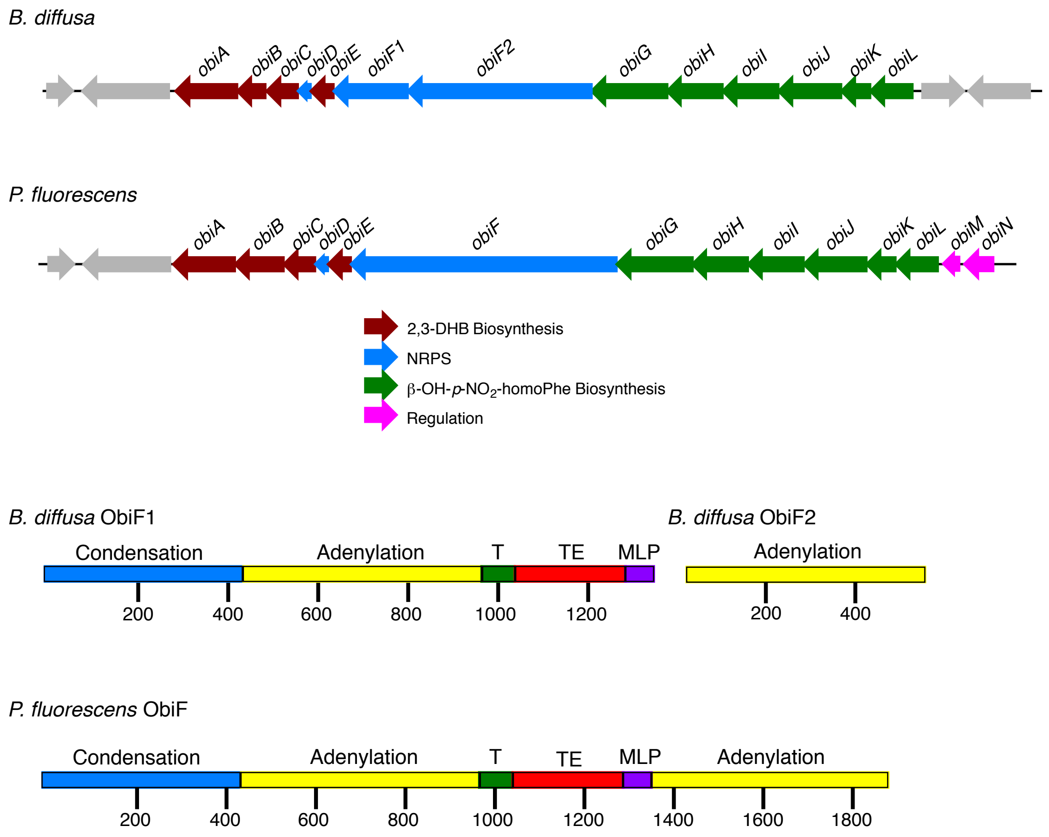

Supplementary Figure 1. Operon and NRPS systems for obafluorin biosynthesis. The gene organization of the obafluorin biosynthetic gene cluster is shown for *B. diffusa* and *P. fluorescens*. The NRPS architecture is also shown for both organisms, including the two proteins ObiF1 and ObiF2 of *B. diffusa*.

```
                          •          •
PA2412   1      MTSVFDRDDIQFQVVVNHEEQYSIWPEYKEIPQGWRAAGKSGLKKDCLAYIEEVWTDMRPLSLRQHMDKAAG*      72
MbtH     1     MSTNPFDDDNGAFFVLVNDEDQHSLWPVFADIPAGWRVVHGEASRAACLDYVEKNWTDLRPKSLRDAMVED*       71
YbdZ     1    MAFSNPFDDPQGAFYILRNAQGQFSLWPQQCVLPAGWDIVCQPQSQASCQQWLEAHWRTLTPTNFTQLQEAQ*      72
TioT     1     MSVNPFDDEDGEFYVLVNDEEQHSLWPTFGDVPDGWRIVFGPAGRAESVAYVEENWTDMRPKSLREAMSAA*       71
SlgN1    1      MSNPFEEYDGGHVVLTDALGRHSLWPAGIAVPAGWSVRHGTDSREGCLAHIEHHWTDLRPTGPAVERAPAGACVH*   75
5U89     1      MTNPFENKEGTYLVLINDEGQYSLWPASIAIPPGWNIAFAENTRSACLDYINAHWIDMRPNSLKDGSLSKRDNDY*   75
PfObiF   1313 RAFHNPFDDVEGRFSLLANDARQLSLWPEFAPTPAGWTALFGPASHSECLARTQAYDHEALISPPAPTEGLDAPYWP* 1389
BdObiF1  1317 RAFLNPFDDEDEVHYLLANDLGAHSLWPAFVPLPGGWRVVAGPASRDACLGALPNPPIGVSAAVAARETAEHCV*    1390
```

	Species	NRPS cluster	PDB
PA2412	*Pseudomonas aeruginosa*	Pyoverdine	**2PST**
MbtH	*Mycobacterium tuberculosis*	Mycobactin	**2KHR**
YbdZ	*Escherichia coli*	Enterobactin	**5JA1** (with EntF)
TioT	*Micromonospora sp. ML1*	Thiocoraline	**5WMM** (with TioS)
SlgN1	*Streptomyces lydicus*	Streptolydigin	**4GR4**
5U89	*Geobacillus sp. Y4.1MC1*	Bacillibactin	**5U89**
PfObiF	*Pseudomonas fluorescens*	Obafluorin	--
BdObiF1	*Burkholderia diffusa*	Obafluorin	**6N8E**

Supplementary Figure 2. Sequence aligment of MLP domains. Sequence alignment with COBALT[1] of MLP domains that have been studied or structurally characterized. Residues that are conserved in greater than six of the eight sequences are highlighted in red. The two tryptophan residues that form the pocket for interaction with the adenylation domain are highlighted with red circles. The stop codon is indicated with an asterisk.

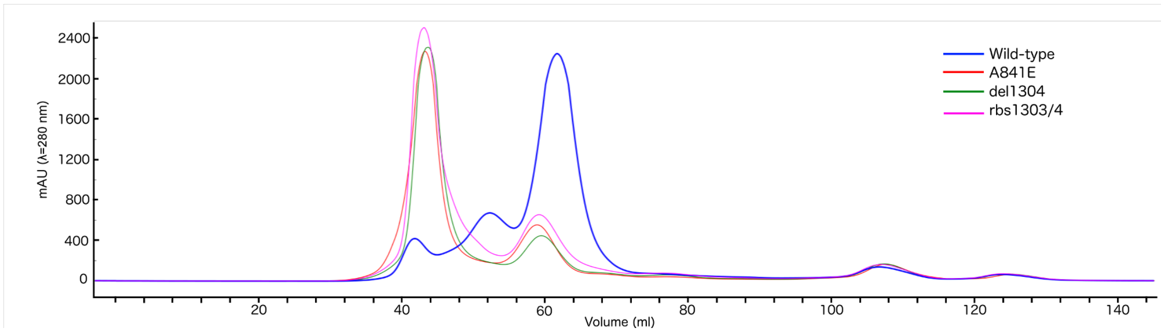

Supplementary Figure 3. Size exclusion analysis of ObiF1 constructs. Purified proteins were examined on a Hi Load 16/600 Sephadex 200 pg. Blue Dextran was analyzed to indicated the void volume and eluted at 41.6 ml. For each sample, approximately 50 mg of protein was loaded onto the column with a 5 mL sample loop following the pantetheinylation reaction. Wild-type protein (blue) eluted primarily as a monomeric sample, with a small amount of aggregated dimer and larger aggregates in the void volume. In contrast, the point mutation A841E (red), the ObiF1 delMLP (green), or the co-expressed ObiF1 + MLP samples (rbs1303/4, pink) all eluted primarily in the void volume, with less protein eluting as a monomeric sample.

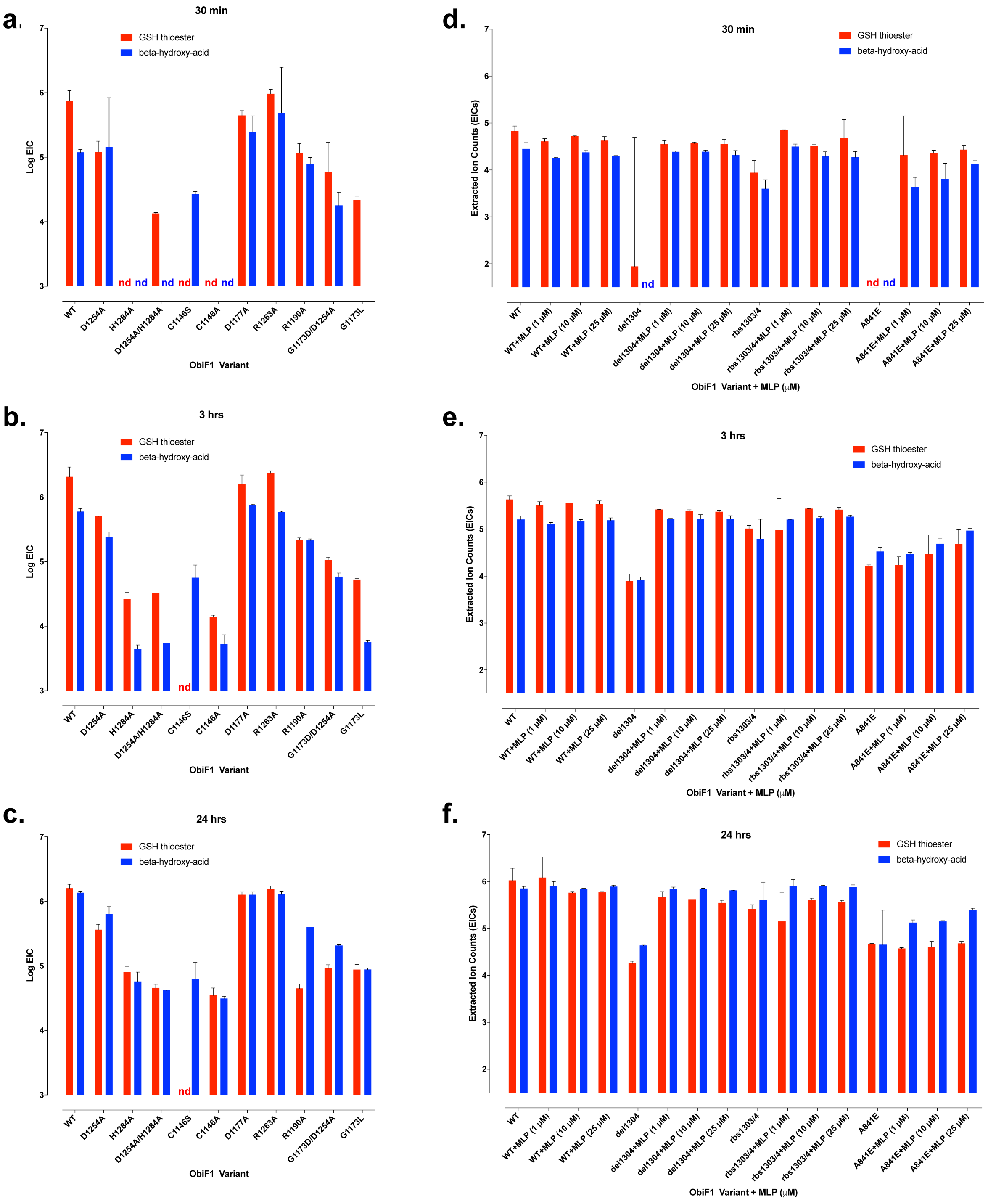

Supplementary Figure 4. Additional time points for ObiF1/F2/H/D reconstitution assay. Assays for active site variants were tested at a) 30 min, b) 3 h, and c) 24 h. Assays for MLP dependence were tested at d) 30 min, e) 3 h, and f) 24 h. GSH was included as a trapping agent to enable quantification of product ion counts by LCMS. Bar graphs represent extracted ion counts (EICs) for the GSH-thioester (*m/z* = 621 for [M+H]+ ion) and β-hydroxy acid (*m/z* = 332 for $[M+H]^+$ ion) normalized to a phenylalanine internal standard at the 0.5 hr (a,d), 3 hr (b,c), and 24 hr (c,d) reaction time points. The y-axis represents the log (EICs) and the x-axis denotes the ObiF1 variant used in the enzymatic reaction along with concentration of exogenous MLP (if added). Error bars represent standard deviations for at least two independent trials. nd = not detected by LCMS.

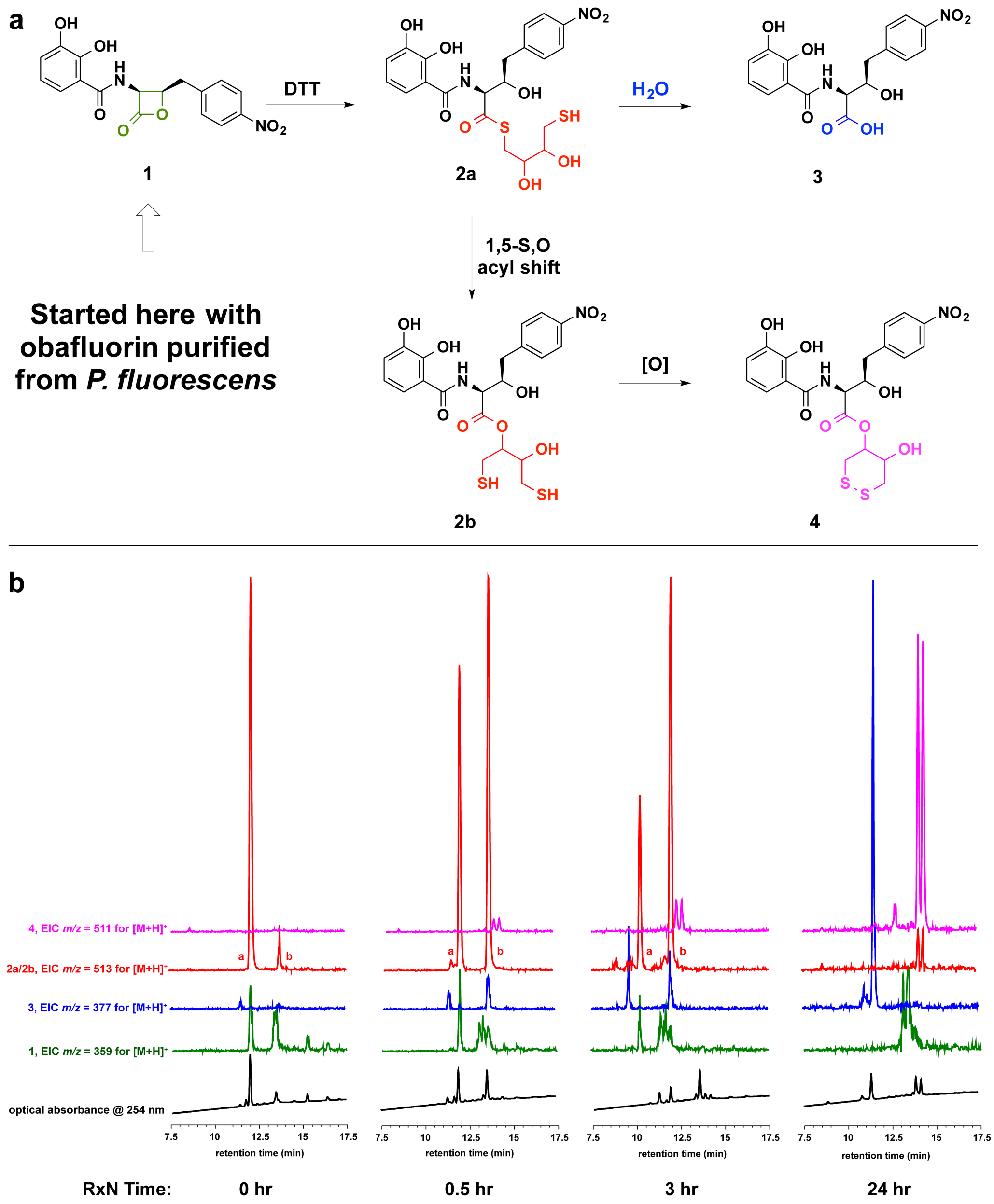

Supplementary Figure 5. Validation of Thiols as Trapping Agents for β-Lactones (I). a) Control experiment to validate use of thiols as β-lactone trapping agents. Obafluorin β-lactone (**1**) was purified from *P. fluorescens* ATCC 39502.[2] Treatment of a ~100 µM solution of obafluorin β-lactone with 500 µM DTT in 25 mM sodium phosphate buffer, pH 7.5 at room temperature leads to the formation of the DTT thioester (**2a**) that can rearrange to the stable oxoester (**2b**) via intramolecular 1,5-S,O acyl shift. Further oxidation to the cyclic disulfide (**4**) takes place in the buffer solution. Alternatively, the DTT thioester can hydrolyze to the β-hydroxy acid (**3**). b) The reaction was monitored by LCMS (instrument: Agilent 6130 quadrupole with G1313 autosampler, G1315 diode array detector, 122 series solvent module; column: Phenomenex Gemini C18, 50 x 2 mm, 5 µm plus guard column; solvents: 0.1% formic acid in (A) water and (B) acetonitrile; method: 5% B to 100% B over 20 min; software: G2710 ChemStation) and EIC traces are shown for each product along with the optical absorbance at 254 nm. Note: The “0 hr” time point was taken immediately after addition of DTT showing immediate consumption of β-lactone with quantitative formation of DTT-thioester.

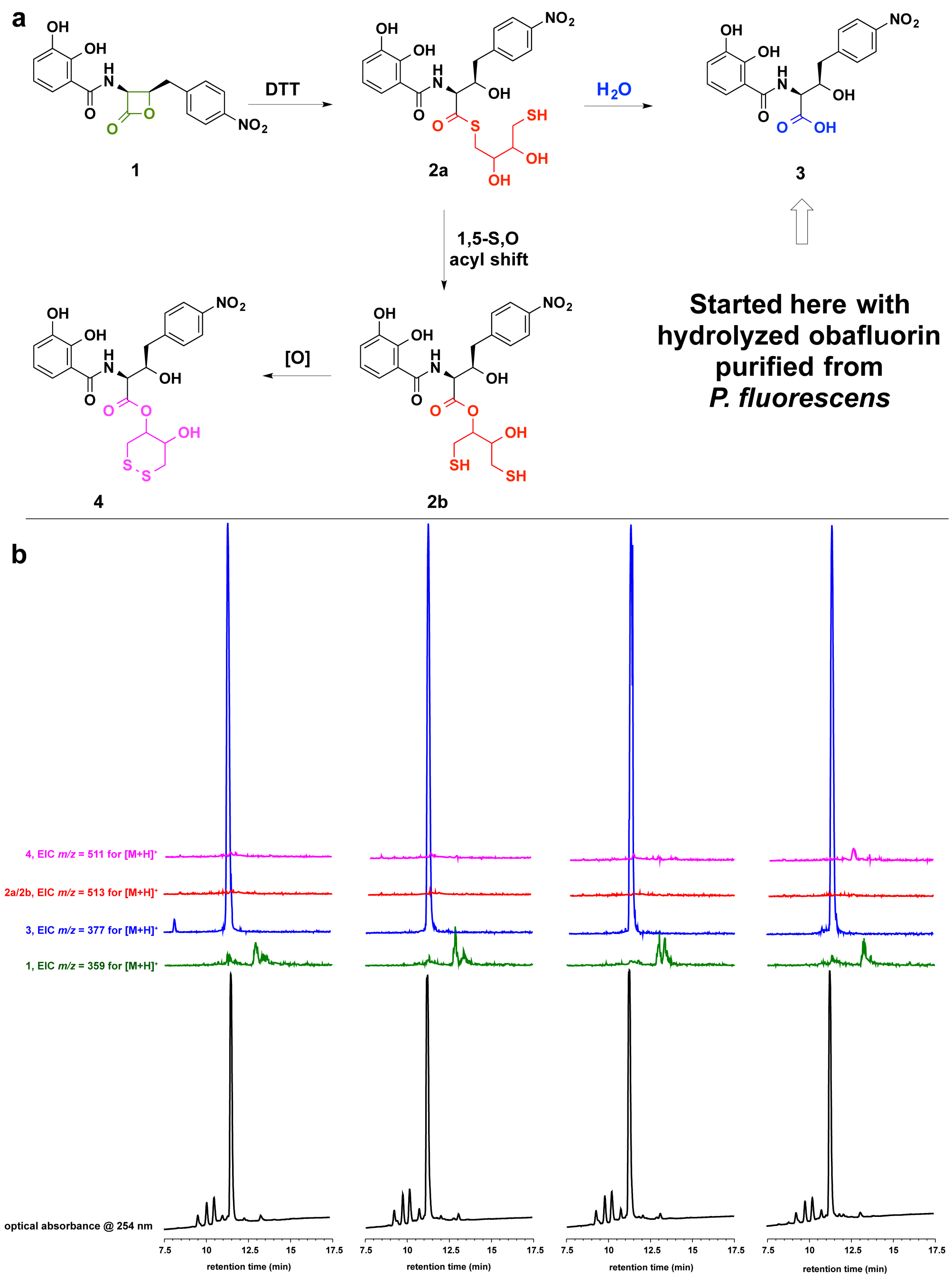

Supplementary Figure 6. Validation of thiols as trapping agents for β-lactones (II). a) Control experiment to validate use of thiols as β-lactone trapping agents. Hydrolyzed obafluorin β-hydroxy acid (**3**) was purified from *P. fluorescens* ATCC 39502.[2] Treatment of a ~100 µM solution of obafluorin β-hydroxy acid with 500 µM DTT in 25 mM sodium phosphate buffer, pH 7.5 at room temperature leads to no reaction; thus, validating that only the β-lactone NRPS product can be trapped by thiol agents. The β-hydroxy acid (**3**) is stable to the reaction conditions. b) The reaction was monitored by LCMS (instrument: Agilent 6130 quadrupole with G1313 autosampler, G1315 diode array detector, 122 series solvent module; column: Phenomenex Gemini C18, 50 x 2 mm, 5 µm plus guard column; solvents: 0.1% formic acid in (A) water and (B) acetonitrile; method: 5% B to 100% B over 20 min; software: G2710 ChemStation) and EIC traces are shown for each product along with the optical absorbance at 254 nm.

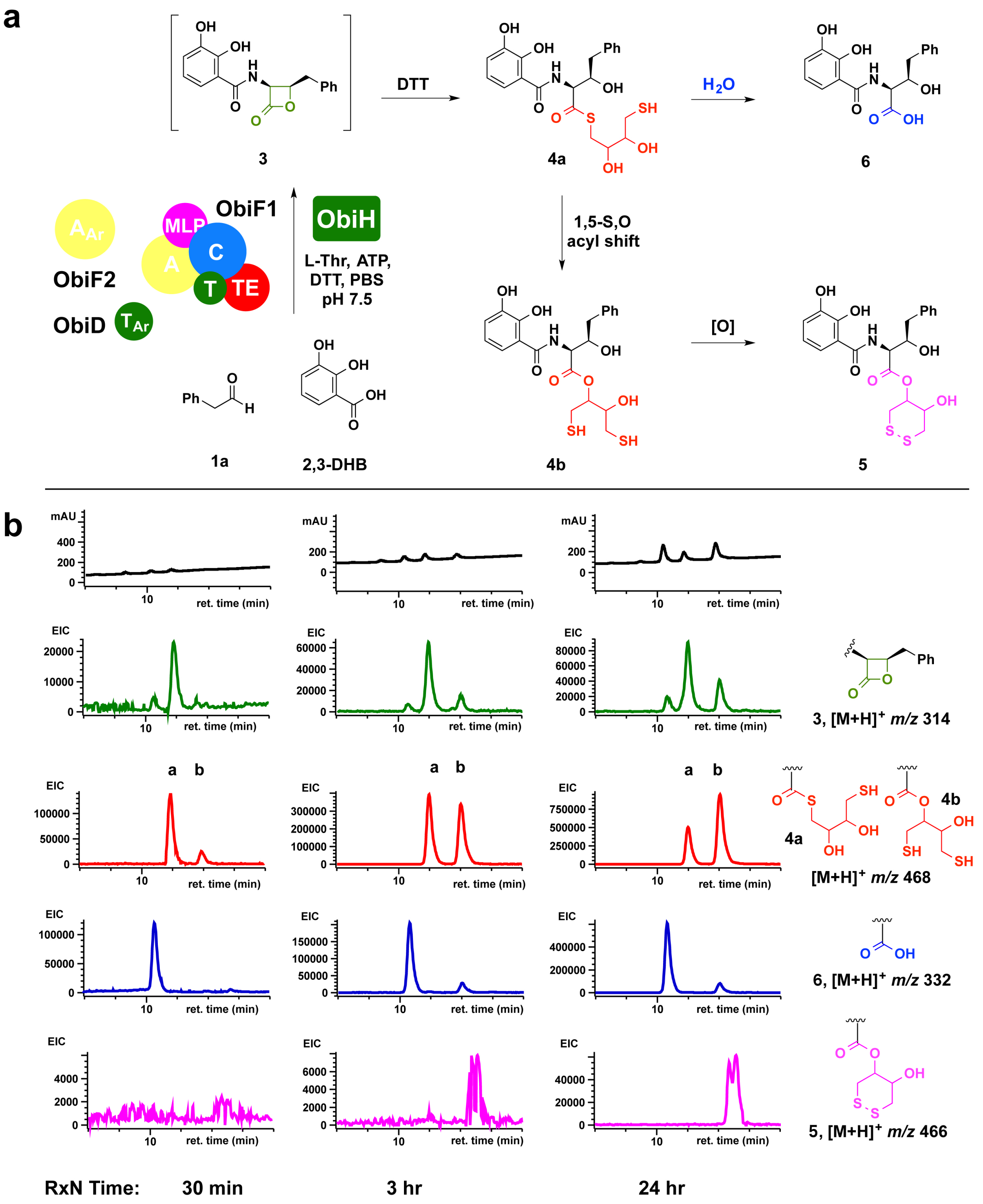

Supplementary Figure 7. Validation of reconstituted obafluorin biosynthesis. a) Enzymatic production of obafluorin analog and trapping with thiol. b) The product distribution from the in vitro ObiF1/F2/D/H reaction starting from aldehyde **1a** (PAA) and 2,3-DHB matches the product distribution for obafluorin β-lactone (Supplementary Figure 5).

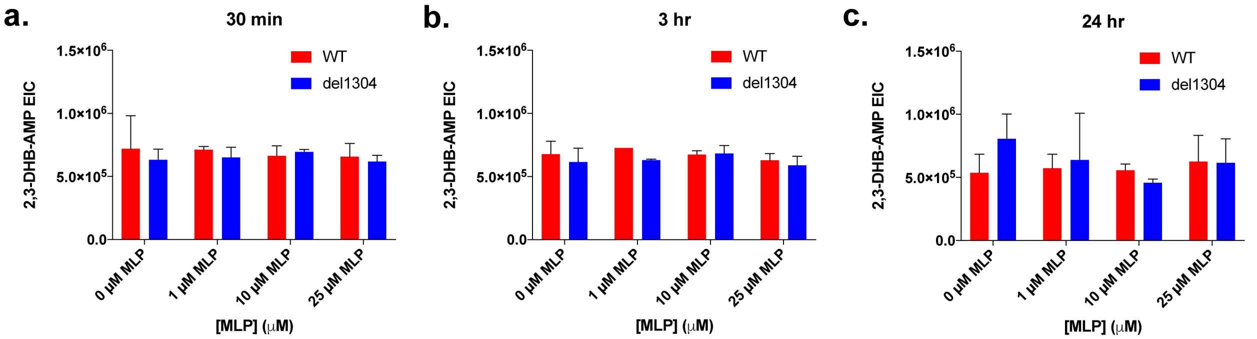

Supplementary Figure 8. DHB-AMP levels determined by LCMS in the ObiF1/F2/H/D reconstitution assay. Bar graphs represent extracted ion counts (EICs) for the 2,3-dihydroxybenzoic acid acyl adenylate (*m/z* = 506 for $[M+Na]^+$ ion) normalized to a phenylalanine internal standard at the a) 0.5 hr, b) 3 hr, and c) 24 hr reaction time points. The y-axis represents the log (EICs) and the x-axis denotes the concentration of MLP added in the enzymatic reaction. The figure legend indicates whether WT or del 1304 ObiF1 was used in the reaction. Error bars represent standard deviations for at least two independent trials. Source data are provided as a Source Data File.

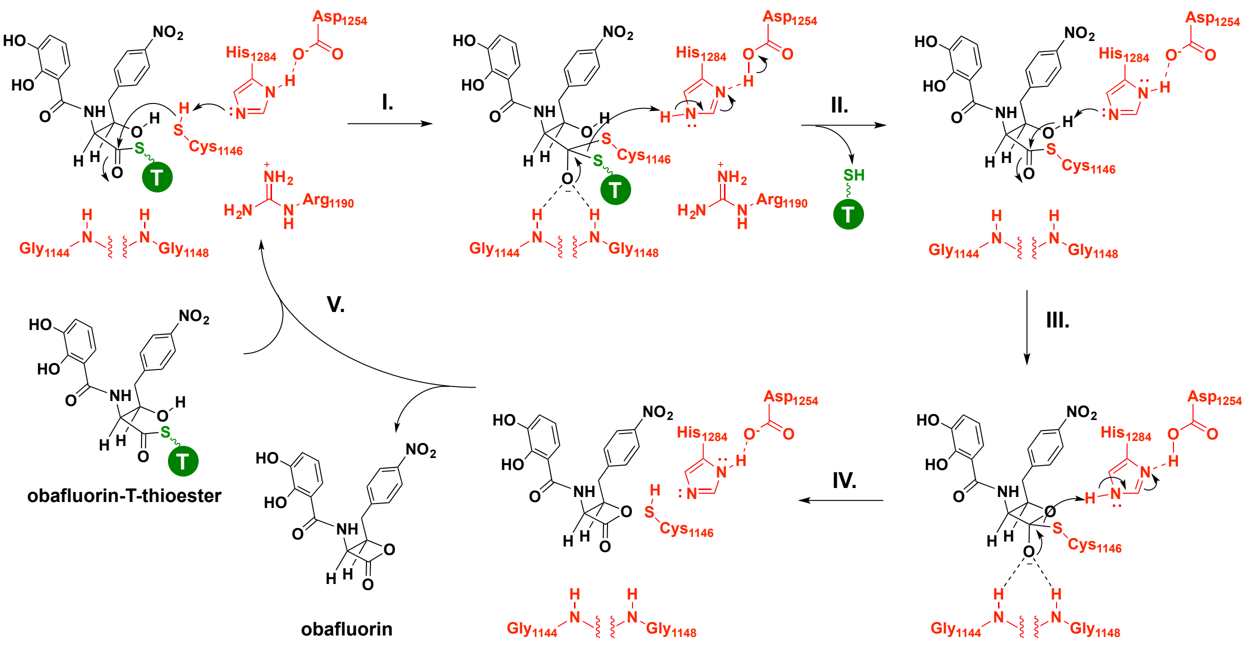

Supplementary Figure 9. Scheme for proposed acyl transfer and β-lactone cyclization during obafluorin biosynthesis. I.) The T domain thioester interacts with the TE domain to facilitate nucleophilic attack of the thioester carbonyl by the Cys1146 thiolate with assistance from the His1284/Asp1254 diad and stabilization of the tetrahedral intermediate by the oxyanion hole formed by Gly1144/Gly1148. Arg1190 is also proposed to play a role in facilitating the acyl transfer. II.) Breakdown of the tetrahedral intermediate and loss of the T domain thiolate generates the energy neutral TE domain thioester. III.) Intramolecular attack of the TE domain thioester carbonyl by the beta-hydroxy group facilitate by the His1284/Asp1254 generates a stabilized tetrahedral intermediate that can IV.) breakdown to release the TE domain Cys1146 thiolate and generate the beta-lactone product. V.) Release of the beta-lactone product and interaction with the loaded upstream T domain thioester starts the next catalytic cycle. The T domain is shown as a green sphere. TE domain residues are shown in red.

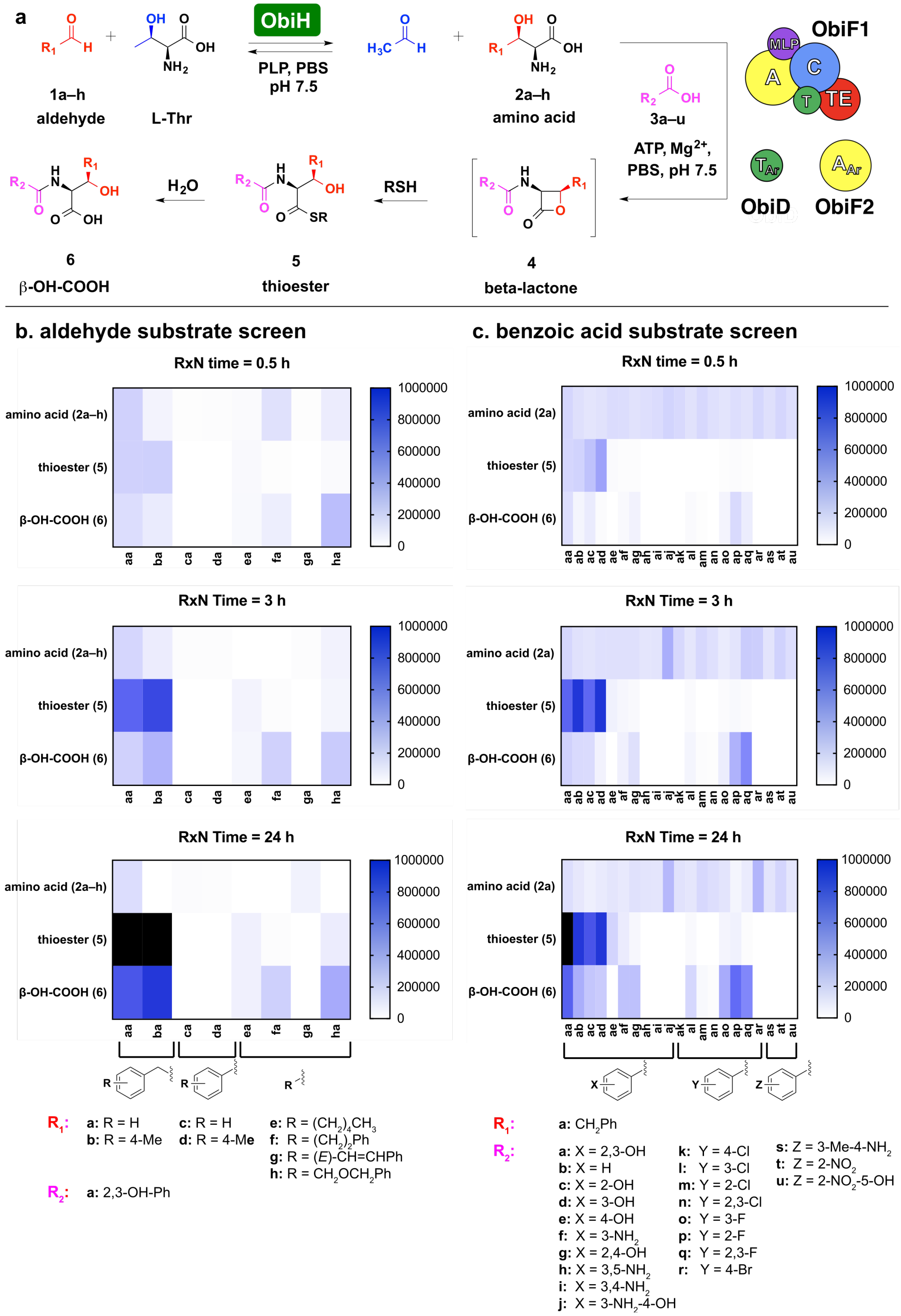

Supplementary Figure 10. Additional time points for obafluorin biosynthesis. a) Schematic reaction of substrate analogs to form lactone, thioester, and carboxylate products. Heat maps for additional time points for the ObiF1/F2/D/H b) aldehyde substrate and c) benzoic acid substrate screens shown in Figure 6 of the main text. . LCMS traces used to generate each heat map are provided in the Supplementary Note. The color scale to the right of each figure reflects peak counts; peaks above 1000000 counts are colored black.

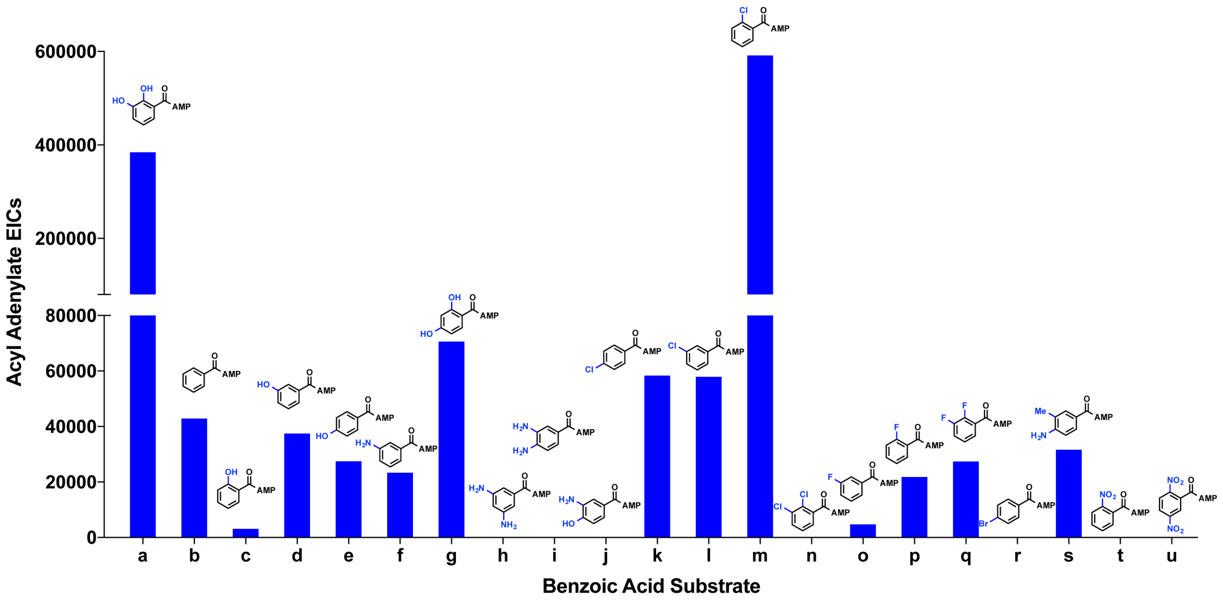

Supplementary Figure 11. Benzoate-AMP levels in the ObiF1/F2/H/D substrate screen reactions. Bar graphs represent extracted ion counts (EICs) for the benzoic acid acyl adenylate *m/z* values corresponding to either $[M+Na]^+$ ion (**a**, **g**, **k**, **l**, **m**, **q**) or $[M+H]^+$ ion (**b-f**, **h-j**, **n-p**, **r-u**) normalized to a phenylalanine internal standard (100 μM final concentration) at the 3 hr reaction time point for a single trial. The y-axis represents the log (EICs) and the x-axis denotes the identity of the benzoic acid substrate.

Supplementary Figure 12 (pg S13-S14).

```
   1 ATGGGCAGCA GCCATCATCA TCATCATCAC AGCAGCGGCC TGGTGCCGCG CGGCAGCCAT
  61 ATGCAAGCGC TGAACCCGGC GGATGTGCTG CCGCTGACCG CGGCGCAGAA CGCGATCTGG
 121 ATTGGTCATC AGCTGGATCC GGCGAGCGCG GCGTACAACG TTGCGGCGCA TGTGGGCGTT
 181 GATGCGGCGC TGGATGCGGA CCTGCTGCGT CGTGCGTTCG ACATCACCGC GAACGAAACC
 241 GATTGCCTGC GTATGCGTTT TGTGGAAACC GGTAGCGATG GTGAAGGTGC TGTTCGTCAG
 301 ACCTTTGTGG CGCGTGCGGA AACCGCGTTC GTTATGCGTG ACTTTCGTGC GGAGCCGGAT
 361 AGCACCGGTG CGGCGCATGC GTGGATGGCG GCGGACGTGC GTCGTCGTAT CGATCTGAGC
 421 AGCGGTTGCC TGGTGCATGC GGCGCTGCTG CGTACCGGTA CCCGTGACTA CGTGTATCTG
 481 CGTAGCCACC ACATTGCGCT GGATGGTTTT GGTCTGGCGA TGGTTCTGCG TCGTGTGGCG
 541 CACGTTTACG GTGCGCTGGT TGCGGGTCGT GAACCGGCGG CGGCGGCGTT CGGTGCGTTT
 601 GCGGAAGTGA TCGATGCGGA CCGTGCGTAC CATGCGAGCG CGGCGTGCGA AGCGGACCGT
 661 GCGTACTGGC GTGCGTATTG CGCGGGTCTG GATGATGTTC CGACCCTGTG CGCGGGTACC
 721 AGCCTGCCGA GCGAGATTGC GGTTTGCCAT ACCGCGCCGG TTCCGGCGGC GCTGGTTGAG
 781 CGTCTGCATG ACTTCGCGAA CGAGTGCGGT ACCCACTGGA TTAACGTGGT TGTTGCGGCG
 841 TTTGGTGCGT TTGTGGGTCG TGCGACCAGC CGTCGTGATA TCACCATTGG CGTTCCGATG
 901 ATGAACCGTC TGGGTGGCGT TGCGGCGAGC GTGCCGTGCA CCACCGCGAA CGTTCTGCCG
 961 CTGAGCCTGG ATGTGCGTCC GGGTGCGCGT GCGGAGGCGC TGGTTGAGGC GGTTGATACC
1021 GGTCTGGCGG GTATGCGTCG TCACCAGCGT TACCGTGCGG AAGACATCCG TCGTGATTGC
1081 CACCTGATTG GCGAGGGCCG TCGTCTGACC GGTCCGCAAA TCAACGTGGA CGTTTATACC
1141 GATCCGATTG CGTTTGGTGA TGCGAGCGGT ATTGCGCGTG TGGTTAGCGC GGGTCCGGCG
1201 GATGATGTGA GCCTGATGAT CCAACGTGGT GACATGGCGG ATGCGCTGAC CATTGTTGGT
1261 ATGGCGAACC CGGCGCTGTA CCGTCCGCAT GAACTGGCGC GTTGGATTGA GCGTTTCGTT
1321 GCGTTTACCA CCGCGTTTGT GGCGGATCCG AGCTGCCCGG TTGGTCGTCT GGATGCGTAT
1381 CTGCCGGGTG ATGGTGTGGA AGTTCACCTG CCGGAGCCGG CGAAGCGTAG CCTGGGTGCG
1441 ACCCTGGTTG AGGTGTTTGA ACGTCGTGTT GCGGAACGTC CGCATGCGAG CGCGGTTACC
1501 CTGGATCACA CCACCTGGGA CTACGCGGAA CTGGATGCGC GTGCGAACCG TCTGGCGCGT
1561 CACTTTGCGG CGAGCACCCC GGCGCGTGGT AACCTGCGTG TTGCGCTGCT GCTGCCGCGT
1621 ACCCTGGACG CGATCGTTGC GATTCTGGCG ACCCTGAAAT TTGGCGCGGC GTATGTGCCG
1681 ATTGATCCGG ATGCGCCGGC GGAGCGTATT CGTGCGATCA TTGACGATTG CGATGCGGCG
1741 CTGGTTGTGA CCACCGTGGA CCTGGCGAGC CGTATCGATG CGAGCGGTCG TCGTCTGGTT
1801 GTGCTGGACG CGCCGGATAC CCGTGCGGCG GTTGCGGCGG CGAGCGCGGC TCCGCCGAGC
1861 CGTGATGGTG AAGGTCCGCG TGCGGATGAT CTGGCGTACA TCATTTTCAC CAGCGGTAGC
1921 ACCGGCAAGC CGAAAGGTGT GAAGATTACC CACCGTAACG TTGTGCGTCT GTTCGAGGCG
1981 ACCGACGCGT GGTTTCACTA CCGTGACGAT GACGTTTGGA CCATGTGCCA CGCGTATGTT
2041 TTCGATGCGA GCGTGTGGGA AATGTGGGGT GCGCTGCTGC ATGGTGGCCG TCTGGTTGTG
2101 GTTCCGCCGG AGACCACCCG TGCGCCGGAT GCGCTGCTGG AACTGGTGGT TCGTGAGGGT
2161 GTGACCGTTT TTGGCCAGAT CCCGAGCGCG TTCTACCGTT TTATGGAAGC GCAAGCGGAC
2221 CACCCGGCGC TGCGTCAGGC GCTGCGTCTG CGTTATCAAT GCTTTGGTGG CGAGGCGCTG
2281 GATCCGAGCC GTCTGAAACC GTGGTTTGAC TGGCACCGTG ATAGCGGTAC CCGTCTGCTG
2341 AACATGTACG GCATCACCGA AACCACCATT AACGCGACCT ATCGTTTCAT TGACGAGCGT
2401 GATGTGGACA CCGGTCGTGG CAGCCTGATC GGTGAAGTTT ACGCGGATCT GGGCATTGTG
2461 GTTCTGGATG ATGCGCTGCG TCCGGTGCCG GCGGGTGCGT ACGGCGAGAT GTATGTTACC
2521 GGTGCGGGCC TGGCGCAGGG TTATCTGAAC CGTCCGGATC TGGACGCGGT GCGTTTTGTG
2581 GCGAACCCGT ACGGTCCGGC GGGTACCCGT ATGTATCGTA GCGGTGATGT GGCGCGTCTG
2641 CACCCGGATG GTGTTCTGGA ATACGTGGGC CGTGCGGACC AGCAAGTGAA GGTTCGTGGT
2701 TATCGTATCG AACTGGGCGA GGTTGAAGCG CGTCTGCGTG AGTATGCGCC GGTTAGCGAT
2761 GCGGTGGTTA GCGTTCGTCG TGACGCGGTG GGTGATGTTC AACTGGTGGC GCATGTGGTT
2821 GCGCGTCGTG GCGAATGCCT GGATGTGGAG GCGCTGCGTG CGCACCTGCG TGAGCGTGTT
2881 CCGGCGTATA TGGTGCCGGC GGCGTTTGGT ACCCTGGATG CGCTGCCGAT GACCCGTAAC
2941 GGCAAGGTTG ACCGTAAAGC GCTGCCGGAT ATCAGCACCG CGACCGAGCG TGTGGTTGAA
3001 CCGCCGCGTG ACGCGCTGGA TGAGCGTATT GTGGAACTGT GGCGTGAGCA GTGCGGTGAC
3061 GTTGCGATCG GCATTGATGA CAACTTCTTT GACGTGGGTG GCGATAGCAT CAAGGCGATT
3121 CGTGTGGCGC GTGCGCTGGA CATGCCGGTT ATGGCGCTGT TTGATGCGCC GACCGTTCGT
```

```
3181 GCGTGCGCGG ACTACCTGCG TGATGCGCTG GCGGATGGTG CGGGTGATGC GGCGGATCGT
3241 ACCCTGCACC ACTTCAAACG TCCGGCGCAA GCGCGTGTGC ACATGGTGTG CGTTCCGTTT
3301 GCGGGTGGCA GCGCGCTGAG CTACCGTGAA CTGGCGCGTG CGCTGCCGGA TGGTTTCGCG
3361 TGCAGCGCGC TGCAACTGCC GGGTCATGAC CCGGCGGCGC CGGATGAAGC GTTTGTGGAT
3421 CTGGACACCA CCATCGATCG TGCGGTTGAT CGTCTGCTGG CGGAGGCGGC GGCGCCGATT
3481 GTGGTTTACG GTCACTGCGC GGGTAACGCG CTGGCGGTTG CGCTGGTGCG TCGTCTGGCG
3541 GGTGCGGGTG CGAACGTTAT CGGTCTGGCG ATTGGTGGCA TGCTGCTGGA TGAAGACGCG
3601 GATGCGGTTC TGGACGAAGT GGGTGCGCGT AGCGGCGAGA ACATCGTGGA TTTCCTGCGT
3661 CAGATTGGTG GCTTTAAGGA CGTTCTGGAT GCGGGTACCC TGGCGGCGAT TGCGCGTATG
3721 ACCAAACACG ACGCGATGCA AGCGGCGACC TTCTTTGCGG CGGAGACCCG TGCGCCGGCG
3781 CGTCTGGATG TGCCGCTGCA CGTGGTTATC GGTGGCCAAG ACCCGCTGAC CCCGGATTAT
3841 GCGCGTCGTT ATCTGGACTG GCGTCGTTAC AGCGATGCGG TTGAACTGGA TGTGATTCCG
3901 GACGGTGGCC ACTATTTTGT TACCGAGCAT GCGGACACCC TGGCGGGTCT GCTGGCGGCG
3961 CGTTGGCTGC CGGCGAGCCG TGCGCCGGAG CGTGAACGTC AGGCGCTGCG TGCGTTCCTG
4021 AACCCGTTTG ATGACGAGGA CGAAGTGCAC TACCTGCTGG CGAACGATCT GGGTGCGCAC
4081 AGCCTGTGGC CGGCGTTTGT TCCGCTGCCG GGTGGCTGGC GTGTGGTTGC GGGTCCGGCG
4141 AGCCGTGATG CGTGCCTGGG TGCGCTGCCG AACCCGCCGA TCGGCGTTAG CGCGGCGGTG
4201 GCGGCGCGTG AGACCGCGGA ACACTGCGTT TGA
```

Supplementary Figure 12. *B. diffusa obiF1* codon optimized sequence. Purification tag sequence is underlined.

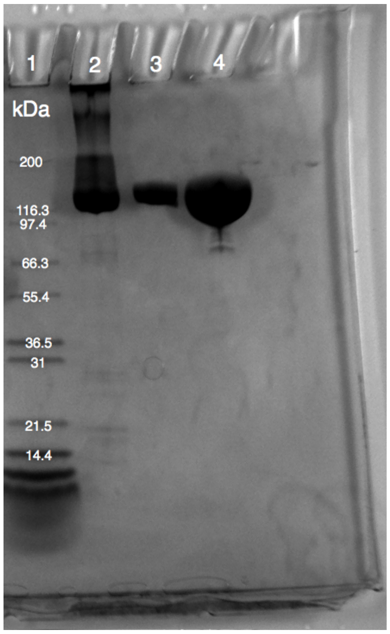

Supplementary Figure 13. SDS-PAGE analysis of purified BD-ObiF1 for crystallization. Samples taken from intermediate BD-ObiF1 protein purification steps and the final crystallization stock solution run on SDS-PAGE and stained with Coomassie blue. From left to right, lane 1) Mark12 unstained standard (ThermoFisher), 2) post-pantetheinylation reaction (12.0 µg protein), 3) Size exclusion chromatography fraction pool (1.5 µg protein), 4) crystallization stock solution (18.7 µg protein).

1 mgsshhhhhh ssglvprgsh mqaLNPADVL PLTAAQNAIW IGHQLDPASA AYNVAAHVGV

41 DAALDADLLR RAFDITANET DCLRMRFVET GSdgegAVRQ TFVARAETAF VMRDFRAEPD

101 STGAAHAWMA ADVRRRIDLS SGCLVHAALL RTGTRDYVYL RSHHIALDGF GLAMVLRRVA

161 HVYGALVAGR EPAAAAFGAF AEVIDADRAY HASAACEADR AYWRAYCAGL DDVPTLCAGT

221 SLPSEIAVCH TAPVPAALVE RLHDFANECG THWINVVVAA FGAFVGRATS RRDITIGVPM

281 MNRLGGVAAS VPCTTANVLP LSLDVRPGAR AEALVEAVDT GLAGMRRHQR YRAEDIRRDC

341 HLIGEGRRLT GPQINVDVYT DPIAFGDASG IARVVSAGPA DDVSLMIQRG DMADALTIVG

401 MANPALYRPH ELARWIERFV AFTTAFVADP SCPVGRLDAY LPGDGVEVHL PEPAKRSLGA

461 TLVEVFERRV AERPHASAVT LDHTTWDYAE LDARANRLAR HFAASTPARG NLRVALLLPR

521 TLDAIVAILA TLKFGAAYVP IDPDAPAERI RAIIDDCDAA LVVTTVDLAS RIDASGRRLV

581 VLDAPDTRAA VAAASAAPPS RDGEGPRADD LAYIIFTSgs tgKPKGVKIT HRNVVRLFEA

641 TDAWFHYRDD DVWTMCHAYV FDASVWEMWG ALLHGGRLVV VPPETTRAPD ALLELVVREG

701 VTVFGQIPSA FYRFMEAQAD HPALRQALRL RYQCFGGEAL DPSRLKPWFD WHRDSGTRLL

761 NMYGITETTI NATYRFIDER DVDTGRGSLI GEVYADLGIV VLDDALRPVP AGAYGEMYVT

*

821 GAGLAQGYLN RPDLDAVRFV ANPYGPAGTR MYRSGDVARL HPDGVLEYVG RADQQVKVRG

881 YRIELGEVEA RLREYAPVSD AVVSVRRDAV GDVQLVAHVV ARRgecldVE ALRAHLRERV

941 PAYMVPAAFG TLDALPMTRN GKVDRKALPD IStaterVVE PPRDALDERI VELWREQCGD

*

1001 VAIGIDDNFF DVGGDSIKAI RVARALDMPV MALFDAPTVR ACADYLRDAL adgagdaaDR

1061 TLHHFKRPAQ ARVHMVCVPF AGGSALSYRE LARALPDGFA CSALQLPGHD PAAPDEAFVD

1121 LDTTIDRAVD RLLAEAAAPI VVYGHCAGNA LAVALVRRLA GAGANVIGLA IGGMLLDEDA

1181 DAVLDEVGAR SGENIVDFLR QIGGFKDVLD AGTLAAIARM TKHDAMQAAT FFAAETRAPA

1241 RLDVPLHVVI GGQDPLTPDY ARRYLDWRRY SDAVELDVIP DGGHYFVTEH ADTLAGLLAA

1301 RWLpasrape RERQALRAFL NPFDDEDEVH YLLANDLGAH SLWPAFVPLP GGWRVVAGPA

1361 SRDACLGAlp nppigvsaav aaretaehcv

Supplementary Figure 14. ObiF1 protein sequence and domain boundaries. The purification tag is underlined and not included in residue numbering. Disordered residues in the crystal structures are in lower case. The domains are highlighted as in the main text: Condensation (blue), Adenylation (yellow), PCP (green), TE (red) and MLP (purple). The adenylation domain hinge (Asp873) and the pantetheinylation site (Ser1016) are highlighted with asterisks.

```
   1 ATGACCATCT TCGATAGCTT TCCGCGTTAC CCGGAGGCGG AACACTATCG TCGTGCGGGT
  61 TTCTGGACCG ATGAGACCCT GGGTGCGATG CTGCGTCGTG CGGCGGCGAC CCAGCCGGAT
 121 GCGCTGGCGA TTGTTGATGG TTGCCGTCAA GTGACCTACC GTGAACTGGA CACCATGGTG
 181 GATCGTGTTG CGAACGGTCT GACCGCGGCG GGTCTGCGTG CGGGCGATCG TGTGGTTGTG
 241 CAGCTGCCGA ACAGCATCGA GTTCGTTGAA GCGCTGTTTG GTCTGGCGCG TATTGGTGCG
 301 GTGCCGGTTA TGGCGCTGCC GAGCGACCGT ATTGCGGAGC TGAAACATGT GGCGGTTGGT
 361 ACCGGTGCGG TTGGTTACTG GGTGCAAGAC CGTGTTTTCG CGACCGATTA TCGTCTGATT
 421 GCGGCGGAGC TGCTGGATGC GGTGGAAACC GTTCGTCACG TGTTTGTTGT TGGTGATGCG
 481 GCGGGTTACC GTAGCCACGC GAGCCTGTAT GGTGTGGAGA CCCGTCGTCC GGAACCGGCG
 541 GCGAGCGCGG CGGCGCTGGT TATGCTGAGC GGTGGCAGCA CCGGCATGCC GAAATTCATC
 601 GTGCGTACCC ACGAAGATTA CCTGTATAGC GTTCGTCGTA GCGCGCAGGT GTGCGGTCTG
 661 AGCGCGTGCA GCGCGTACCT GTGCGTTCTG CCGGCGGCGC ACAACTTTAC CCTGAGCAGC
 721 CCGGGTGTTC TGGGCGTGAT CCATGCGGGT GGCTGCATTG TGATGCTGCG TGAGCCGAGC
 781 GGTCACACCG CGCTGAACGC GCTGGCGCTG CTGGACGCGG GTCGTAGCGC GTTCACCAGC
 841 CTGGTTCCGG GTCTGGCGCA GGCGTGGCTG GAAACCACCC CGCCGAACGC GTTCGTTAGC
 901 CTGGATTTTC TGCAAATCGG TGGCGCGGCG CTGAGCCCGG ATGTGGCGGC GCGTGTTGTG
 961 GAGCACTTCG GTTGCGAACT GCAGCAAGTT TTTGGTATGA CCGAGGGCAT GGTGGCGTAT
1021 ACCCGTCGTG GTGACCCGCC GGATCGTGCG CTGCATACCC AGGGTCTGCC GATGAGCCCG
1081 GCGGATGAGA TCCTGATTGT TGACGATGAC GATCGTCCGG TGCCGGATGG TGAAACCGGT
1141 CACCTGCTGG TTCGTGGTCC GTACACCATT CCGGGCTATT TTAACGCGAG CCCGCAGGCG
1201 GCGCAAGCGT TTACCCCGGA CGGTTACTAT CGTACCGGCG ATCGTGTTCG TCGTCGTGCG
1261 GACGGTTACC TGGTTGTGGA AGGCCGTCTG AAGGATCAAG TGAACCGTGG TGGCGAGAAA
1321 ATCGCGGCGG AGGAAATTGA AGCGCACCTG CTGGCGTTTC CGGGTGTGAG CGAGGCGGCG
1381 GTTGTGGGTC TGCCGGACCG TTATCTGGGC GAAGCGAGCT GCGCGTTTGT TGTGGTTGCG
1441 GACCAGATGA ACGATGAAAG CGCGTTTGCG GGTCGTCTGC GTGCGTTTGT GCGTGCGCGT
1501 GGCGTTGCGC AATTCAAGGT GCCGGACGTG GTTCTGATTG TTCCGGCGCT GCCGCGTACC
1561 ACCCTGGGTA AAACCGATAA GAAAGTGCTG CGTGCGCGTT TTGCGCCGGG TCGTGAAGAC
1621 GCGCTGGGCG CGGCGTGA
```

Supplementary Figure 15. *B. diffusa obiF2* codon optimized sequence.

```
   1 ATGAAACAAG AGCCGACCGG TGCGTTTGAA GTGGCGACCG TTCTGAACGA CATTTTTAGC
  61 GCGGATCACC GTTACCGTGA GGCGACCCTG AGCCTGACCG CGAACGAGAA CTATCCGAGC
 121 GAACTGGTTC GTGTTACCAG CGGTAGCACC GCGGGTGCGT TCTACCACGT GAGCTTCCCG
 181 TTTGACGTTC CGGATGGCGA GTGGCACTTT CCGGAACCGG GTCACATGCA TGCGGTGGCG
 241 GACAAGGTTC GTAGCCTGGG TAAAAGCCTG CTGCACGCGC AAACCTTCGA TTGGCGTCCG
 301 AACGGTGGCA GCGCGGCGGA ACAGGCGCTG ATGCTGGCGG CGTGCCAACC GGGTGACGGC
 361 TTCGTGCACT TTGCGCATGG TGATGGTGGC CACTTTGCGC TGGAGGCGCT GGCGAGCAAG
 421 GCGGGTATCG AAATTTTTCA CCTGCCGGTG GACCCGGATA CCCTGCTGAT TGACGTGGAT
 481 CGTCTGGCGG CGCTGGTTGA CGCGCACCCG CGTATCCGTA TTGTTATCCT GGATCAGAGC
 541 TTCAAACTGC GTTGGCAGCC GCTGCGTGCG ATCCGTGATG CGCTGCCGGC GCACTGCACC
 601 CTGACCTACG ACGCGAGCCA CGATGGTGGC CTGGTGATGG GTGGCTGGTT TGACAGCCCG
 661 CTGCGTTGCG GTGCGGATGT GGTTCACGGT AACACCCACA AGACCATTGC GGGCCCGCAG
 721 AAAGCGTATG TGGCGTTTGG TAGCGCGGAG CACCCGCTGC TGACCGACAC CAGCATTTGG
 781 GTTTGCCCGA ACATCCAGAG CAACTGCCAC GCGGAACAAC TGCCGAGCAT GTGGGTTGCG
 841 CTGAAAGAGA TCGAAGCGTA CGGTCCGGCG TATGCGAGCC AAGTGGTTCA CAACGCGGCG
 901 GCGTTTGCGC GTGCGCTGCA TGCGCGTGGC CTGGATGTGA GCGGTGAGAG CTTCGGTTTT
 961 ACCGAAACCC ACCAAGTGCA CTTTAGCGTT GGTACCCCGG AGGATGCGCT GCTGACCTGC
1021 CGTGATGTTC TGCACCGTGG TGGCATTCGT ACCACCAACA TTGAACTGCC GGGCAAGCCG
1081 GGCGTGCACG GTATTCGTCT GGGCGTTCAG GCGATGACCC GTCGTGGTAT GGTGGAGCGT
1141 GACTTCGAAA CCGTTGCGGA TTTTATCGCG GCGCTGTGCA CCCGTAAACG TACCCCGGAG
1201 GAAGTGGCGC CGGATGTTGC GACCTTCCTG GGTGATTTTC CGCTGAGCCC GCTGGCGTTC
1261 AGCTTTGACG GTGGCATGAC CGATGAGCTG CGTACCGCGC TGCGTCAAGG TGTTATGCGT
1321 TGA
```

Supplementary Figure 16. ***B. diffusa obiH*** **codon optimized sequence.**

```
  1 ACCGCGACCC CGACCACCGC GGGTCTGACC CGTGAGCGTT TCCTGGCGGA CGTGGCGAAC
 61 GTTGCGCGTG TGGATGTTAG CGCGATGAGC GATGATCTGG CGCCGTTTGA AGCGGGTCTG
121 GACAGCCTGC GTCTGCTGGT GCTGATCGAT GGTTGGCGTA AGCTGGGCGT TGAACTGGGT
181 TTTGGTGAAC TGGCGGAACG TCGTACCCTG GGTGACTGGT GGGCGCTGAT TGAGGCGCGT
241 GAACGTGGCC GTGCGTGA
```

Supplementary Figure 17. ***B. diffusa obiD*** **codon optimized sequence.**

Supplementary Table 1. Structural Alignments with BdObiF with prior structures[a]

Condensation Domain, residues 4-439

Protein	PDB	#Res	# Aligned	RMSD (Å)	Seq ID %
EntF	5JA1	1231	357	2.63	26.6
SrfA-C	2VSQ	1274	350	3.39	16.0
AB3403	4ZXH	1314	369	2.68	16.0
CDA	5DU9	427	407	1.57	32.7
VibH	1L5A	424	349	2.73	25.5
TycC Epimerization	5M6P	438	369	3.15	16.3

Adenylation Domain, residues 444-972

Protein	PDB	#Res	# Aligned	RMSD (Å)	Seq ID %
EntF	5JA1	1321	390	1.92	31.5
SrfA-C	2VSQ	1274	402	1.92	33.6
AB3403	4ZXH	1314	457	2.14	33.9
DhbF	5U89	1039	380	2.12	37.1
Thr1	5N9W	477	448	2.12	38.8
SlgN1	4GR5	451	349	1.65	38.4
PheA	1AMU	510	443	1.87	35.6
LgrA	5ES5.B	685	466	1.92	36.1
Engineered TycA	5N82	396	344	1.68	32.6

Thioesterase Domain, residues 1059-1303

Protein	PDB	#Res	# Aligned	RMSD (Å)	Seq ID %
EntF	5JA1	1231	178	2.47	15.7
SrfA-C	2VSQ	1274	165	2.54	18.2
AB3403	4ZXH	1314	184	2.78	16.8
RifR	3FLB	245	215	2.32	25.6
RedJ	3QMV	244	205	2.23	21.5
Vlm2	6ECB	263	185	2.45	25.9
TesA	6FVJ	209	166	1.96	25.3

[a]Sequence alignments were performed with the SSM algorithm[3] implemented with COOT[4]. The total number of residues (#Res) as well as the number used in the final alignment (#Aligned) are reported, with RMS displacement of Cα positions and sequence identity over the aligned region.

Supplementary Table 2. Data collection and refinement statistics.

**Data collection**	**BD-ObiF1 (6N8E)**
**Beamline**	SSRL 12-2
**Wavelength (Å)**	0.97946
**Resolution range**	39.70-3.00 (3.11-3.00)
**Space group**	$P2_12_12_1$
**a / b / c (Å)**	81.3 154.3 183.9
**α / β / γ (°)**	90 90 90
**Total reflections**	300715 (30159)
**Unique reflections**	47131 (4545)
**Multiplicity**	6.4 (6.6)
**Completeness (%)**	99.9 (99.9)
**Mean I/sigma(I)**	11.4 (3.3)
**Wilson B-factor**	65.0
**R_{sym}**	0.096 (0.407)
**R_{meas}**	0.105 (0.442)
**R_{pim}**	0.041 (0.171)
**$CC_{1/2}$**	0.995 (0.938)
**Structure refinement**	
**Reflections used in refinement**	47061 (4623)
**Reflections used for R_{free}**	1998 (197)
**R_{work}**	0.210 (0.307)
**R_{free}**	0.240 (0.349)
**Number of non-hydrogen atoms**	10018
**macromolecules**	9951
**ligands**	56
**solvent**	11
**Protein residues**	1332
**RMS(bonds)**	0.009
**RMS(angles)**	1.37
**Ramachandran favored (%)**	95.2
**Ramachandran allowed (%)**	4.5
**Ramachandran outliers (%)**	0.3
**Rotamer outliers (%)**	0.2
**Clashscore**	3.9
**Average B-factor**	73.0
**macromolecules**	73.0
**ligands**	84.7
**solvent**	33.5
**Number of TLS groups**	9

Statistics for the highest-resolution shell are shown in parentheses.

Supplementary Table 3. PCR primers used for BD-ObiF1 mutagenesis.

Primer	Nucleotide sequence (5' -> 3')
BD_ObiF1-R1190A-F	GGT GCG **GCA** AGC GGC GAG AAC ATC GTG GAT TTC CTG
BD_ObiF1-R1190A-R	GCC GCT **TGC** CGC ACC CAC TTC GTC CAG AAC CGC ATC
BD_ObiF1-R1263A-F	GCG CGT **GCA** TAT CTG GAC TGG CGT CGT TAC AGC GAT G
BD_ObiF1-R1263A-R	CAG ATA **TGC** ACG CGC ATA ATC CGG GGT CAG CGG GTC
BD_ObiF1-D1254A-F	GGC CAA **GCA** CCG CTG ACC CCG GAT TAT GCG CGT CGT TAT C
BD_ObiF1-D1254A-R	CAG CGG **TGC** TTG GCC ACC GAT AAC CAC GTG CAG C
BD_ObiF1-G1173D-F	G ATT GGT **GAC** ATG CTG CTG GAT GAA GAC GCG GAT G
BD_ObiF1-G1173D-R	CAG CAT **GTC** ACC AAT CGC CAG ACC GAT AAC GTT CG
BD_ObiF1-G1173L-F	G ATT GGT **CTG** ATG CTG CTG GAT GAA GAC GCG GAT G
BD_ObiF1-G1173L-R	CAG CAT **CAG** ACC AAT CGC CAG ACC GAT AAC GTT CG
BD_ObiF1-D1177A-F	CTG CTG **GCG** GAA GAC GCG GAT GCG GTT CTG GAC GAA G
BD_ObiF1-D1177A-R	GTC TTC **CGC** CAG CAG CAT GCC ACC AAT CGC CAG AC
BD_ObiF1-H1284A-F	GGT GGC **GCC** TAT TTT GTT ACC GAG CAT GCG GAC AC
BD_ObiF1-H1284A-R	C AAA ATA **GGC** GCC ACC GTC CGG AAT CAC ATC CAG TT
BD_ObiF1-C1146S-F	AC GGT CAC **TCC** GCG GGT AAC GCG CTG G
BD_ObiF1-C1146S-R	TT ACC CGC **GGA** GTG ACC GTA AAC CAC AAT C
BD_ObiF1-C1146A-F	TAC GGT CAC **GCA** GCG GGT AAC GCG CTG GCG GTT
BD_ObiF1-C1146A-R	C GTT ACC CGC **TGC** GTG ACC GTA AAC CAC AAT CG
BD_ObiF1-A841E-F	TTT GTG **GAA** AAC CCG TAC GGT CCG GCG GGT AC
BD_ObiF1-A841E-R	A CGG GTT **TTC** CAC AAA ACG CAC CGC GTC
BD_ObiF1-rbs-1303/4-F	TGA GAA GGA GAT ATA CAT ATG CCG GCG AGC CGT GCG CCG GAG C
BD_ObiF1-rbs-1303/4-R	CAT ATG TAT ATC TCC TTC TCA CAG CCA ACG CGC CGC CAG CAG AC
BD_ObiF1-Δ1304-F	GAG CGT GAA CGT CAG TGA AAG CTT GCG GCC GCA CTC GAG
BD_ObiF1-Δ1304-R	GGC CGC AAG CTT TCA CTG ACG TTC ACG CTC CGG CGC ACG
BD_ObiF1-MLP-1315-NdeI-F	TGA GAA GGA GAT ATA CAT ATG GCG CTG CGT GCG TTC CTG AAC CCG
BD_ObiF1-Δopal-HindIII-R	C CGC AAG CTT AAC GCA GTG TTC CGC GGT CTC

*Mutant codons are bolded

Supplementary References

1. Papadopoulos, J. S. & Agarwala, R. COBALT: constraint-based alignment tool for multiple protein sequences. *Bioinformatics* **23**, 1073-1079 (2007).

2. Schaffer, J. E.; Reck, M. R.; Prasad, N. K. & Wencewicz, T. A. β-lactone formation during product release from a nonribosomal peptide synthetase. *Nat. Chem. Biol.* **13**, 737–744 (2017).

3. Krissinel, E. & Henrick, K. Secondary-structure matching (SSM), a new tool for fast protein structure alignment in three dimensions. *Acta Crystallogr. Sect. D Struct. Biol.* **60**, 2256-2268 (2004).

4. Emsley, P., Lohkamp, B., Scott, W. G., & Cowtan, K. Features and development of Coot. *Acta Crystallogr. Sect. D Struct. Biol.* **66**, 486-501 (2010).
