# Supplementary material for: The structural basis of N-acyl-α-amino-β-lactone formation catalyzed by a nonribosomal peptide synthetase
Source: Nat Commun. 2019 Jul 31;10:3432. doi: 10.1038/s41467-019-11383-7 (PMC6668435; doi:10.1038/s41467-019-11383-7)
Supplement: Supplementary file 3 — Description of Additional Supplementary Files [file 41467_2019_11383_MOESM3_ESM.pdf]

Description of Additional Supplementary Files

File name: Supplementary Data 1

Description: Supplementary Data 1 contains the LCMS chromatograms for the aldehyde substrate screen, the benozoic acid substrate screen, and the mutant enzyme assays. These data support figures 4c, 4d, 6b, and 6c.
