# Supplementary material for: The structural basis of N-acyl-α-amino-β-lactone formation catalyzed by a nonribosomal peptide synthetase
Source: Nat Commun. 2019 Jul 31;10:3432. doi: 10.1038/s41467-019-11383-7 (PMC6668435; doi:10.1038/s41467-019-11383-7)
Supplement: Supplementary file 4 — Supplementary Data 1 [file 41467_2019_11383_MOESM4_ESM.pdf]

The structural basis of *N*-acyl-α-amino-β-lactone formation catalyzed by a nonribosomal peptide synthetase

D. F. Kreitler et al.

Supplementary Data

I. Aldehyde Substrate Screen 2

Scheme 1: Aldehyde substrate screen for coupled ObiHDF1F2 enzyme reactions with 2,3-DHB 3

Table 1: Expected and observed *m/z* values for compounds 2–5 $[M+H]^+$ ions by high-resolution LCMS analysis 4

LCMS chromatograms for aldehyde substrate screen of coupled ObiHDF1F2 enzyme reactions with 2,3-DHB 5–12

II. Benzoic Acid Substrate Screen 13

Scheme 2: Benzoic acid substrate screen for coupled ObiHDF1F2 enzyme reactions with phenylacetaldehyde 14

Table 2: Expected and observed *m/z* values for compounds 2–5 $[M+H]^+$ ions by high-resolution LCMS analysis 15

LCMS chromatograms for benzoic acid substrate screen of coupled ObiHDF1F2 enzyme reactions with phenylacealdehyde 16–36

III. ObiF1 Mutant Assays 37

Scheme 3: ObiF1 mutant assays for coupled ObiHDF1F2 enzyme reactions with 2,3-DHB and phenylacetaldehyde as substrates 38

LCMS chromatograms for coupled ObiHDF1F2 enzyme reactions with mutant ObiF1 and added ObiF1 MLP using 2,3-DHB and phenylacetaldehyde as substrates 39–95

I. Aldehyde Substrate Screen

Scheme 1, Table 1, and the associated LC-MS chromatograms provide the structures of substrates/products, expected *m/z* values for $[M+H]^+$ ions, and raw LCMS data used for generation of ion count heat maps (main text), respectively, for aldehyde substrate screening assays reported in this manuscript. Compound **4–6** letters correspond to variable aldehyde substrate (**1a–h**) and amino acid (**2a–h**) while keeping the benzoic acid substrate (**3a**) constant. (For compounds **4–6**, the first letter in the two-letter nomenclature, **a–h**, represents the structure of R_1 while the second letter in the two-letter nomenclature, **a**, represents the structure of R_2.) All enzyme reactions were performed using the wild-type reconstituted enzymes, ObiH, ObiD, ObiF1, and ObiF2 from *Burkholderia diffusa,* as shown in Scheme 1 following the experimental procedure described in the main text with DTT as the thiol trapping agent. Table 1 contains compound letter identifiers, the molecular formula, the expected $[M+H]^+$, and the observed $[M+H]^+$ from high-resolution LCMS analysis of aliquots from the enzyme reactions at the 3 hour time point. An entry of "NA" indicates that that molecular ion for the expected mass was not observed. All LCMS chromatograms are from low-resolution LCMS analysis of aliquots from the enzyme reactions at 30 minute, 3 hour, and 24 hour time points including the chromatograms for optical absorbance at 220 nm (UV/Vis), total ion counts (TIC), and extracted ion counts (EIC) for the phenylalanine internal standard and compound structures indicated on to the left of each EIC chromatogram. LC-MS samples were prepared by removing a 100 µL aliquot of the assay at 30 minutes, 3 hours, and 24 hours, quenched by acidification to pH 2 with 1 M HCl, addition of phenylalanine at 100 µM as internal standard, and immediately frozen at -80° C. At the time of LC-MS analysis, the sample is thawed, centrifuged at 16,000 rpm for 1 minute to pellet solids, and analyzed by LCMS (instrument: Agilent 6130 quadrupole with G1313 autosampler, G1315 diode array detector, 122 series solvent module; column: Phenomenex Gemini C18, 50 x 2 mm, 5 µm plus guard column; solvents: 0.1% formic acid in (A) water and (B) acetonitrile; method: 5% B to 100% B over 20 min; software: G2710 ChemStation).

Scheme 1: Aldehyde substrate screen for coupled ObiHDF1F2 enzyme reactions with 2,3-DHB (3a).

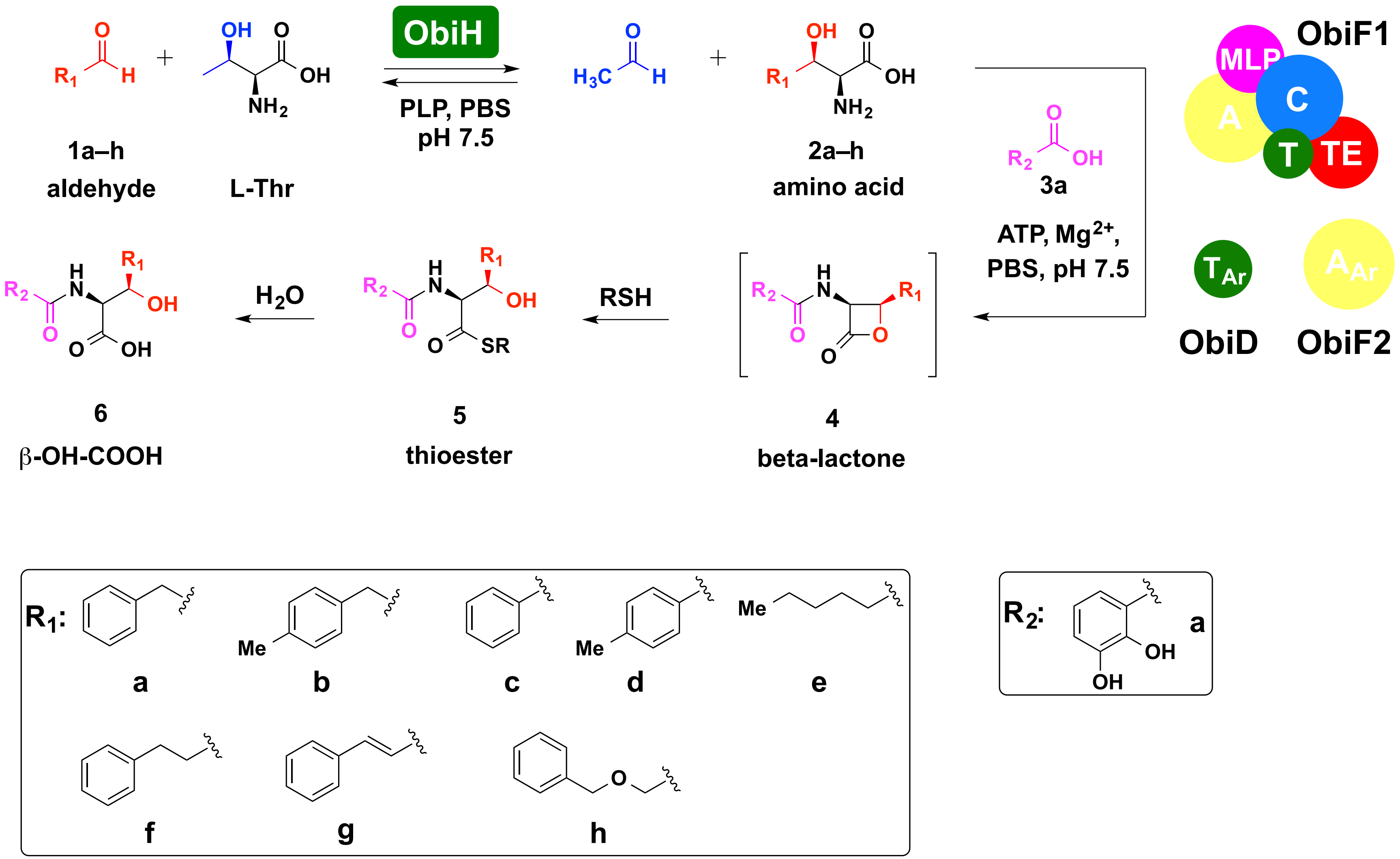

Table 1: Expected and observed *m/z* values for compounds 2a–h, 4, and 6 $[M+H]^+$ ions by high-resolution LCMS analysis.

Compound #	Molecular Formula			Expected $(M+H)^+$			Observed $(M+H)^+$		
	2a–h	4	6	2a–h	4	6	2a–h	4	6
aa	$C_{10}H_{14}NO_3^+$	$C_{17}H_{16}NO_5^+$	$C_{17}H_{18}NO_6^+$	196.0968	314.1023	332.1129	196.0948-196.0988	314.0992-314.1054	332.1096-332.1162
ba	$C_{11}H_{16}NO_3^+$	$C_{18}H_{18}NO_5^+$	$C_{18}H_{20}NO_6^+$	210.1125	328.1179	346.1285	210.1104-210.1146	328.1146-328.1212	346.1250-346.1320
ca	$C_9H_{12}NO_3^+$	$C_{16}H_{14}NO_5^+$	$C_{16}H_{16}NO_6^+$	182.0812	300.0866	318.0972	182.0794-182.0830	NA	NA
da	$C_{10}H_{14}NO_3^+$	$C_{17}H_{16}NO_5^+$	$C_{17}H_{18}NO_6^+$	196.0968	314.1023	332.1129	196.0948-196.0988	314.0992-314.1054	332.1096-332.1162
ea	$C_8H_{18}NO_3^+$	$C_{15}H_{20}NO_5^+$	$C_{15}H_{22}NO_6^+$	176.1281	294.1336	312.1442	176.1263-176.1299	294.1307-294.1365	312.1411-312.1473
fa	$C_{11}H_{16}NO_3^+$	$C_{18}H_{18}NO_5^+$	$C_{18}H_{20}NO_6^+$	210.1125	328.1179	346.1285	210.1104-210.1146	328.1146-328.1212	346.1250-346.1320
ga	$C_{11}H_{14}NO_3^+$	$C_{18}H_{16}NO_5^+$	$C_{18}H_{18}NO_6^+$	208.0968	326.1023	344.1129	208.0947-208.0989	326.0990-326.1056	344.1095-344.1163
ha	$C_{11}H_{16}NO_4^+$	$C_{18}H_{18}NO_6^+$	$C_{18}H_{20}NO_7^+$	226.1074	344.1129	362.1234	226.1051-226.1097	344.1095-344.1163	362.1198-362.1270

LCMS chromatograms for aldehyde substrate screen of coupled ObiHDF1F2 enzyme reactions with 2,3-DHB.

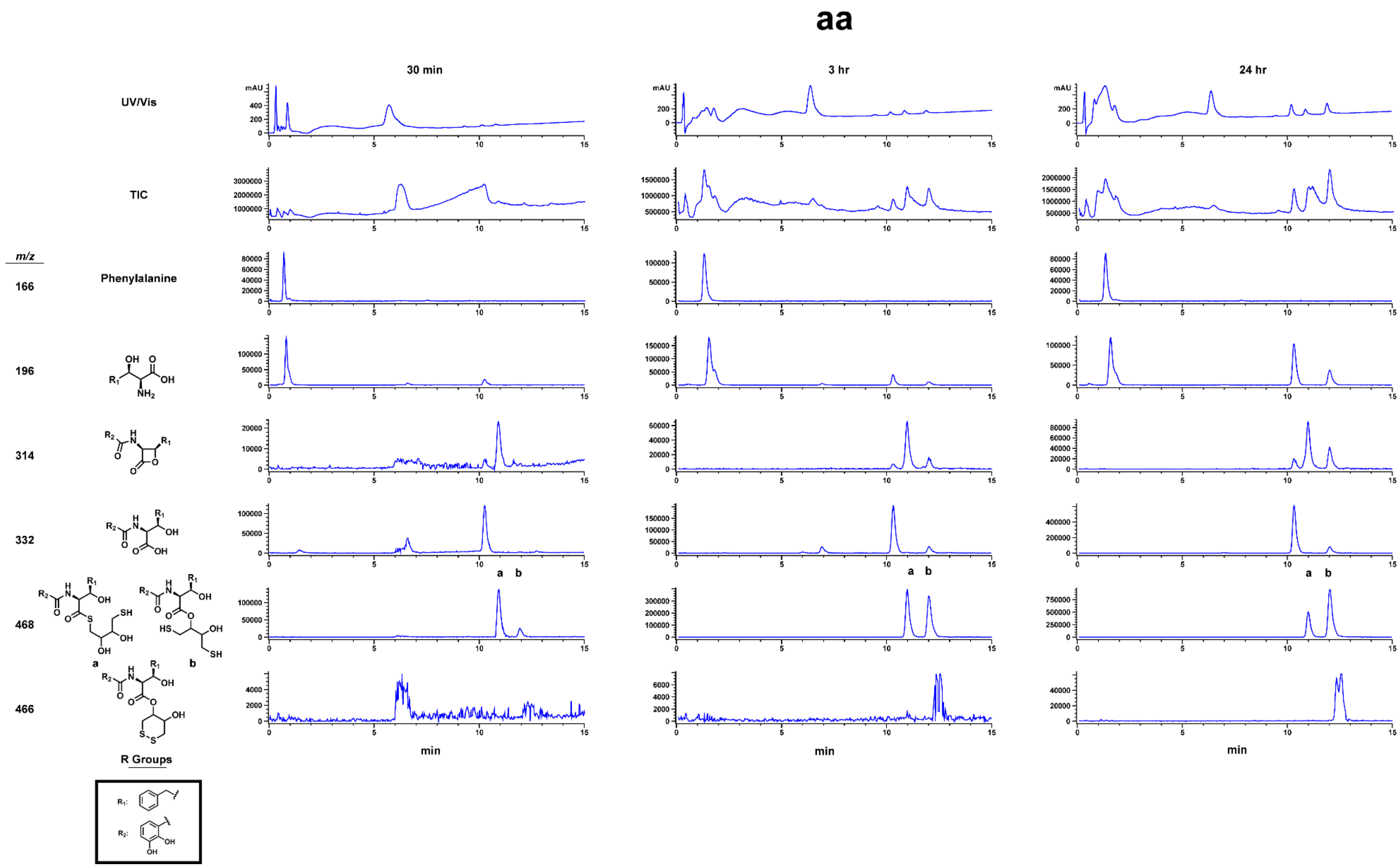

ba

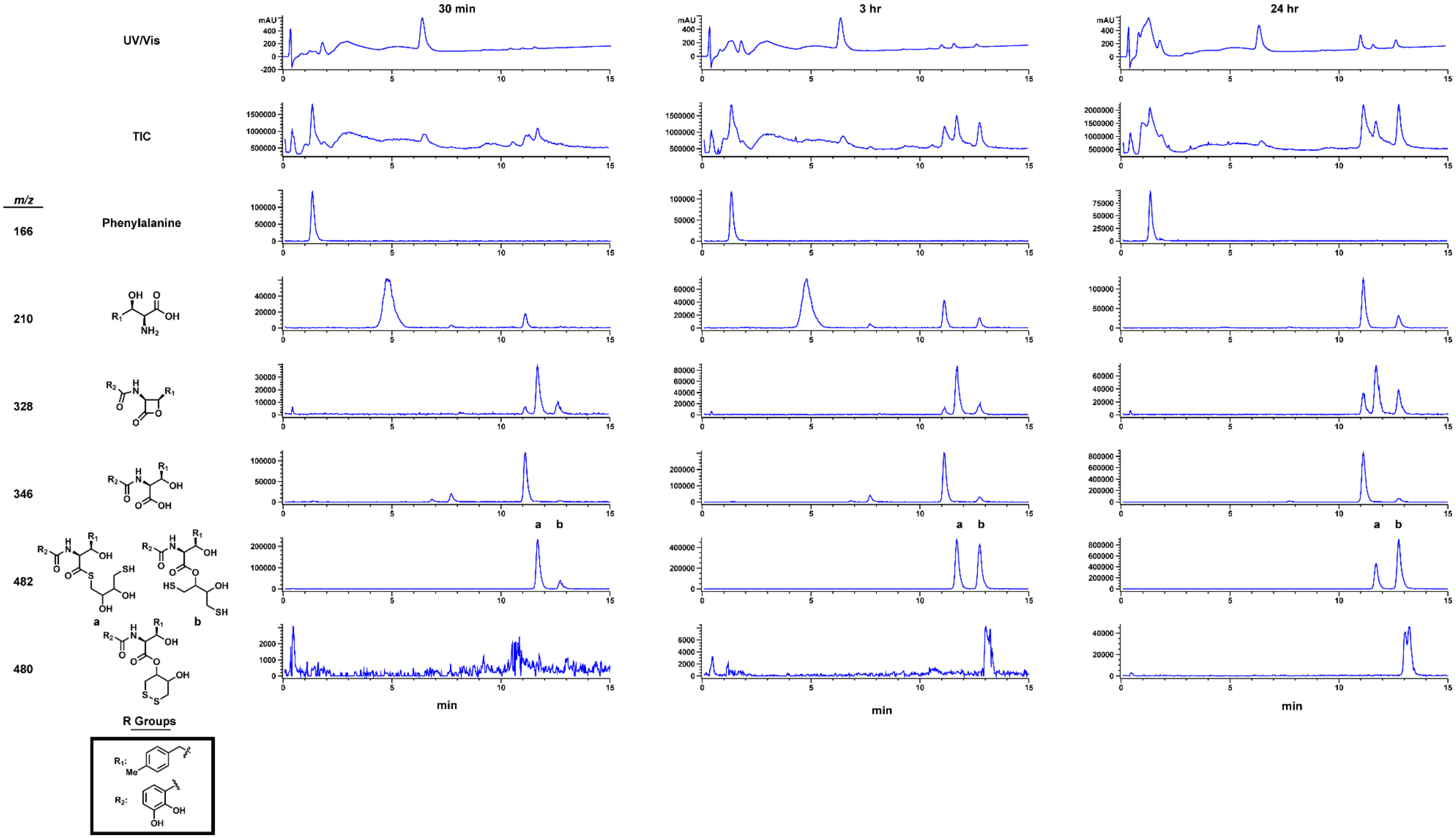
30 min
3 hr
24 hr
UV/Vis
TIC
m/z
166
Phenylalanine
210
328
346
482
480
a
b
min
R Groups

ca

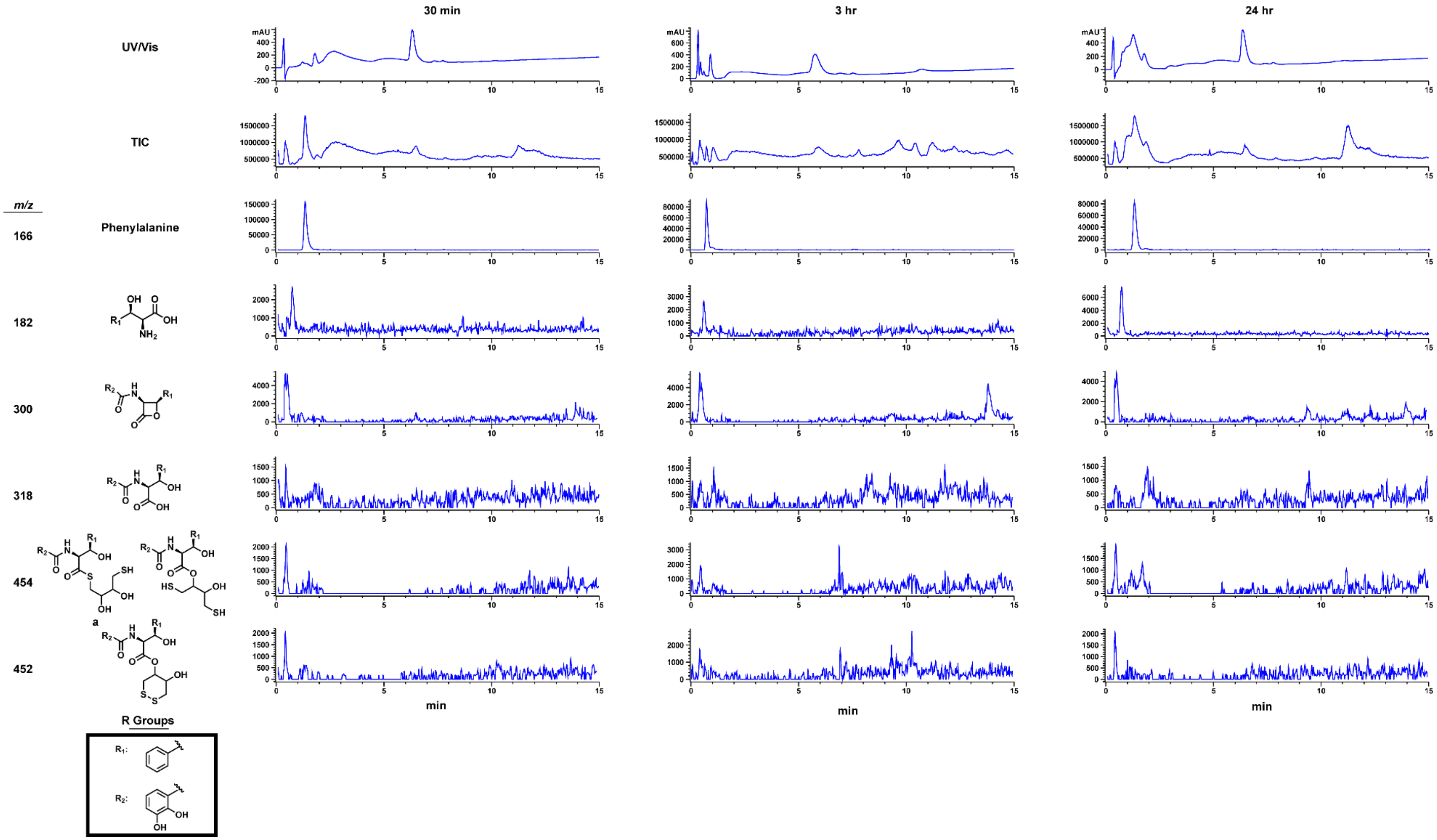
30 min
3 hr
24 hr
UV/Vis
TIC
m/z
166
Phenylalanine
182
300
318
454
452
a
R Groups
min

da

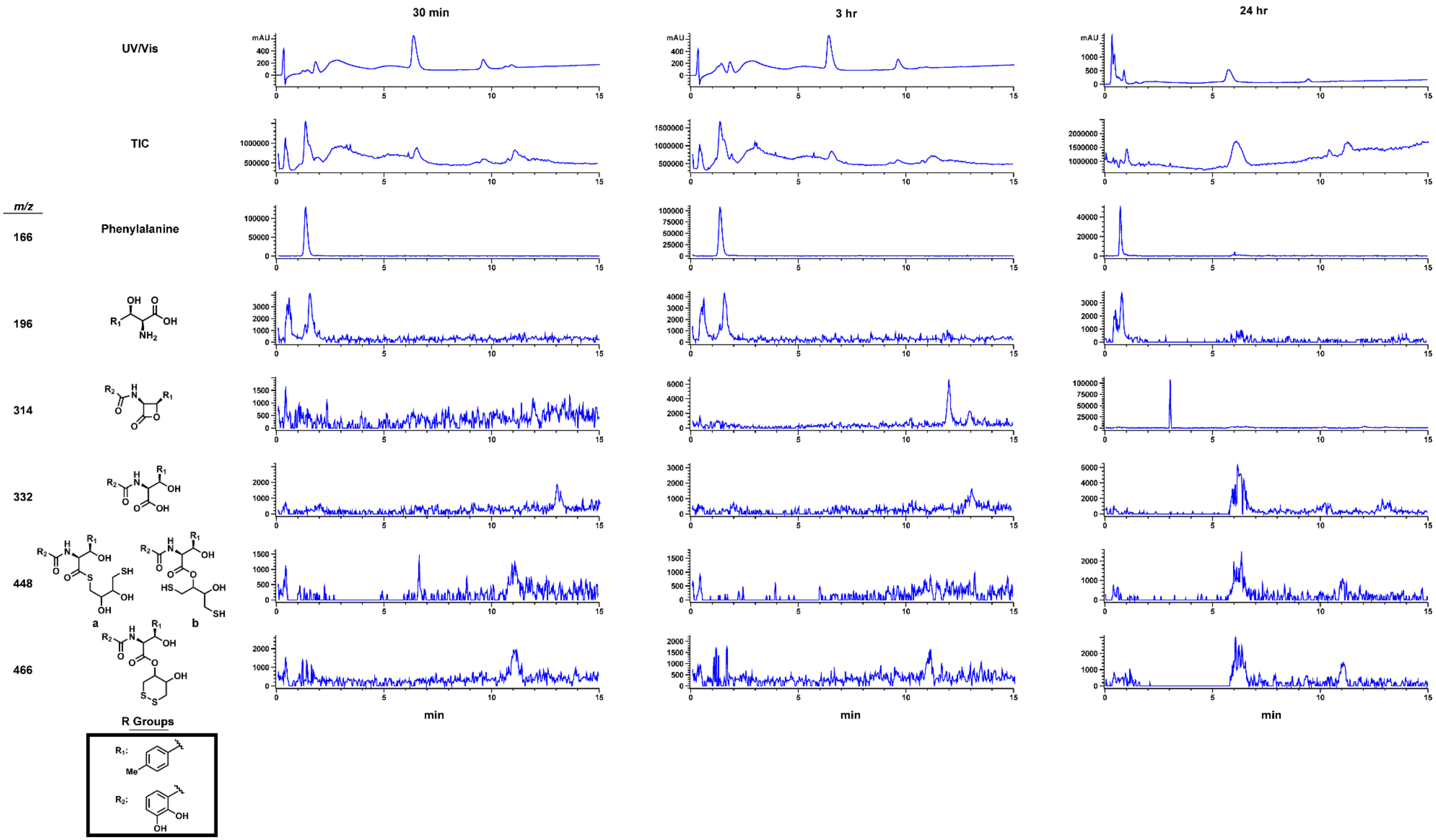
30 min
3 hr
24 hr
UV/Vis
TIC
m/z
166
Phenylalanine
196
314
332
448
466
a
b
R Groups
min

ea

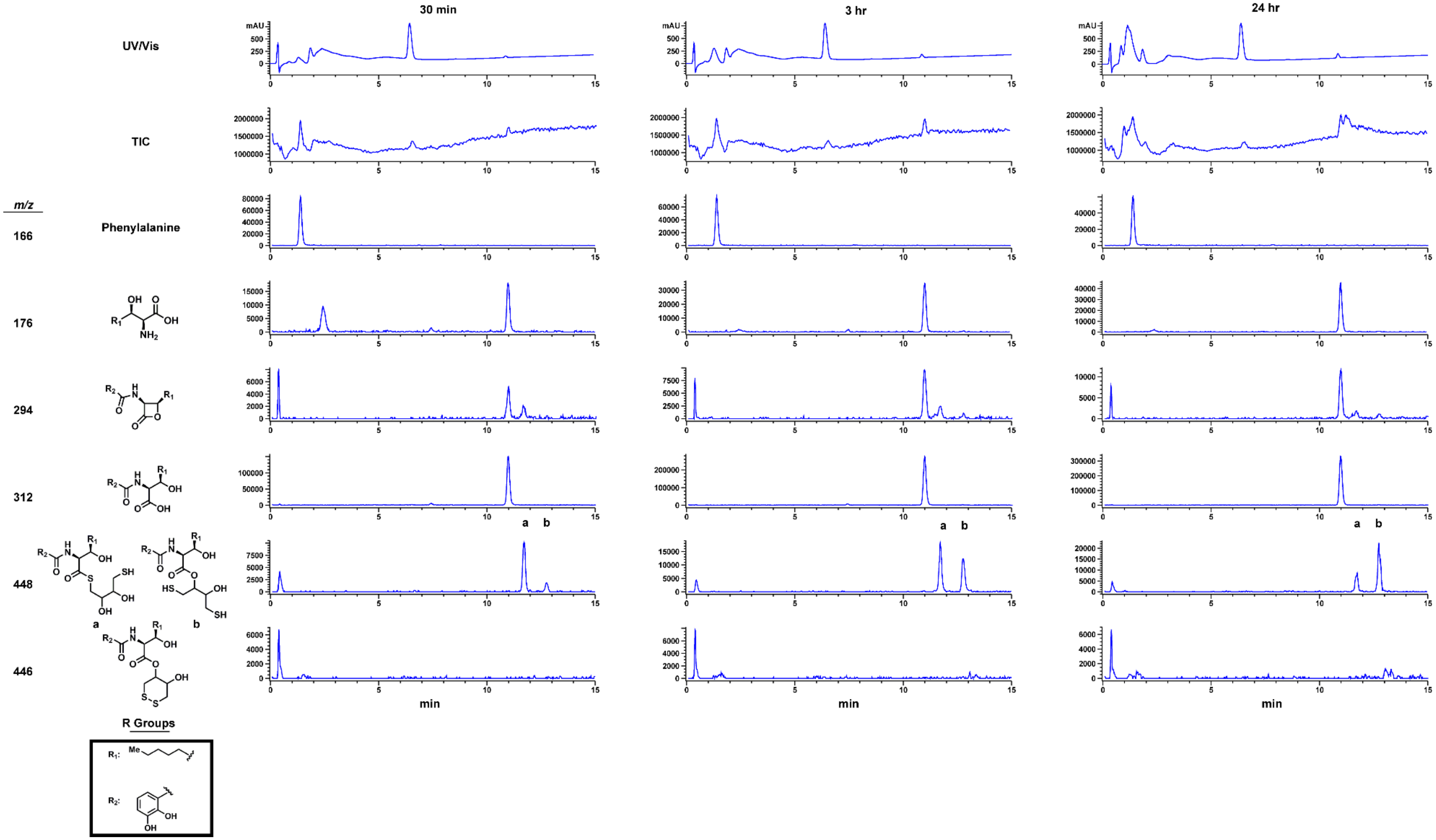
30 min
3 hr
24 hr
UV/Vis
TIC
m/z
166
Phenylalanine
176
294
312
448
446
a
b
min
R Groups

fa

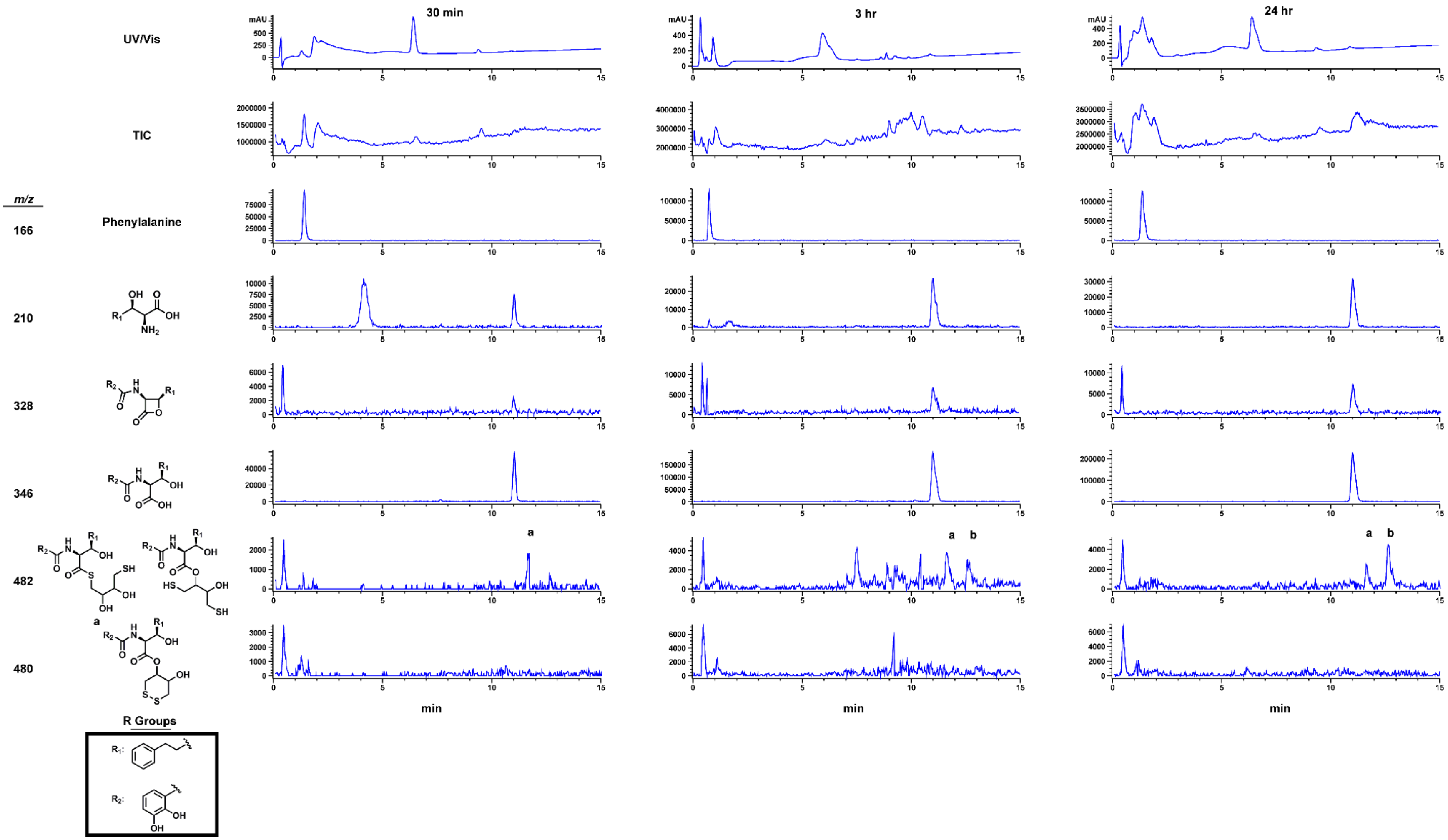
30 min
3 hr
24 hr
UV/Vis
TIC
m/z
166
Phenylalanine
210
328
346
482
480
a
b
R Groups
min

ga

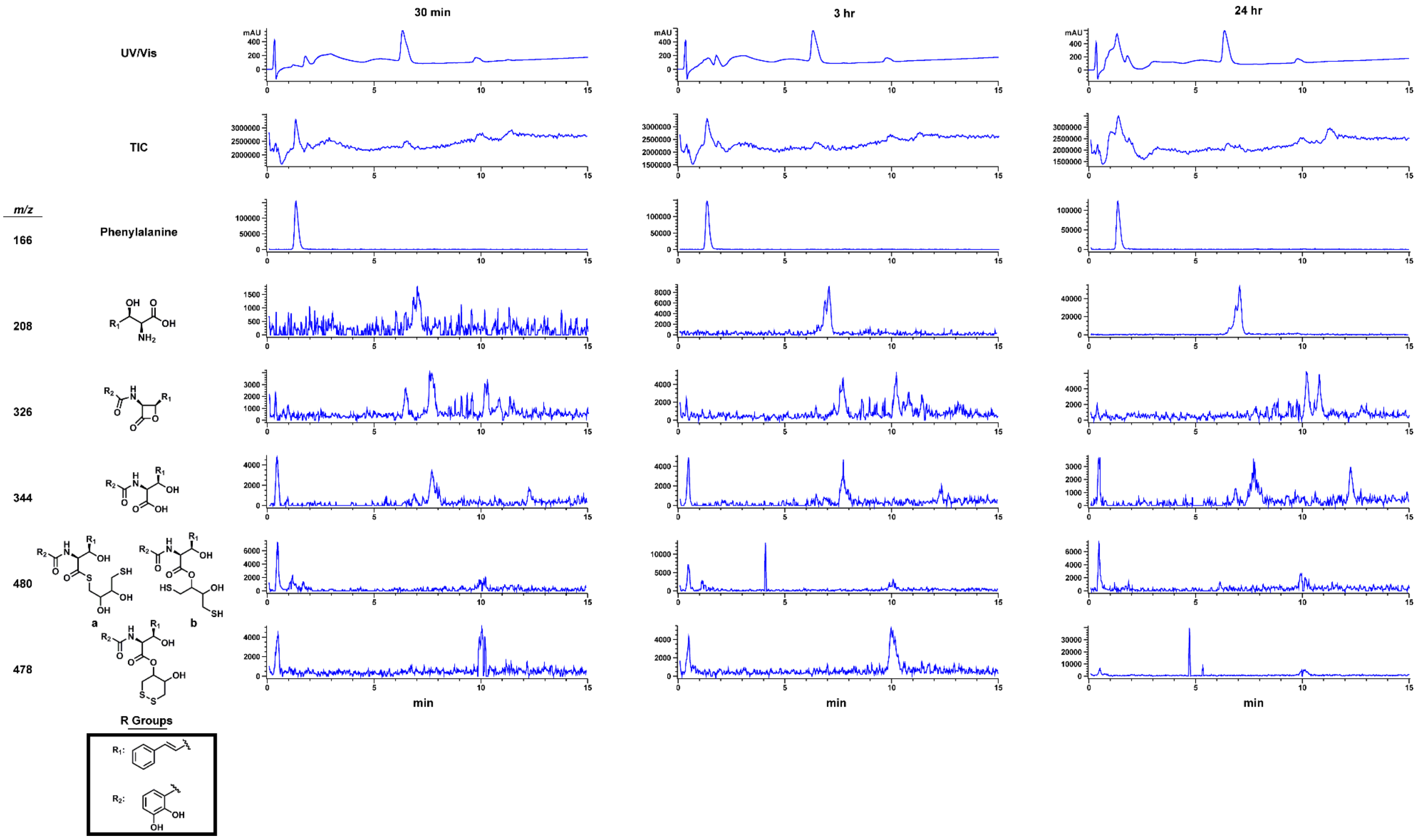
30 min
3 hr
24 hr
UV/Vis
TIC
m/z
166
Phenylalanine
208
326
344
480
a
b
478
R Groups
R1:
R2:
min

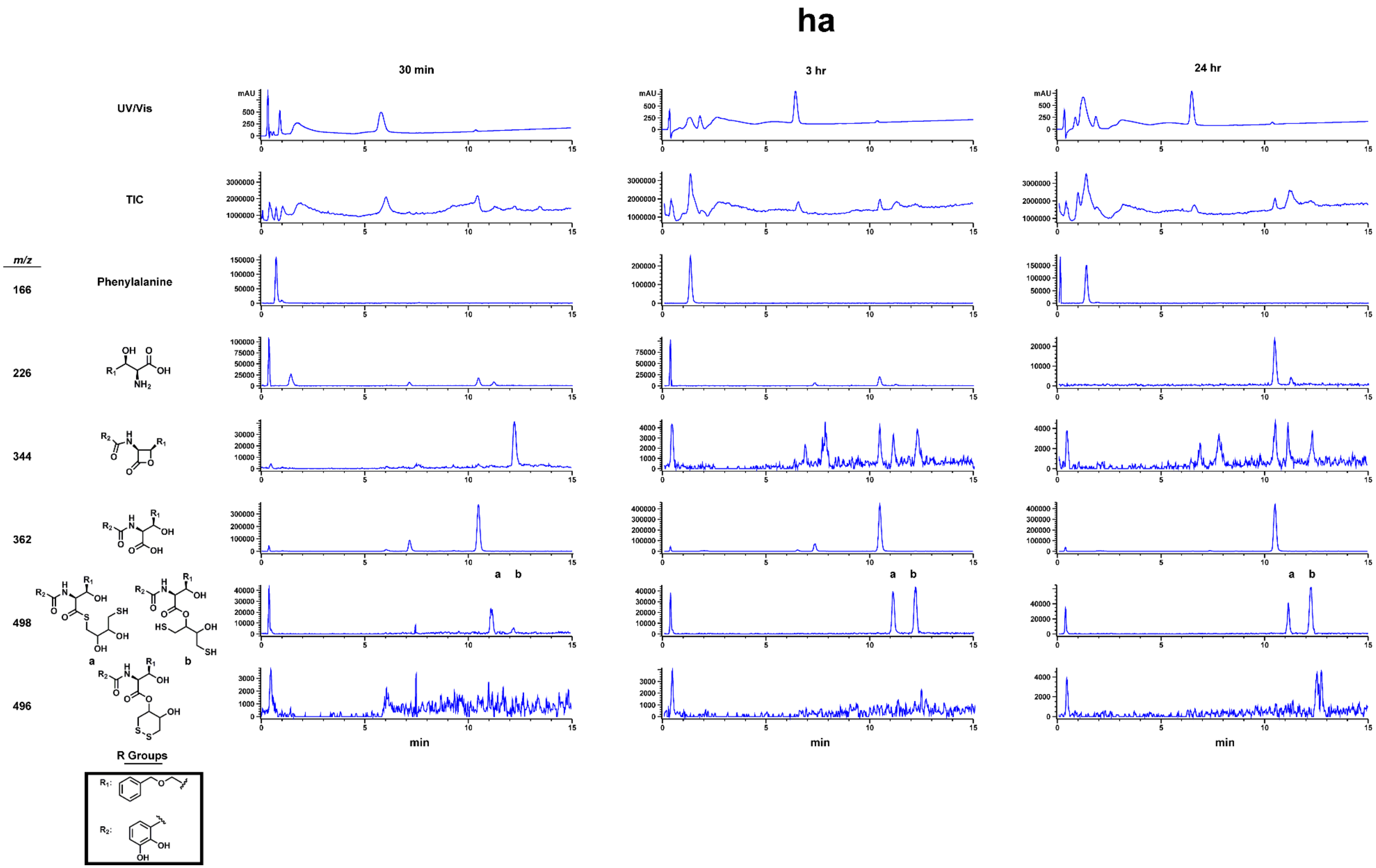

ha
30 min
3 hr
24 hr
UV/Vis
TIC
m/z
166
Phenylalanine
226
344
362
498
496
a
b
R Groups
min

II. Benzoic Acid Substrate Screen

Scheme 1, Table 1, and the associated LC-MS chromatograms provide the structures of substrates/products, expected *m/z* values for $[M+H]^+$ ions, and raw LCMS data used for generation of ion count heat maps (main text), respectively, for benzoic acid substrate screening assays reported in this manuscript. Compound **4–6** letters correspond to constant aldehyde substrate (**1a**) and amino acid (**2a**) while varying the benzoic acid substrate (**3a–u**) constant. (For compounds **4–6**, the first letter in the two-letter nomenclature, **a–h**, represents the structure of R_1 while the second letter in the two-letter nomenclature, **a**, represents the structure of R_2.) All enzyme reactions were performed using the wild-type reconstituted enzymes, ObiH, ObiD, ObiF1, and ObiF2 from *Burkholderia diffusa,* as shown in Scheme 2 following the experimental procedure described in the main text with DTT as the thiol trapping agent. Table 1 contains compound letter identifiers, the molecular formula, the expected $[M+H]^+$, and the observed $[M+H]^+$ from high-resolution LCMS analysis of aliquots from the enzyme reactions at the 3 hour time point. An entry of "NA" indicates that that molecular ion for the expected mass was not observed. All LCMS chromatograms are from low-resolution LCMS analysis of aliquots from the enzyme reactions at 30 minute, 3 hour, and 24 hour time points including the chromatograms for optical absorbance at 220 nm (UV/Vis), total ion counts (TIC), and extracted ion counts (EIC) for the phenylalanine internal standard and compound structures indicated on to the left of each EIC chromatogram. LC-MS samples were prepared by removing a 100 µL aliquot of the assay at 30 minutes, 3 hours, and 24 hours, quenched by acidification to pH 2 with 1 M HCl, addition of phenylalanine at 100 µM as internal standard, and immediately frozen at -80° C. At the time of LC-MS analysis, the sample is thawed, centrifuged at 16,000 rpm for 1 minute to pellet solids, and analyzed by LCMS (instrument: Agilent 6130 quadrupole with G1313 autosampler, G1315 diode array detector, 122 series solvent module; column: Phenomenex Gemini C18, 50 x 2 mm, 5 µm plus guard column; solvents: 0.1% formic acid in (A) water and (B) acetonitrile; method: 5% B to 100% B over 20 min; software: G2710 ChemStation). ***Note: EIC traces and *m/z* values highlighted in red correspond to $[M+Na]^+$ molecular ions.**

Scheme 2: Benzoic acid substrate screen for coupled ObiHDF1F2 enzyme reactions with phenylacetaldehyde (1a).

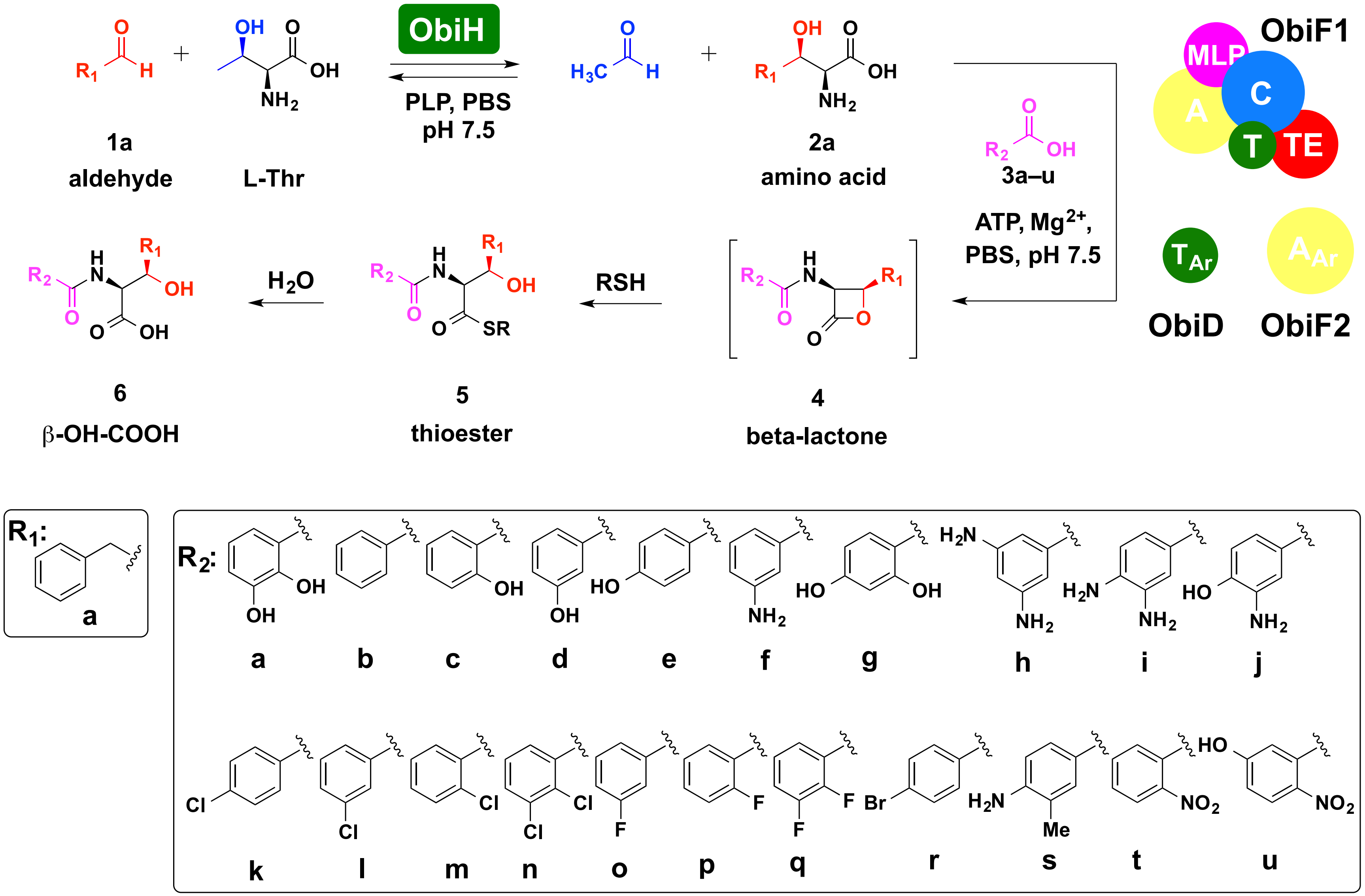

Table 2: Expected and observed *m/z* values for compounds 2a, 4, and 6 [M+H]+ ions by high-resolution LCMS analysis. *Note: *m/z* values highlighted in red correspond to [M+Na]+ molecular ions.

Compound #	Molecular Formula			Expected (M+H)+			Observed (M+H)+		
	2a	4	6	2a	4	6	2a	4	6
aa	$C_{10}H_{14}NO_3^+$	$C_{17}H_{16}NO_5^+$	$C_{17}H_{18}NO_6^+$	196.0968	314.1023	332.1129	196.0948-196.0988	314.0992-314.1054	332.1096-332.1162
ab	$C_{10}H_{14}NO_3^+$	$C_{17}H_{16}NO_3^+$	$C_{17}H_{18}NO_4^+$	196.0968	282.1125	300.1230	196.0948-196.0988	282.1097-282.1153	300.1200-300.1260
ac	$C_{10}H_{14}NO_3^+$	$C_{17}H_{16}NO_4^+$	$C_{17}H_{18}NO_5^+$	196.0968	298.1074	316.1179	196.0948-196.0988	282.1097-282.1153	300.1200-300.1260
ad	$C_{10}H_{14}NO_3^+$	$C_{17}H_{16}NO_4^+$	$C_{17}H_{18}NO_5^+$	196.0968	298.1074	316.1179	196.0948-196.0988	282.1097-282.1153	300.1200-300.1260
ae	$C_{10}H_{14}NO_3^+$	$C_{17}H_{16}NO_4^+$	$C_{17}H_{18}NO_5^+$	196.0968	298.1074	316.1179	196.0948-196.0988	NA	300.1200-300.1260
af	$C_{10}H_{14}NO_3^+$	$C_{17}H_{17}N_2O_3^+$	$C_{17}H_{19}N_2O_4^+$	196.0968	297.1234	315.1339	196.0948-196.0988	297.1204-297.1264	315.1307-315.1371
ag	$C_{10}H_{14}NO_3^+$	$C_{17}H_{16}NO_5^+$	$C_{17}H_{18}NO_6^+$	196.0968	314.1023	332.1129	196.0948-196.0988	314.0992-314.1054	332.1096-332.1162
ah	$C_{10}H_{14}NO_3^+$	$C_{17}H_{18}N_3O_3^+$	$C_{17}H_{20}N_3O_4^+$	196.0968	334.1162	330.1448	196.0948-196.0988	NA	NA
ai	$C_{10}H_{14}NO_3^+$	$C_{17}H_{18}N_3O_3^+$	$C_{17}H_{20}N_3O_4^+$	196.0968	334.1162	330.1448	196.0948-196.0988	NA	NA
aj	$C_{10}H_{14}NO_3^+$	$C_{17}H_{17}N_2O_4^+$	$C_{17}H_{19}N_2O_5^+$	196.0968	313.1183	331.1288	196.0948-196.0988	NA	NA
ak	$C_{10}H_{14}NO_3^+$	$C_{17}H_{15}ClNO_3^+$	$C_{17}H_{17}ClNO_4^+$	196.0968	316.0735	334.0841	196.0948-196.0988	NA	334.0808-334.0874
al	$C_{10}H_{14}NO_3^+$	$C_{17}H_{15}ClNO_3^+$	$C_{17}H_{17}ClNO_4^+$	196.0968	316.0735	334.0841	196.0948-196.0988	316.0703-316.0767	334.0808-334.0874
am	$C_{10}H_{14}NO_3^+$	$C_{17}H_{15}ClNO_3^+$	$C_{17}H_{17}ClNO_4^+$	196.0968	316.0735	334.0841	196.0948-196.0988	316.0703-316.0767	334.0808-334.0874
an	$C_{10}H_{14}NO_3^+$	$C_{17}H_{14}Cl_2NO_3^+$	$C_{17}H_{16}Cl_2NO_4^+$	196.0968	350.0345	368.0451	196.0948-196.0988	NA	368.0414-368.0488
ao	$C_{10}H_{14}NO_3^+$	$C_{17}H_{15}FNO_3^+$	$C_{17}H_{17}FNO_4^+$	196.0968	300.1030	318.1136	196.0948-196.0988	300.1000-300.1060	318.1104-318.1168
ap	$C_{10}H_{14}NO_3^+$	$C_{17}H_{15}FNO_3^+$	$C_{17}H_{17}FNO_4^+$	196.0968	300.1030	318.1136	196.0948-196.0988	300.1000-300.1060	318.1104-318.1168
aq	$C_{10}H_{14}NO_3^+$	$C_{17}H_{14}F_2NO_3^+$	$C_{17}H_{16}F_2NO_4^+$	196.0968	318.0936	336.1042	196.0948-196.0988	318.0904-318.0968	336.1008-336.1076
ar	$C_{10}H_{14}NO_3^+$	$C_{17}H_{15}BrNO_3^+$	$C_{17}H_{17}BrNO_4^+$	196.0968	360.0230	378.0335	196.0948-196.0988	NA	NA
as	$C_{10}H_{14}NO_3^+$	$C_{18}H_{19}N_2O_3^+$	$C_{18}H_{21}N_2O_4^+$	196.0968	333.1210	329.1496	196.0948-196.0988	NA	329.1463-329.1529
at	$C_{10}H_{14}NO_3^+$	$C_{17}H_{15}N_2O_5^+$	$C_{17}H_{17}N_2O_6^+$	196.0968	327.0975	345.1081	196.0948-196.0988	NA	345.1049-345.1119
au	$C_{10}H_{14}NO_3^+$	$C_{17}H_{15}N_2O_6^+$	$C_{17}H_{17}N_2O_7^+$	196.0968	343.0925	361.1030	196.0948-196.0988	NA	NA

LCMS chromatograms for benzoic acid substrate screen of coupled ObiHDF1F2 enzyme reactions with phenylacetaldehyde (1a). *Note: EIC traces and *m/z* values highlighted in red correspond to $[M+Na]^+$ molecular ions.

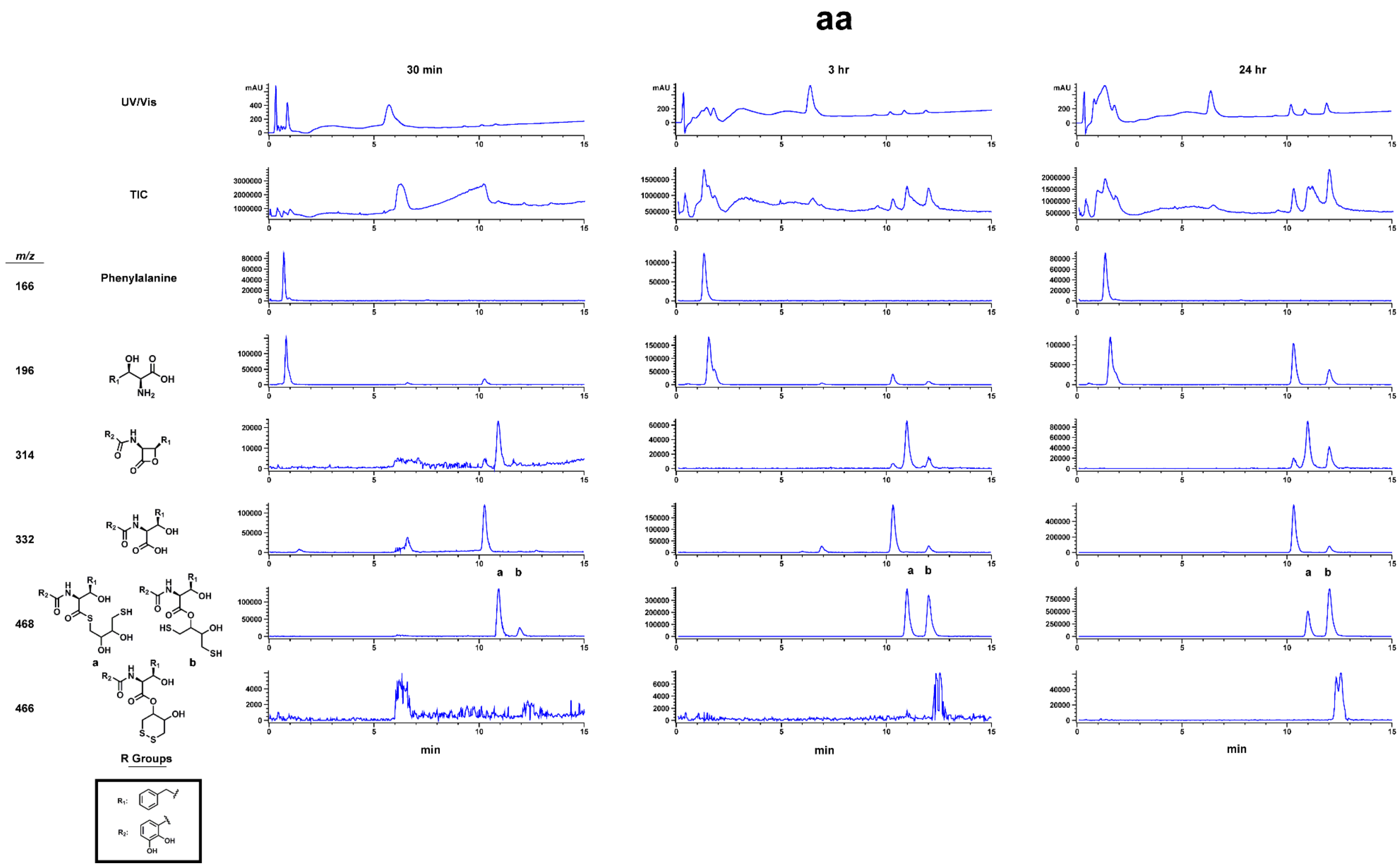

ab

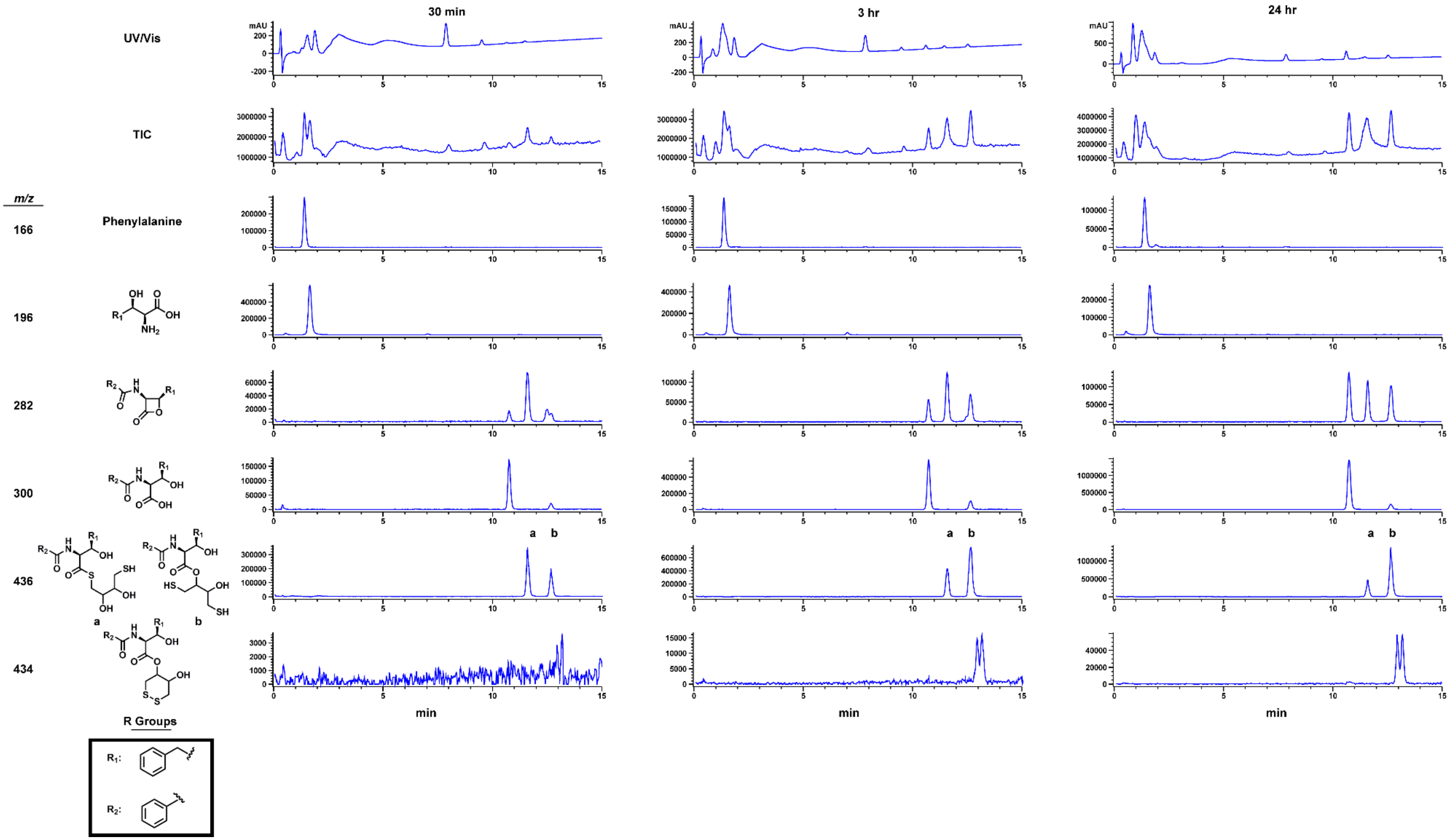

30 min
3 hr
24 hr
UV/Vis
TIC
m/z
166
Phenylalanine
196
282
300
436
434
a
b
R Groups
R1:
R2:
min

ac

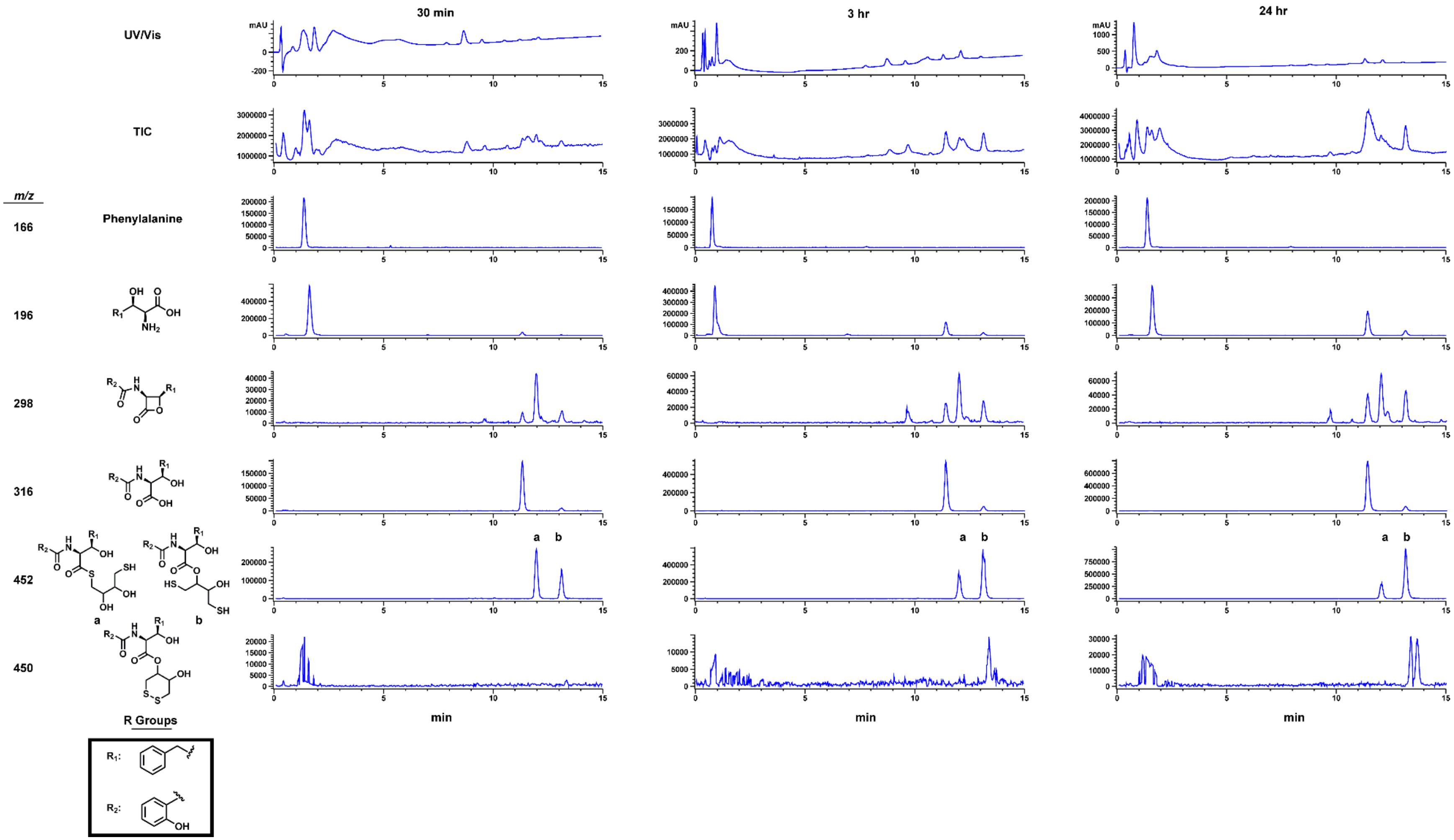

30 min
3 hr
24 hr
UV/Vis
TIC
m/z
166
Phenylalanine
196
298
316
452
450
a
b
mAU
min
R Groups
R1:
R2:

ad

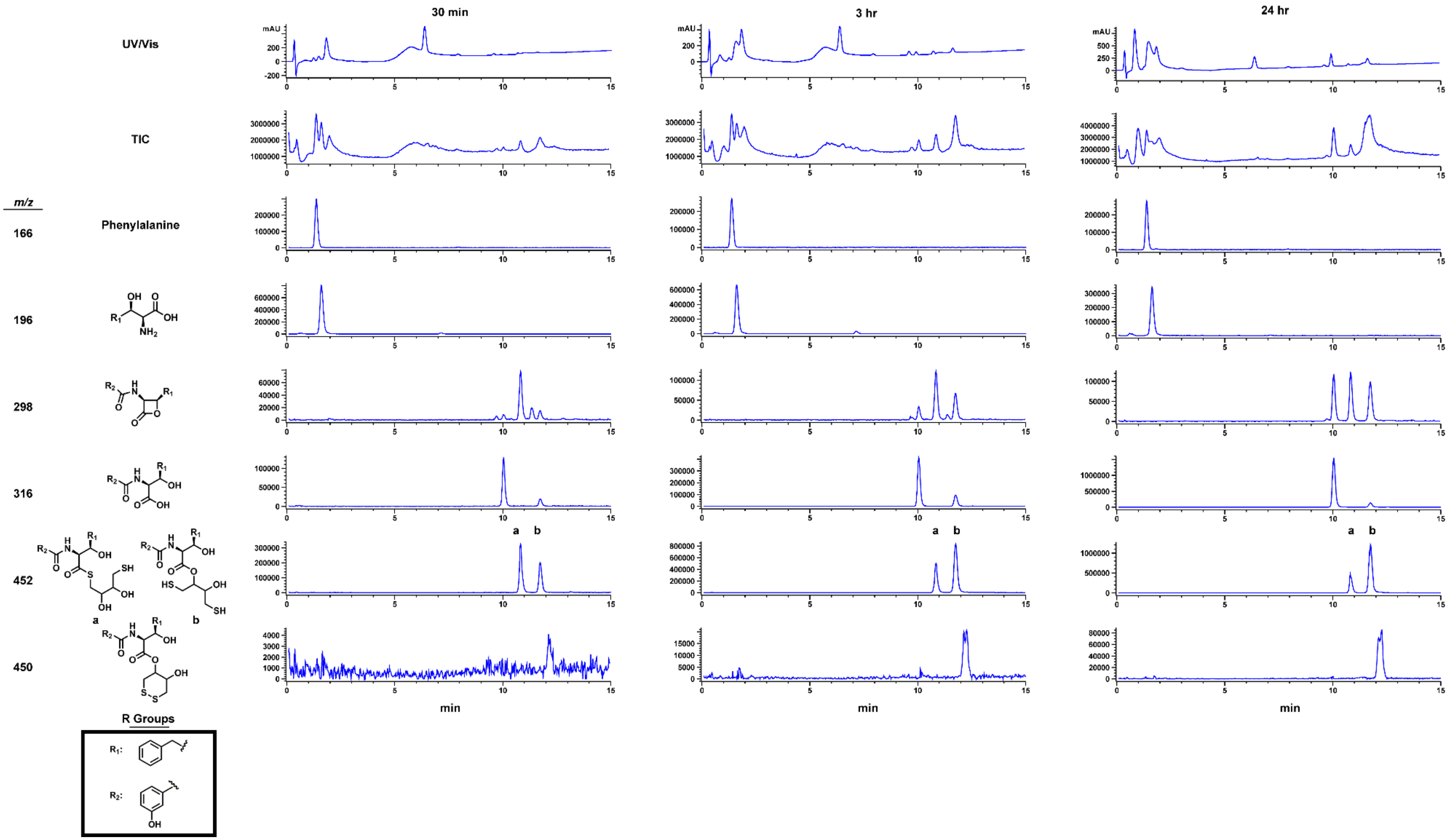
30 min
3 hr
24 hr
UV/Vis
TIC
m/z
166
Phenylalanine
196
298
316
452
a
b
450
R Groups
R1:
R2:
OH
mAU
min

ae

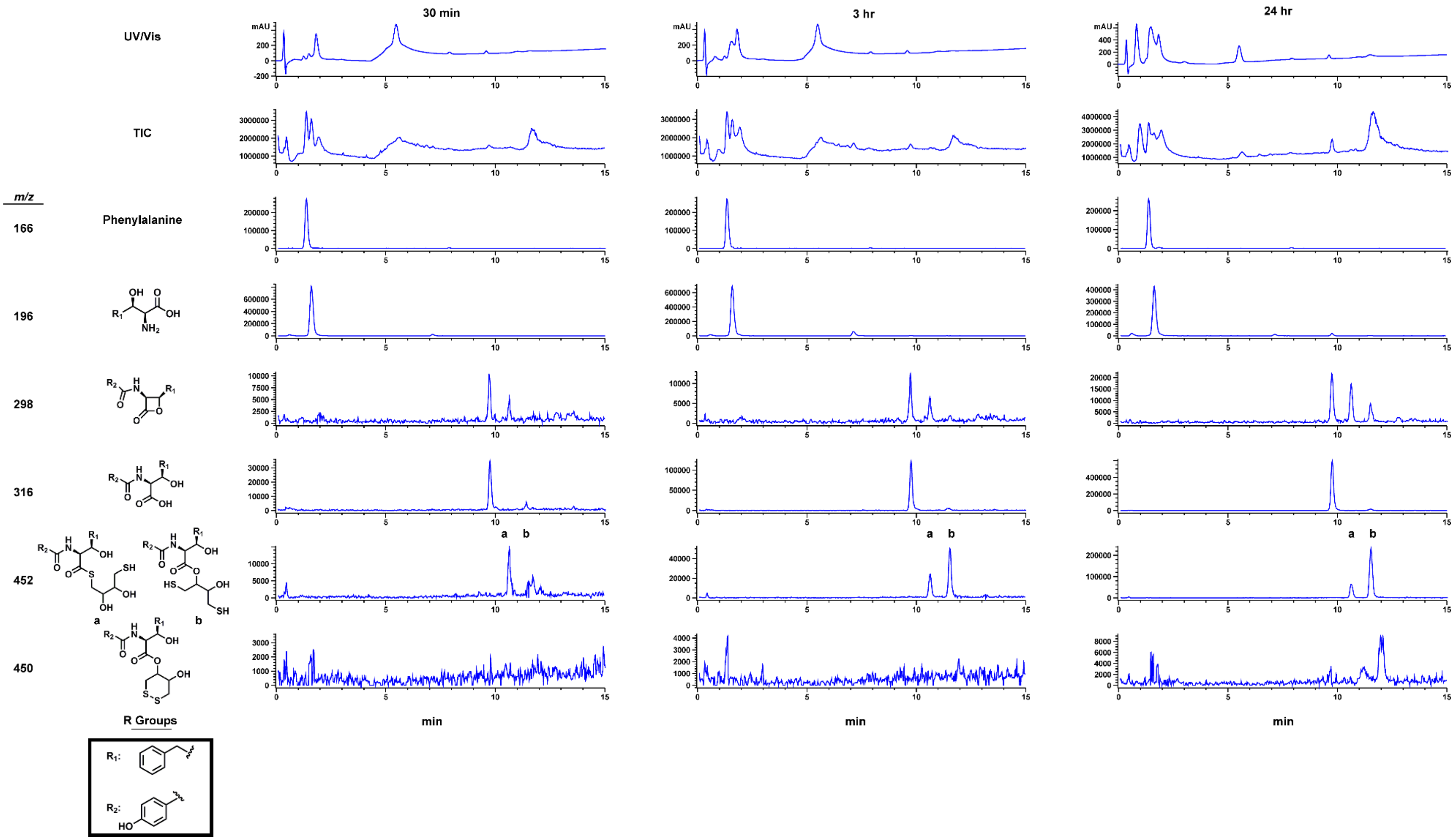

30 min
3 hr
24 hr
UV/Vis
TIC
m/z
166
Phenylalanine
196
298
316
452
450
a
b
min
R Groups

af

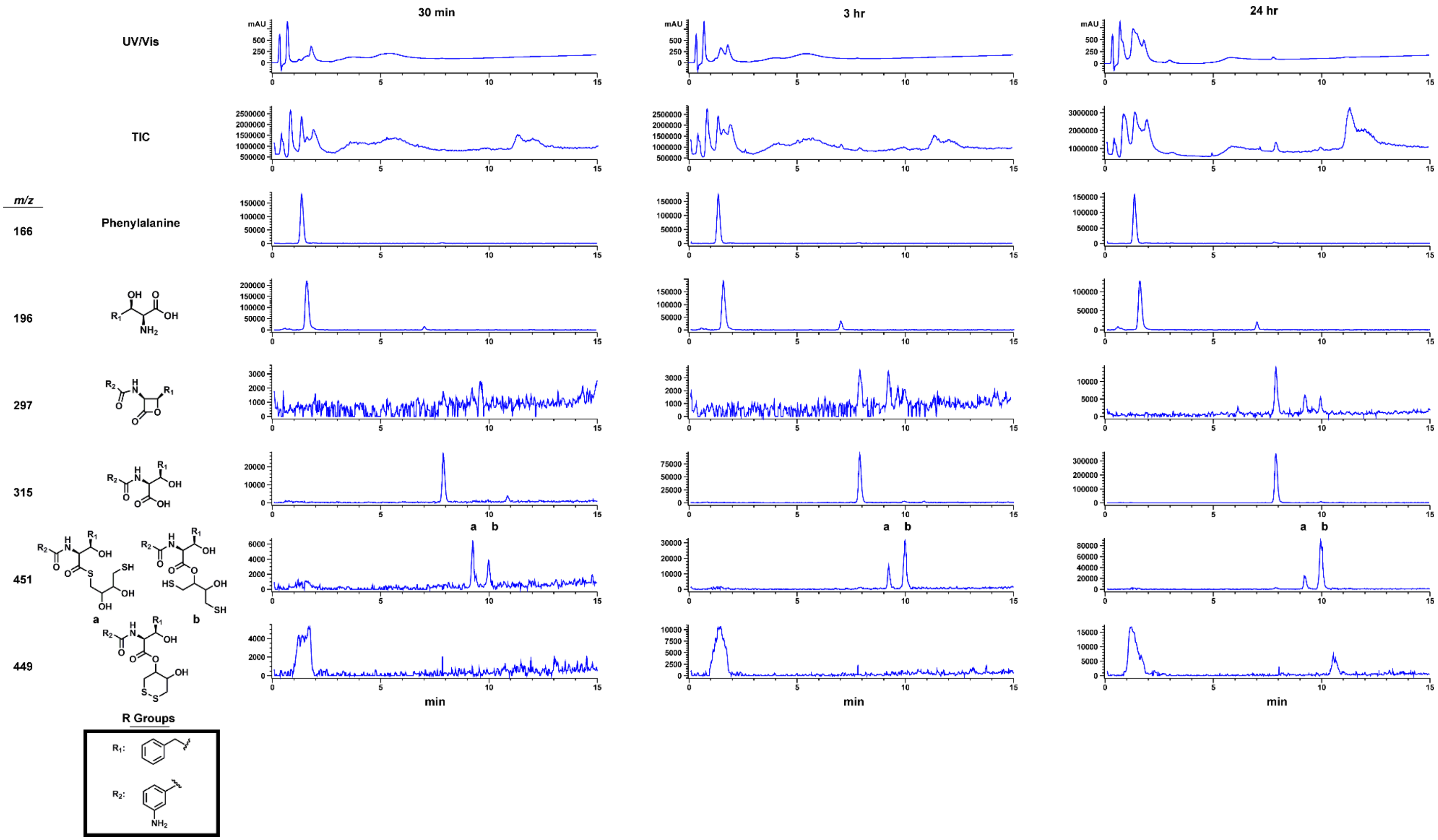
30 min
3 hr
24 hr
UV/Vis
TIC
m/z
166
Phenylalanine
196
297
315
451
449
a
b
R Groups
R_1:
R_2:
NH_2
min

ag

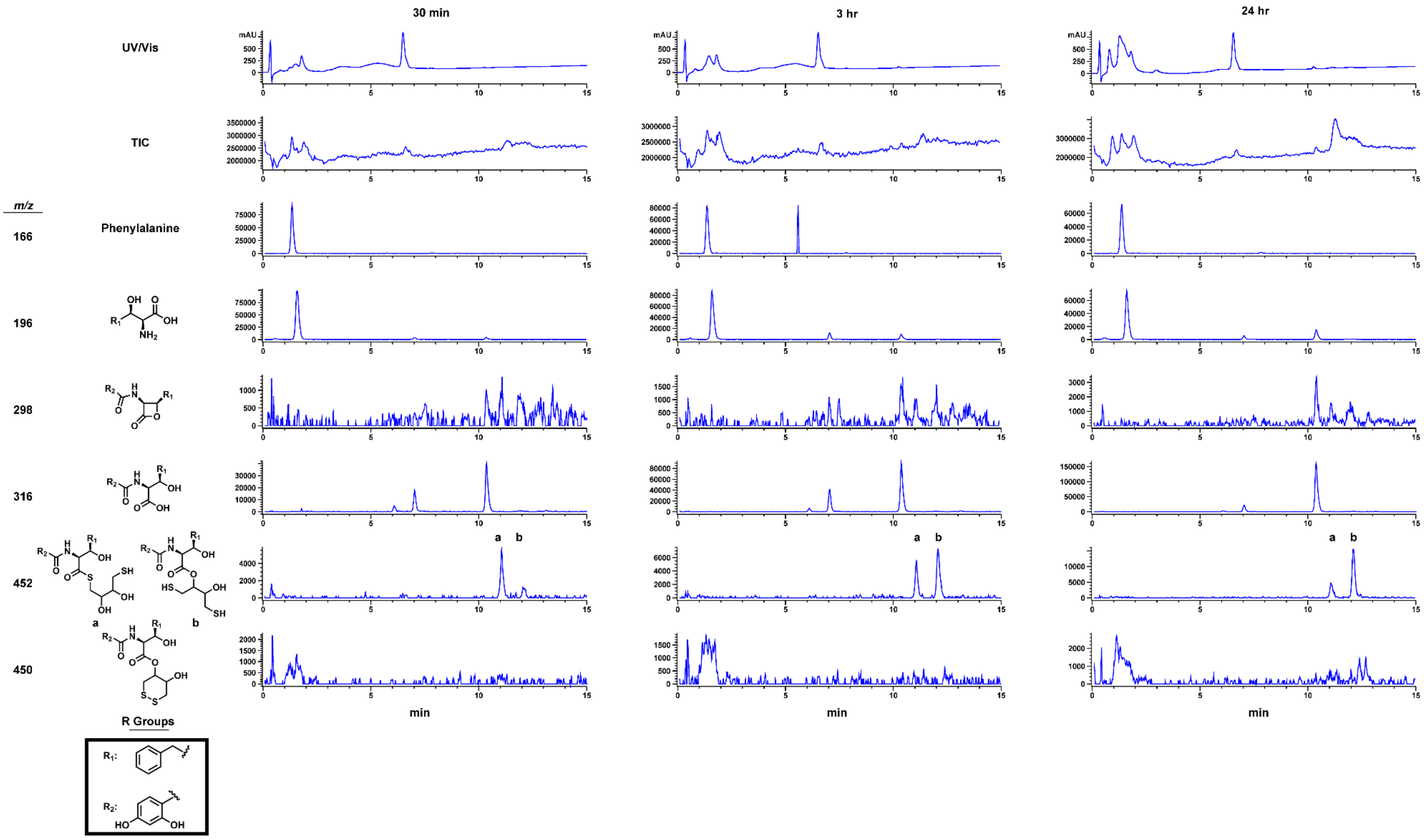
30 min
3 hr
24 hr
UV/Vis
TIC
m/z
166
Phenylalanine
196
298
316
452
450
a
b
min
R Groups
R1:
R2:

ah

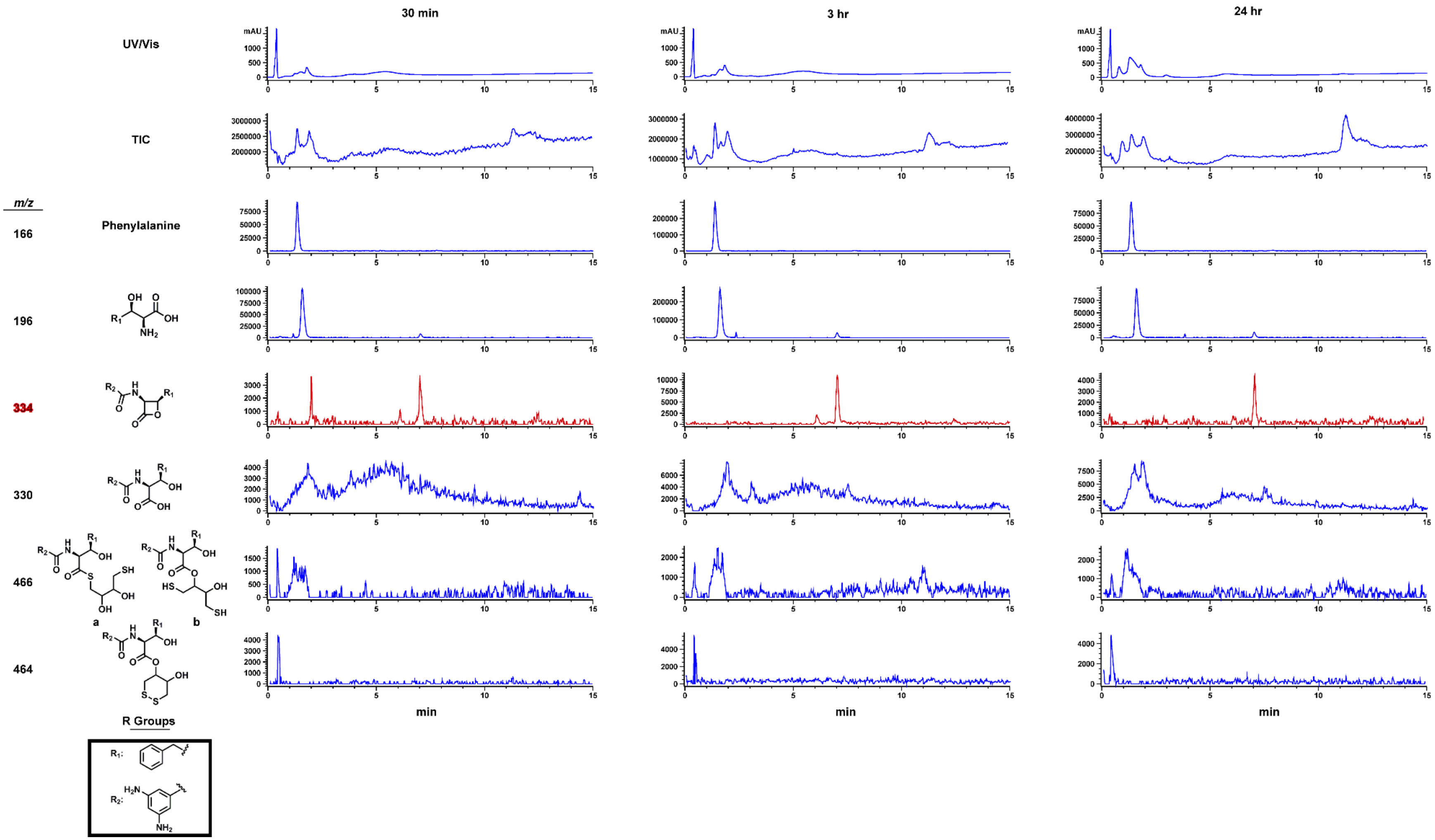

30 min
3 hr
24 hr
UV/Vis
TIC
m/z
166
Phenylalanine
196
334
330
466
a
b
464
R Groups
min

ai

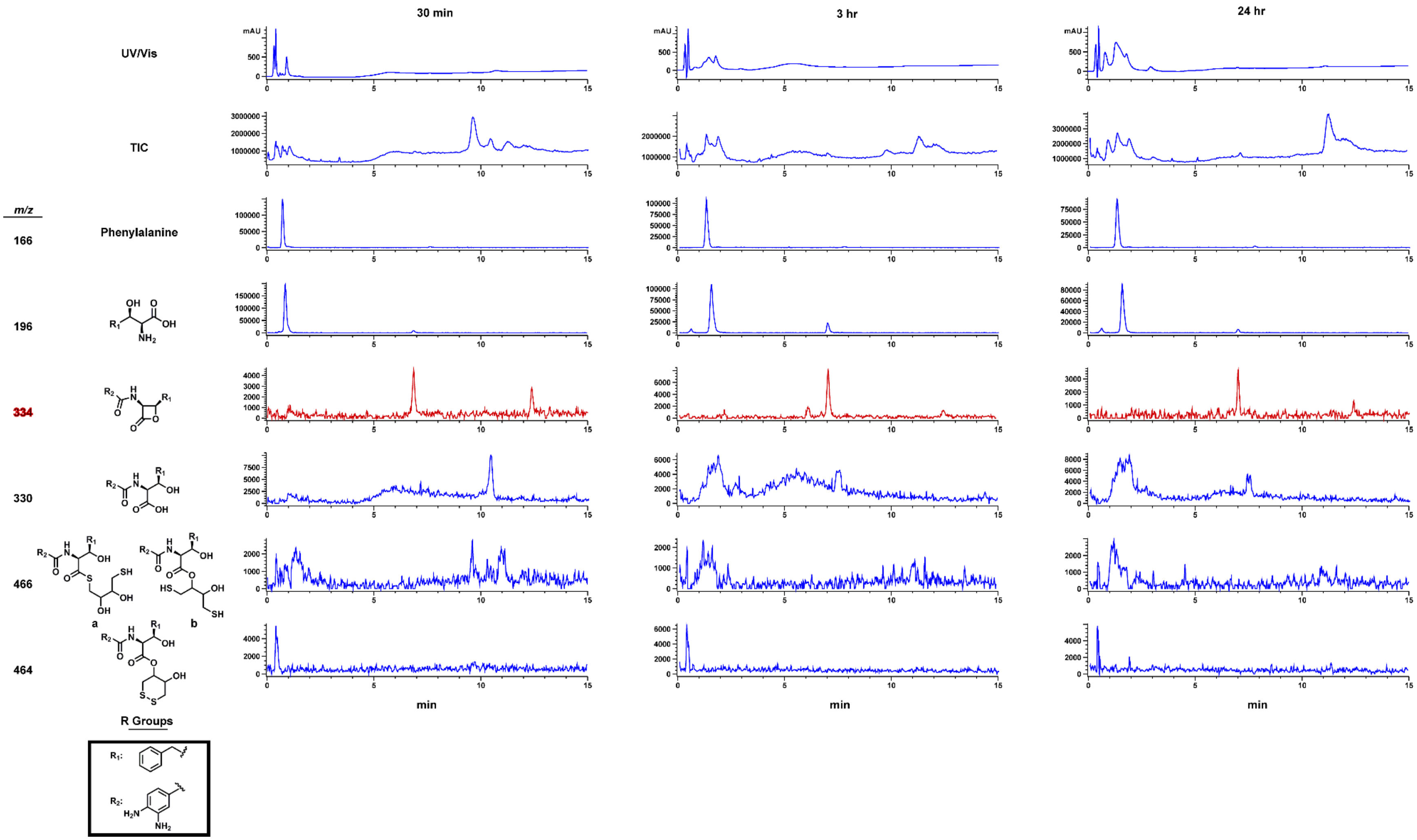
30 min
3 hr
24 hr
UV/Vis
TIC
m/z
166
Phenylalanine
196
334
330
466
464
a
b
R Groups
min

aj

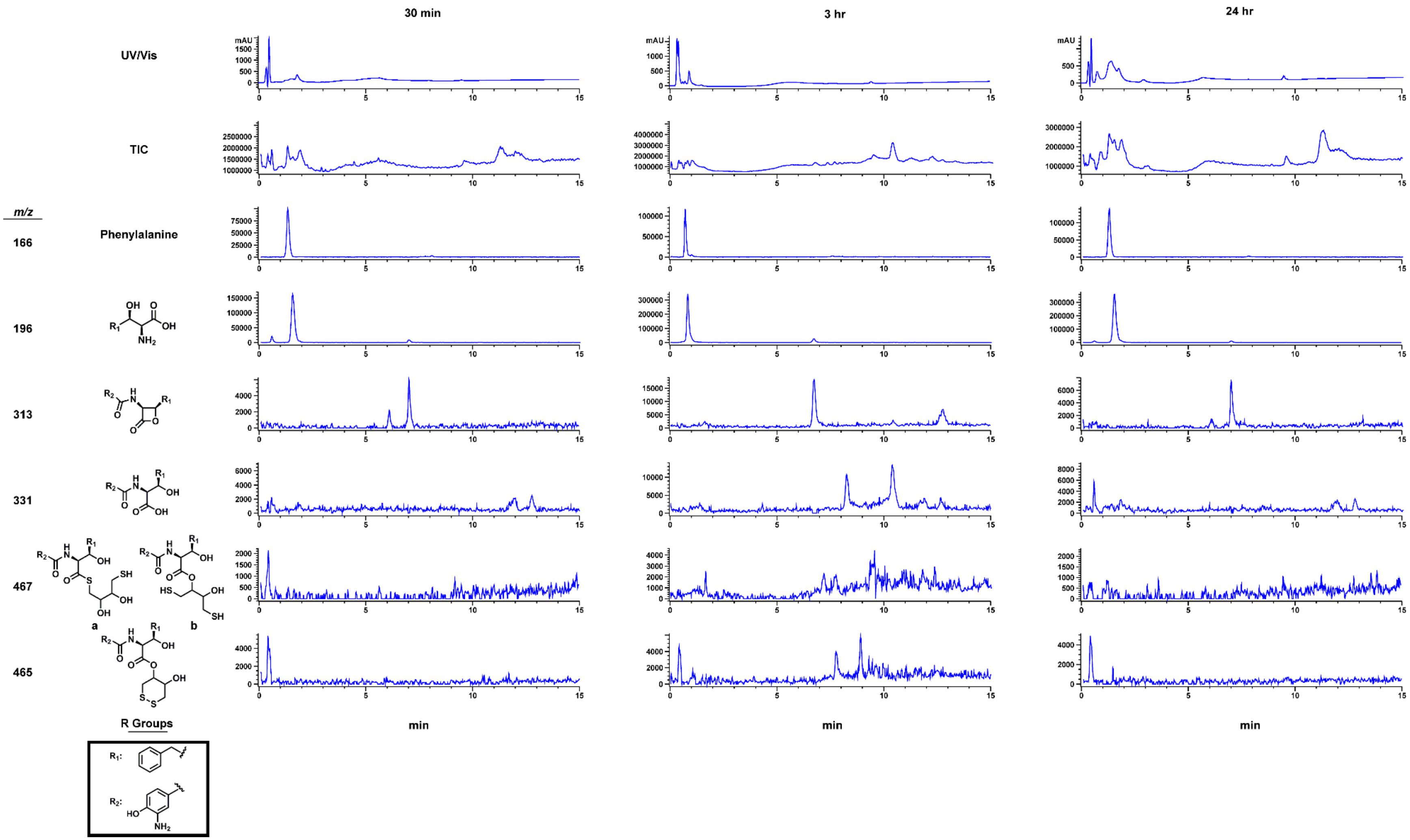
30 min
3 hr
24 hr
UV/Vis
TIC
m/z
166
Phenylalanine
196
313
331
467
a
b
465
R Groups
R1:
R2:
min
min
min

ak

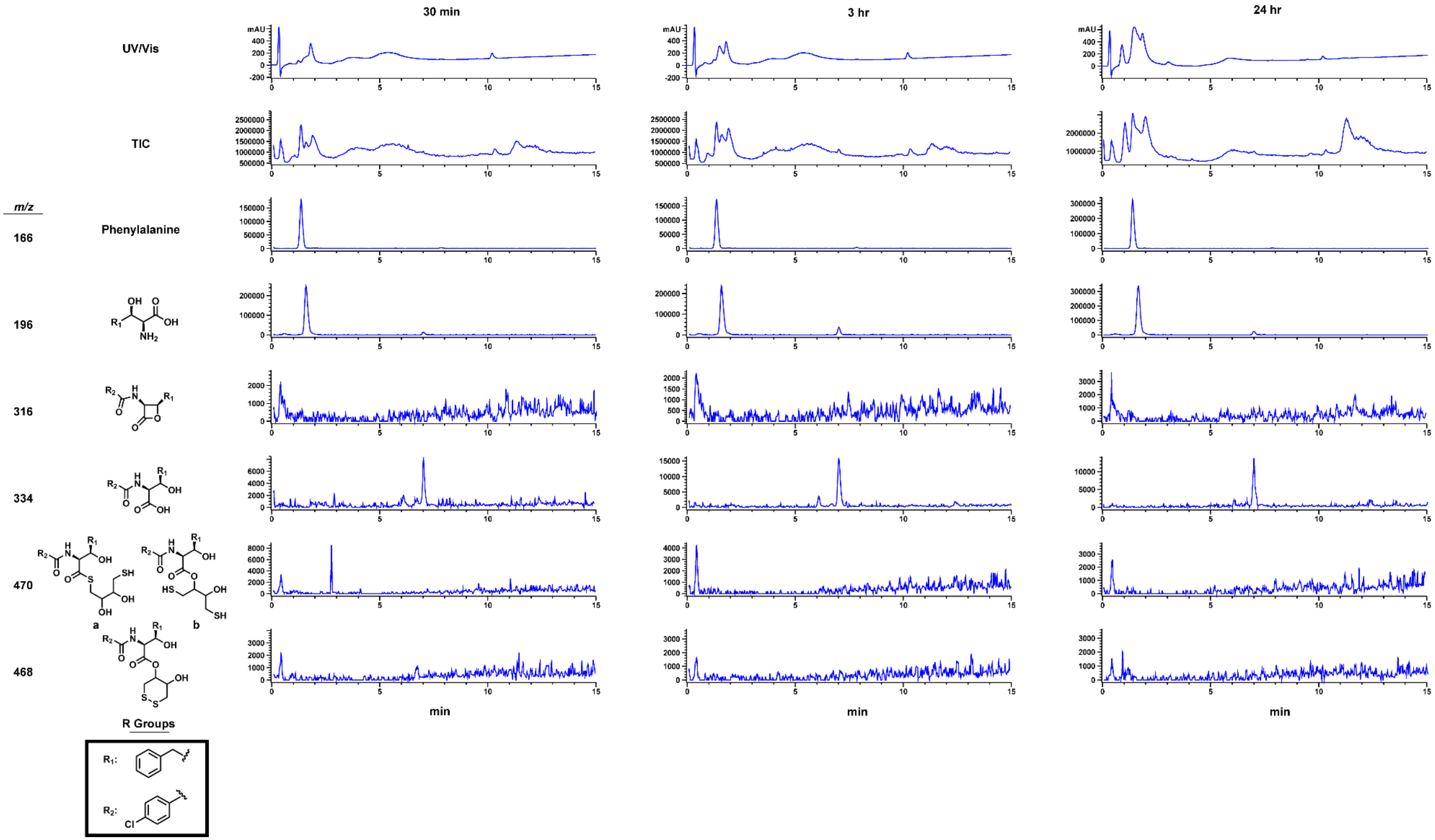
30 min
3 hr
24 hr
UV/Vis
TIC
m/z
166
Phenylalanine
196
316
334
470
a
b
468
R Groups
R1:
R2:
Cl
min
min
min

al

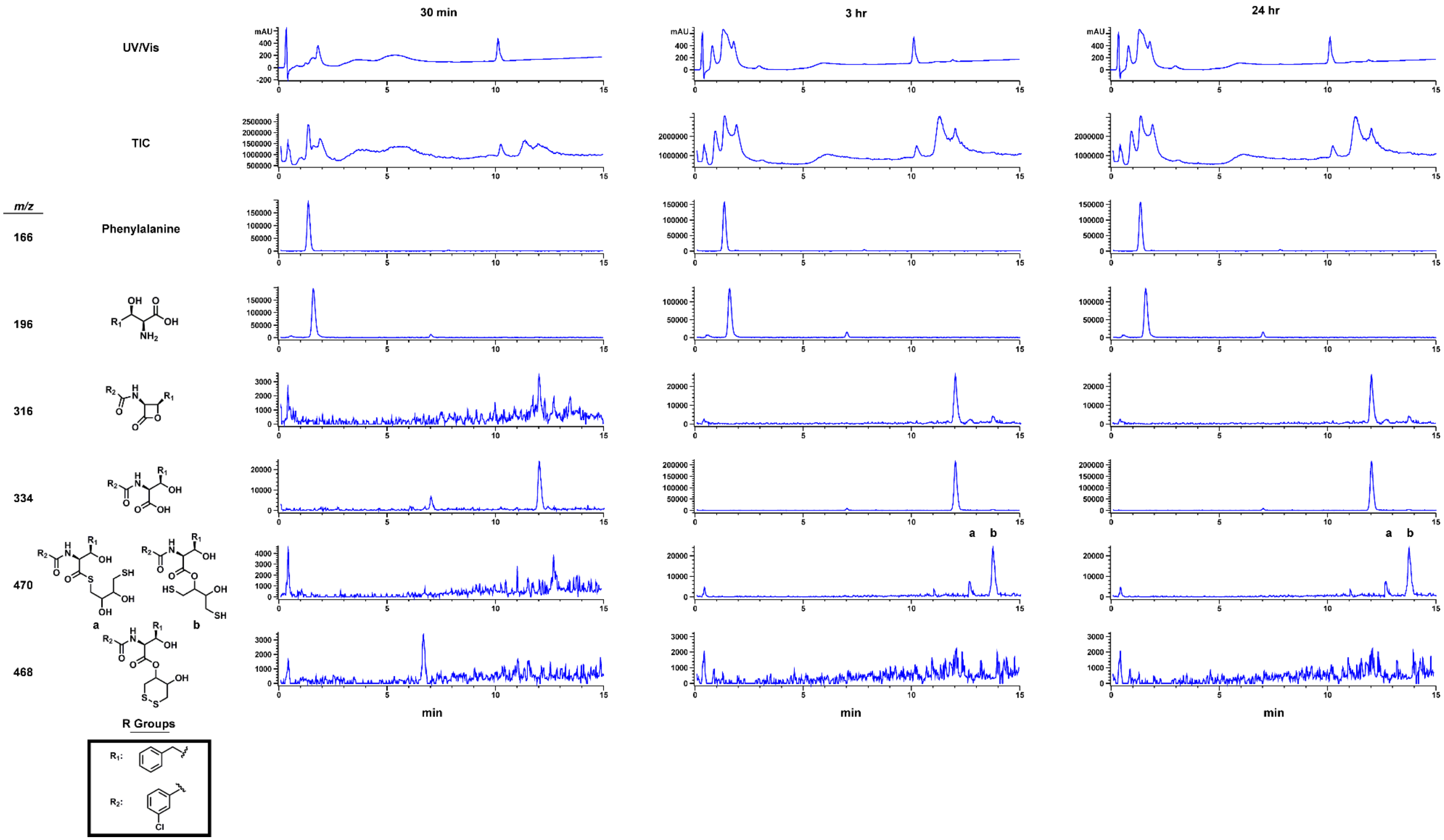
30 min
3 hr
24 hr
UV/Vis
TIC
m/z
166
Phenylalanine
196
316
334
470
468
a
b
min
R Groups

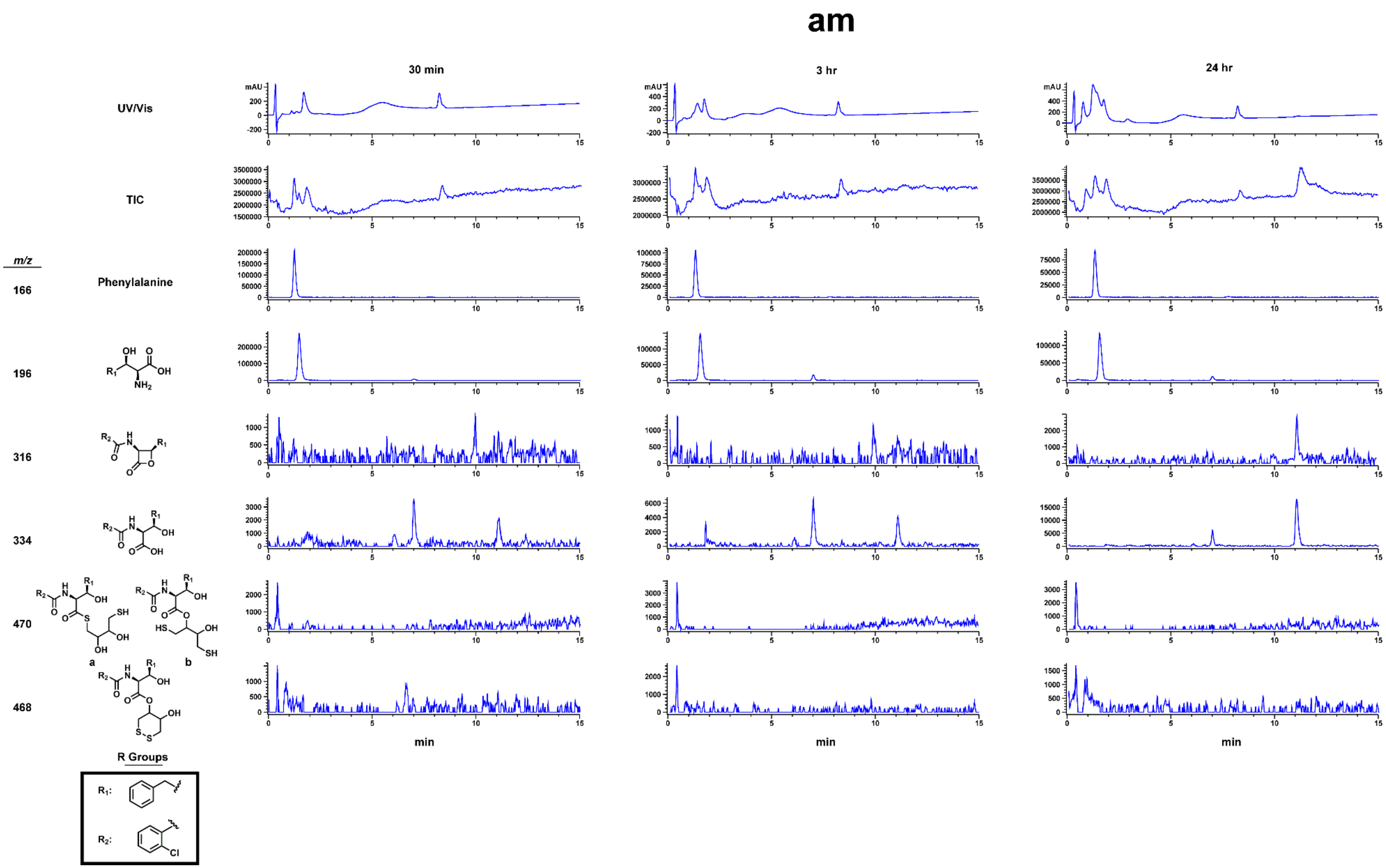

am
30 min
3 hr
24 hr
UV/Vis
TIC
m/z
166
Phenylalanine
196
316
334
470
468
a
b
R Groups
min

an

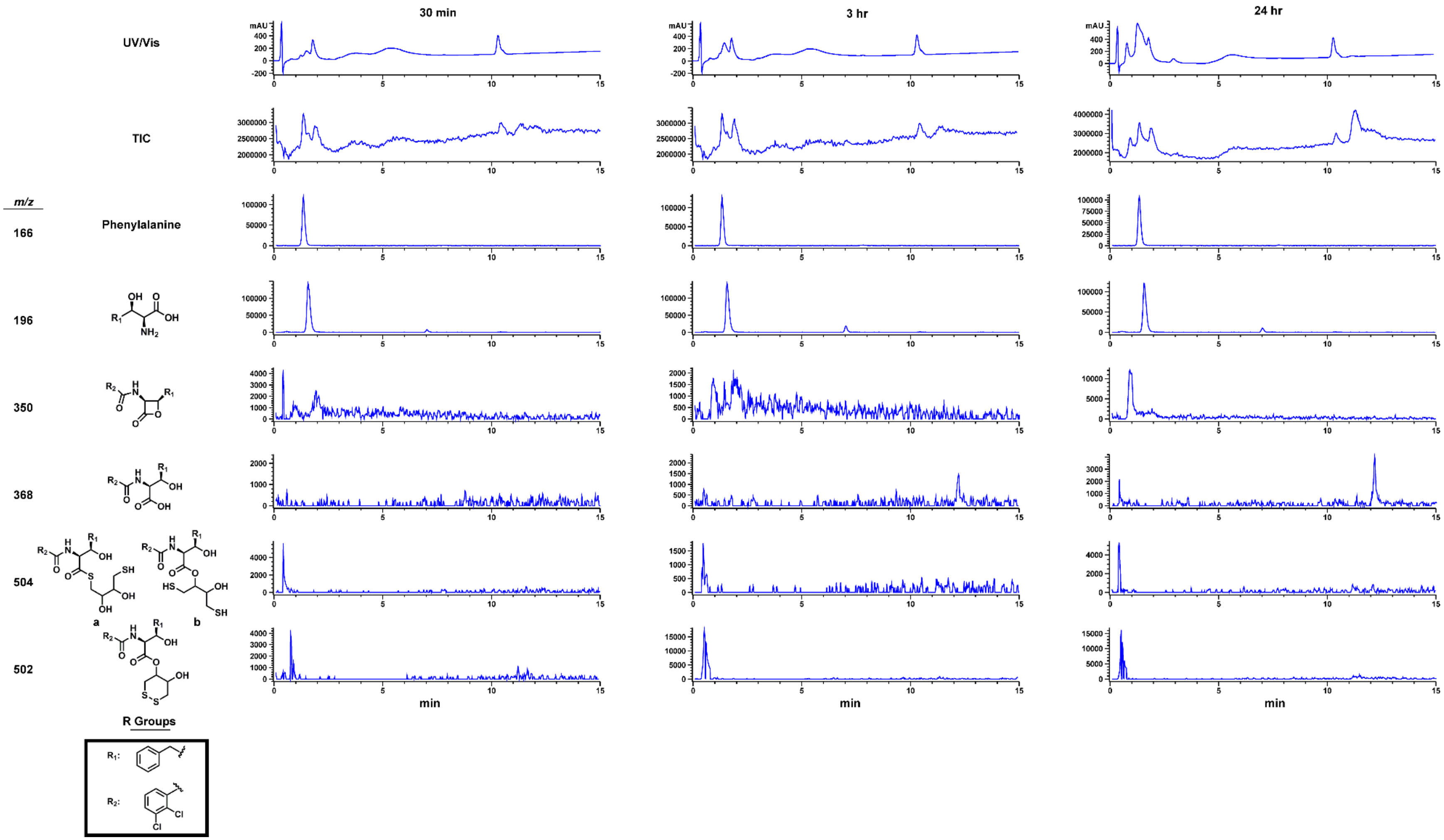

30 min
3 hr
24 hr
UV/Vis
TIC
m/z
166
Phenylalanine
196
350
368
504
a
b
502
R Groups
min

ao

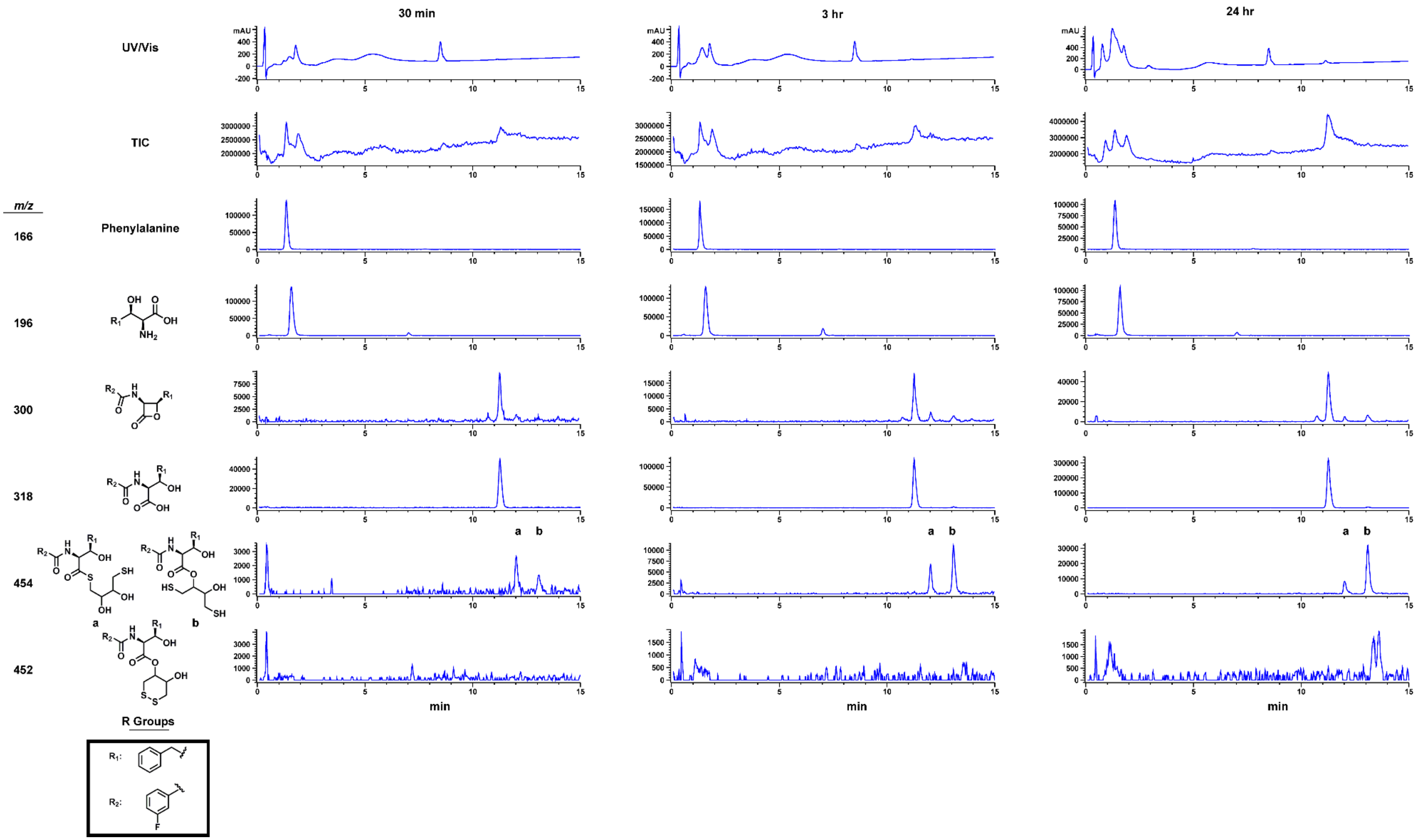
30 min
3 hr
24 hr
UV/Vis
TIC
m/z
166
Phenylalanine
196
300
318
454
a
b
452
R Groups
R1:
R2:
min

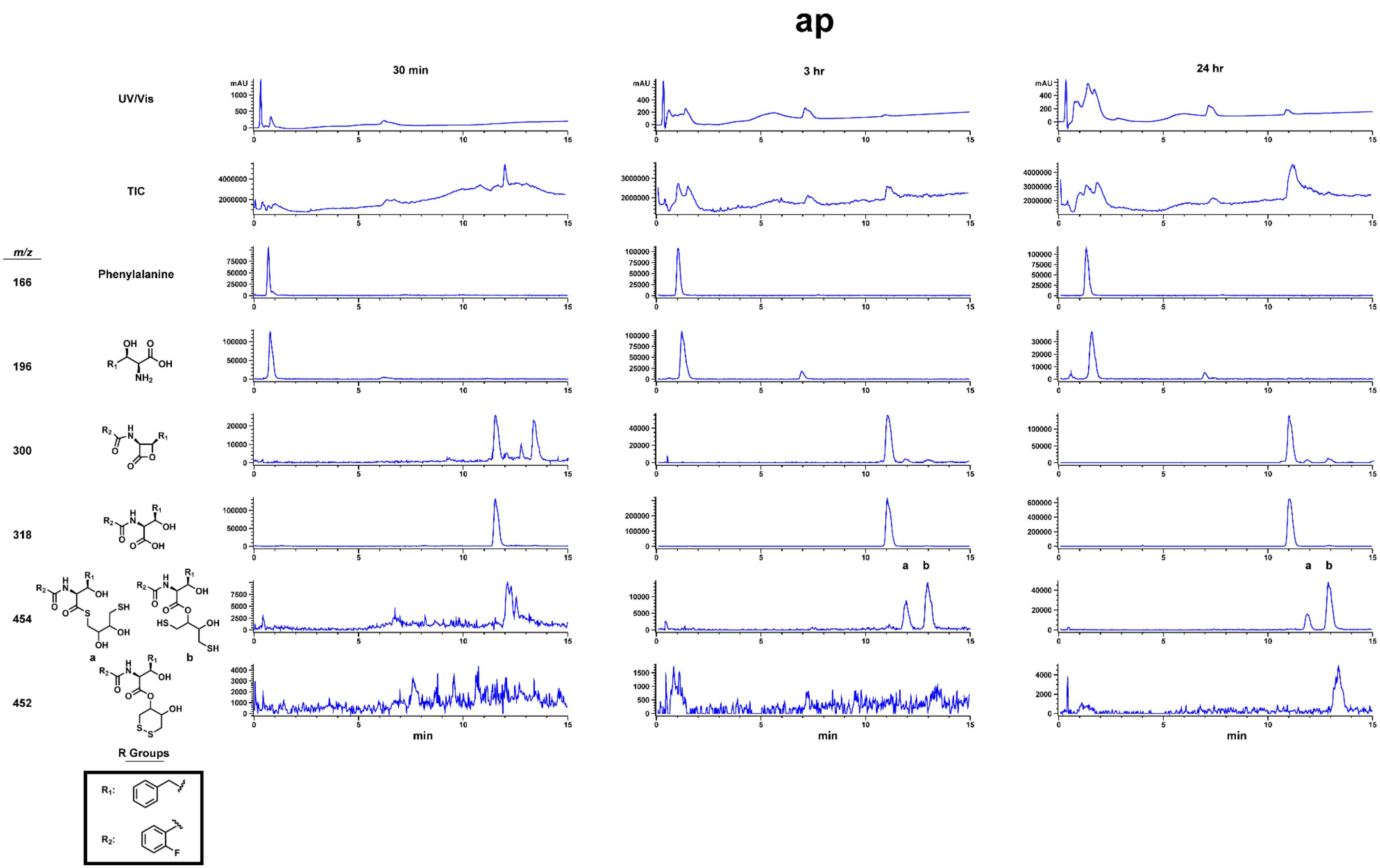

ap
30 min
3 hr
24 hr
UV/Vis
TIC
m/z
166
Phenylalanine
196
300
318
454
452
a
b
R Groups
min

aq

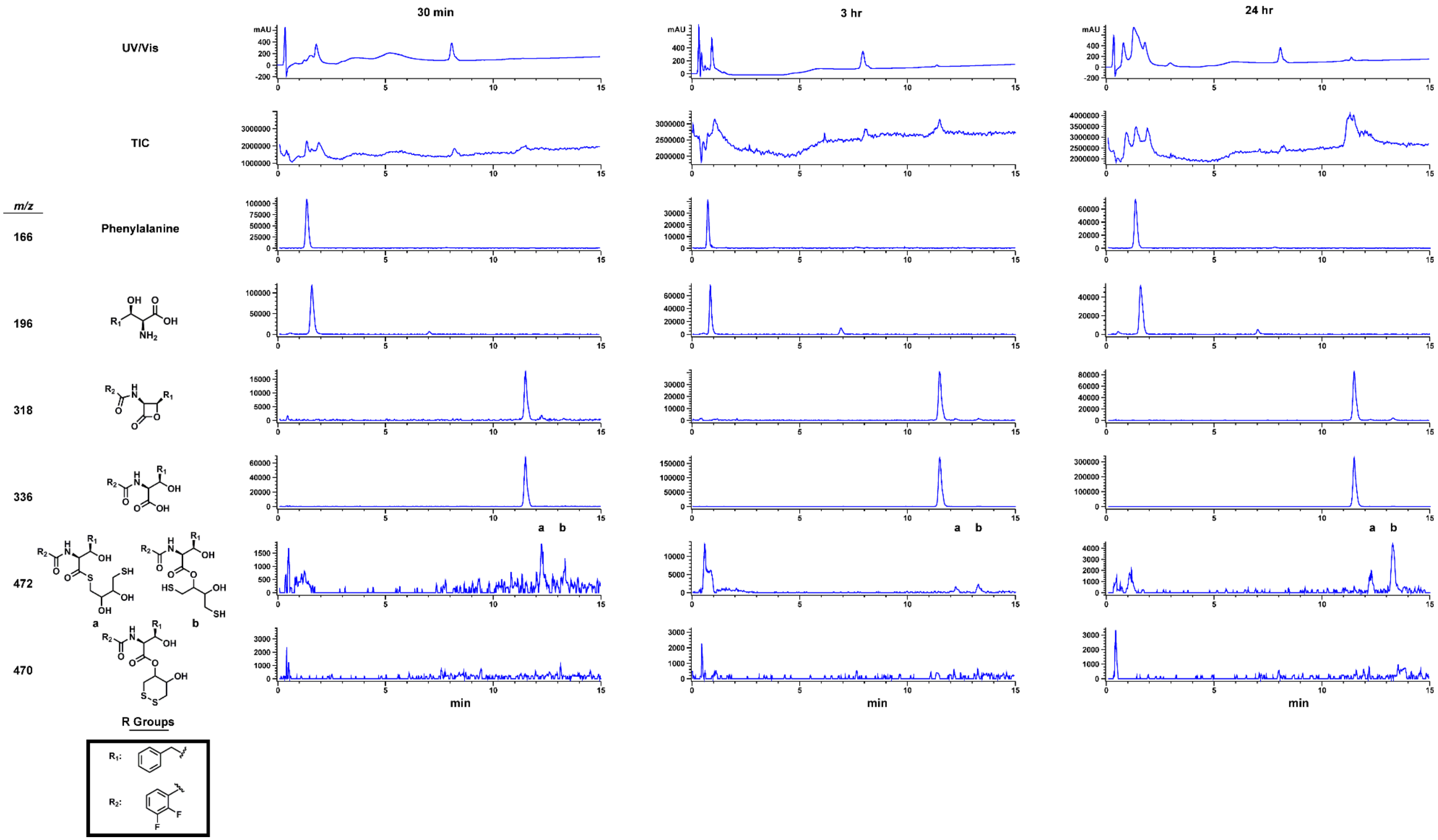
30 min
3 hr
24 hr
UV/Vis
TIC
m/z
166
Phenylalanine
196
318
336
472
470
a
b
min
R Groups
R1:
R2:

ar

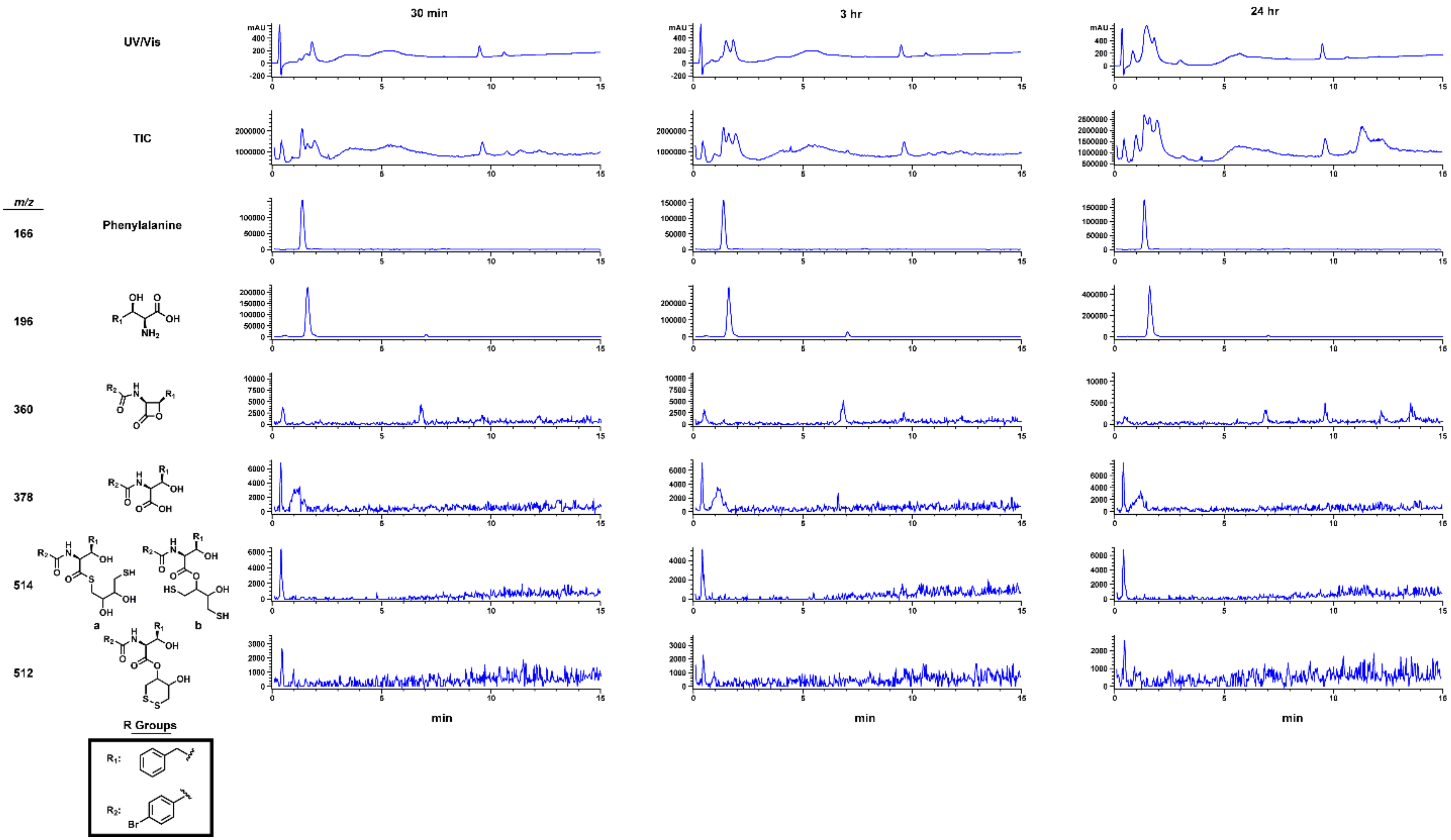
30 min
3 hr
24 hr
UV/Vis
TIC
m/z
166
Phenylalanine
196
360
378
514
a
b
512
R Groups
min
min
min

as

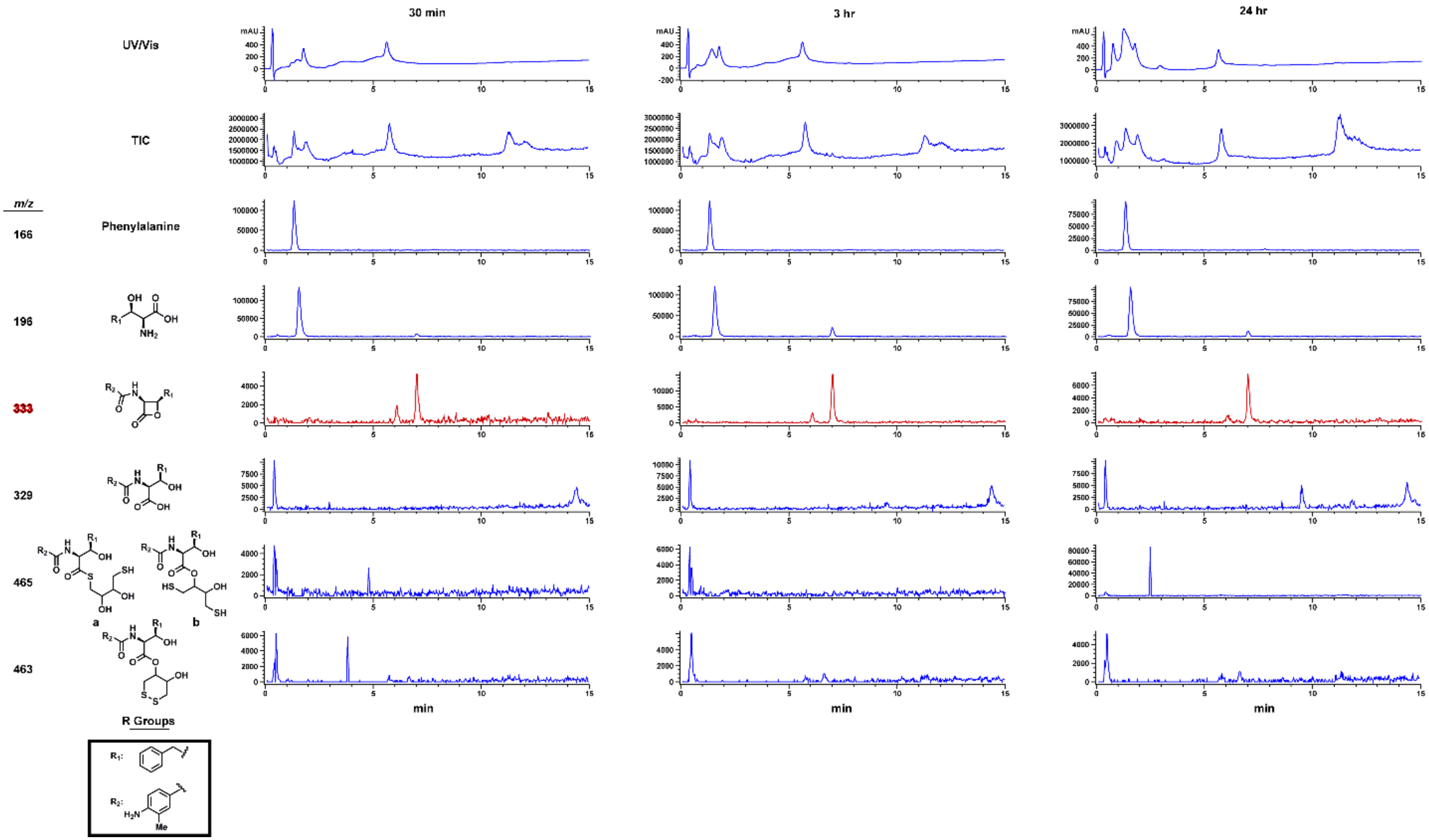

30 min
3 hr
24 hr
UV/Vis
TIC
m/z
166
Phenylalanine
196
333
329
465
463
a
b
R Groups
min

at

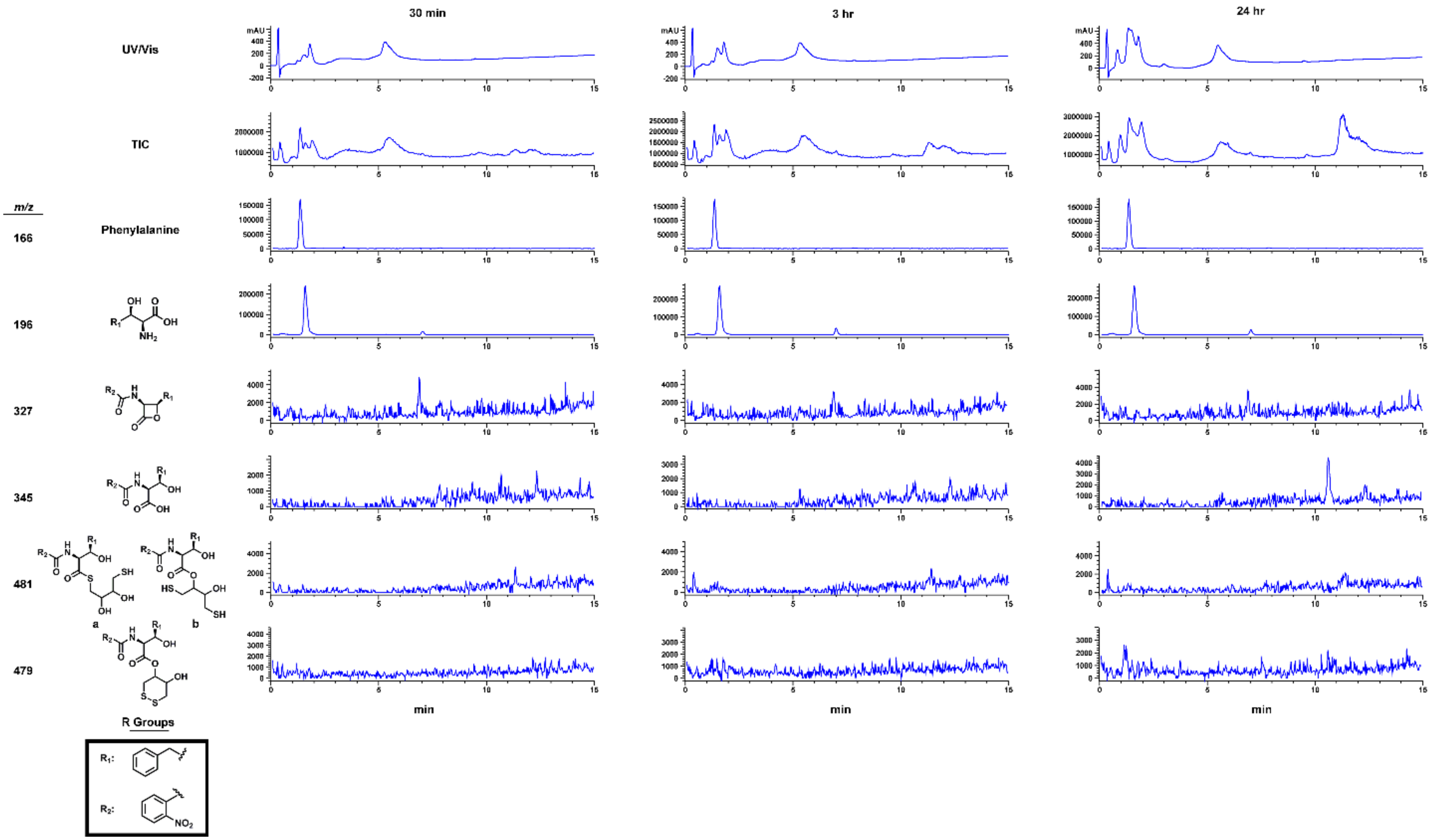

30 min
3 hr
24 hr
UV/Vis
TIC
m/z
166
Phenylalanine
196
327
345
481
479
a
b
R Groups
R1:
R2:
min

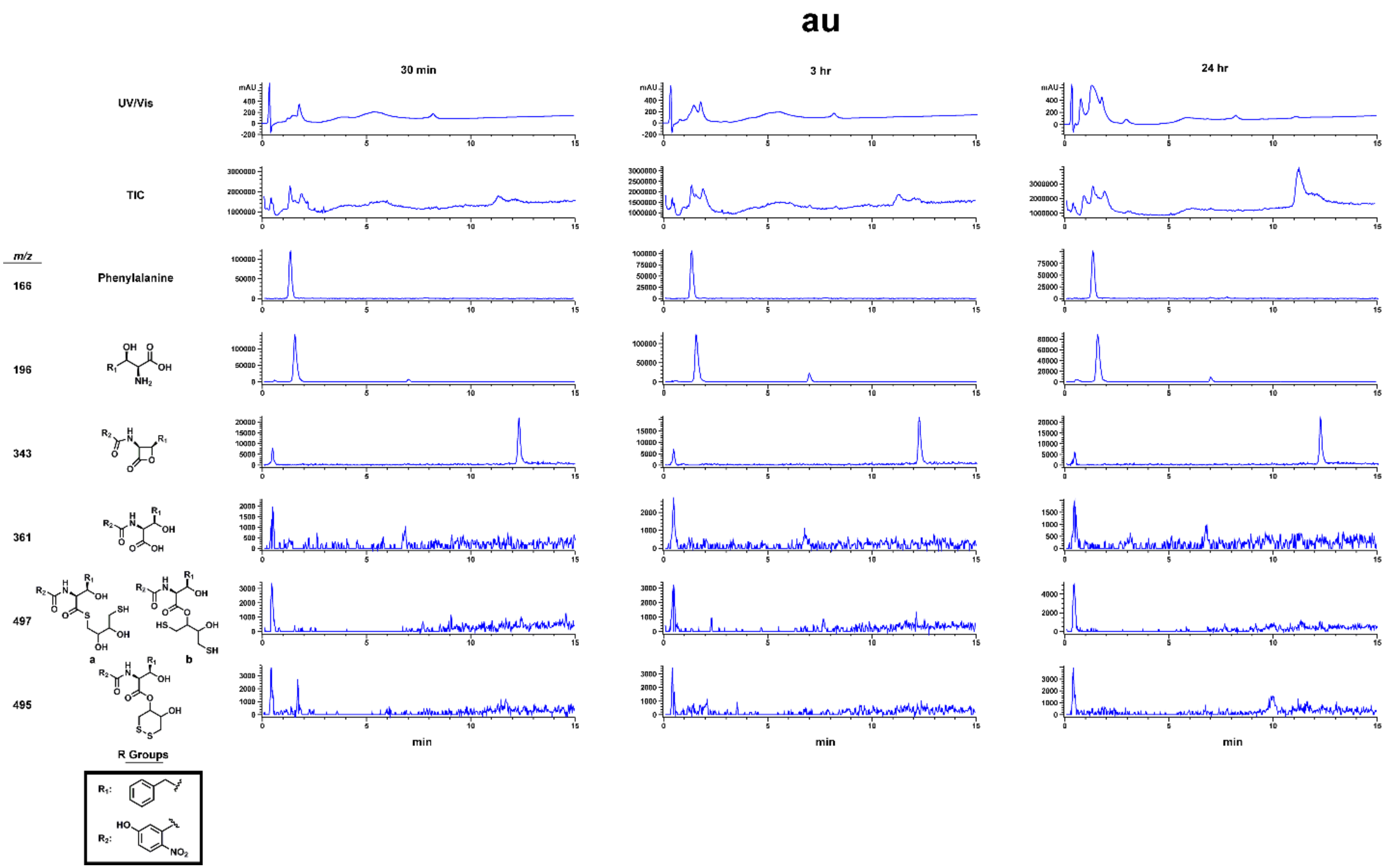

au
30 min
3 hr
24 hr
UV/Vis
TIC
m/z
166
Phenylalanine
196
343
361
497
a
b
495
R Groups
min

III. ObiF Mutant Assays

Scheme 3 and the associated LC-MS chromatograms provide the structures of substrates/products, ObiF1 variants, expected *m/z* values for $[M+H]^+$ ions, and raw LCMS data used for generation of ion count bar graphs (main text) for enzyme reactions with ObiF1 mutants. 2,3-DHB (**3a**) and phenylacetaldehyde (**1a**) were used as substrates for all reactions. All enzyme reactions were performed using the same conditions as described previously where ObiH, ObiD, and ObiF2 are wild-type enzymes from *Burkholderia diffusa*, ObiF1 is either wild-type (WT) or mutant variants (D1177A, R1236A, D1254A, G1173A/D1254A, R1190A, H1284A, D1254A/H1284A, G1173L, A841E, C1146S, C1146A, del1304, rbs1303/4), and MLP is the purified ObiF1 MLP domain added exogenously to the reaction (Scheme 3). Glutathione (GSH) was used as the thiol trapping agent for all reactions as described in the main text. All LCMS chromatograms are from low-resolution LCMS analysis of aliquots from the enzyme reactions at 30 minute, 3 hour, and 24 hour time points including the chromatograms for optical absorbance at 220 nm (UV/Vis), total ion counts (TIC), and extracted ion counts (EIC) for the phenylalanine internal standard and compound structures indicated on to the left of each EIC chromatogram. LC-MS samples were prepared by removing a 100 µL aliquot of the assay at 30 minutes, 3 hours, and 24 hours, quenched by acidification to pH 2 with 1 M HCl, addition of phenylalanine at 100 µM as internal standard, and immediately frozen at -80° C. At the time of LC-MS analysis, the sample is thawed, centrifuged at 16,000 rpm for 1 minute to pellet solids, and analyzed by LCMS (instrument: Agilent 6130 quadrupole with G1313 autosampler, G1315 diode array detector, 122 series solvent module; column: Phenomenex Gemini C18, 50 x 2 mm, 5 µm plus guard column; solvents: 0.1% formic acid in (A) water and (B) acetonitrile; method: 5% B to 100% B over 20 min; software: G2710 ChemStation). *NOTE: All enzyme reactions for this section were performed in at least two independent experiments. The nomenclature for LCMS chromatograms for both experimental trials are provided indicated by a “1” or a “2” at the end of the label above each set of chromatograms. For assays using ObiF1 mutants C1146A, del1304, and rbs1303/4 and where exogenous ObiF1-MLP was added at variable concentrations the phenylacetaldehyde was prepared fresh via oxidation of phenethylalcohol using Dess-Martin periodinane followed by purification via silica gel chromatography and quantification of stock solutions via ^{1}H-NMR.

Scheme 3: ObiF mutant assays for coupled ObiHDF1F2 enzyme reactions with 2,3-DHB (3a) and phenylacetaldehyde (1a) as substrates.

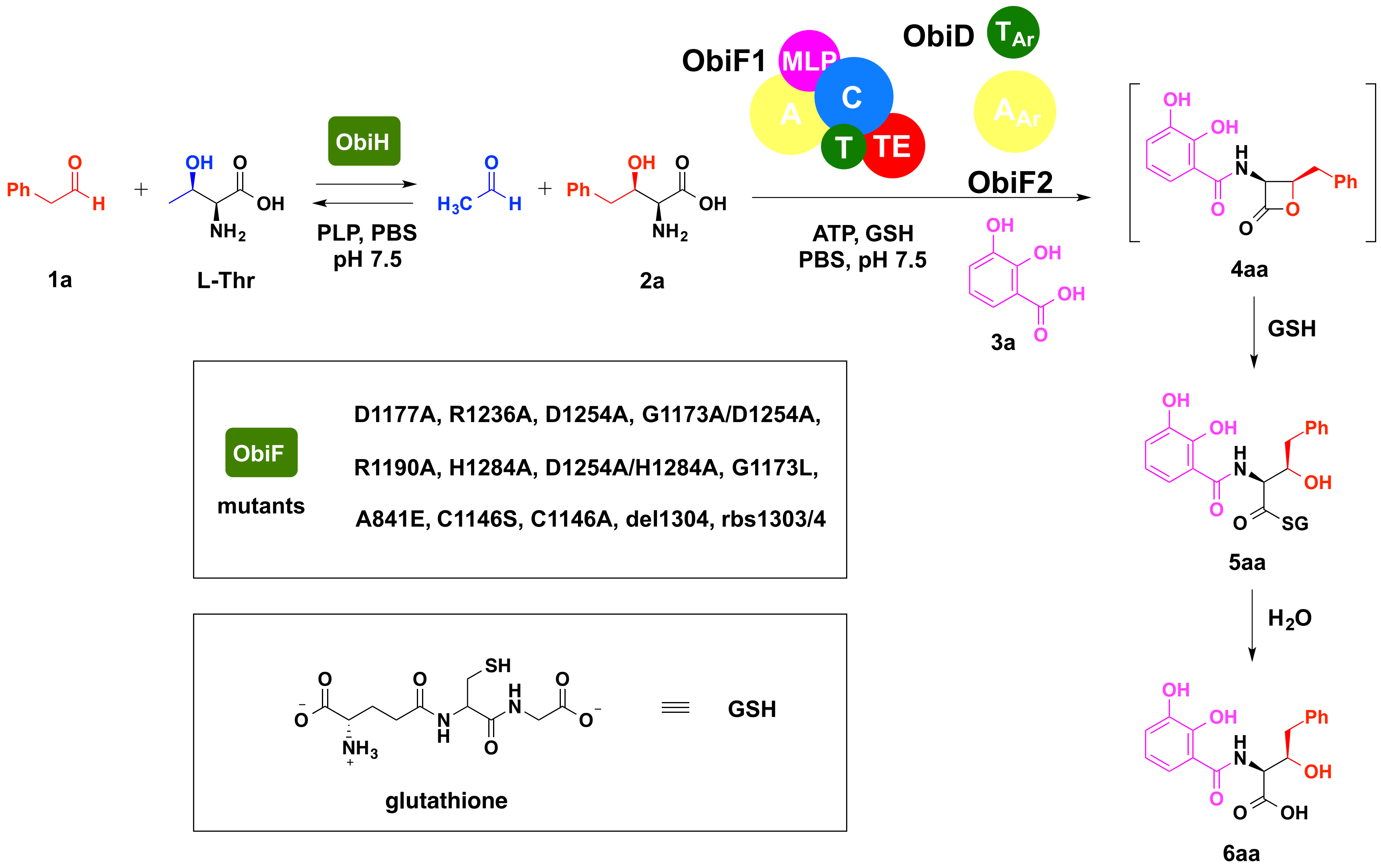

LCMS chromatograms of ObiF mutant assays for coupled ObiHDF1F2 enzyme reactions with 2,3-DHB (3a) and phenylacetaldehyde (1a) as substrates.

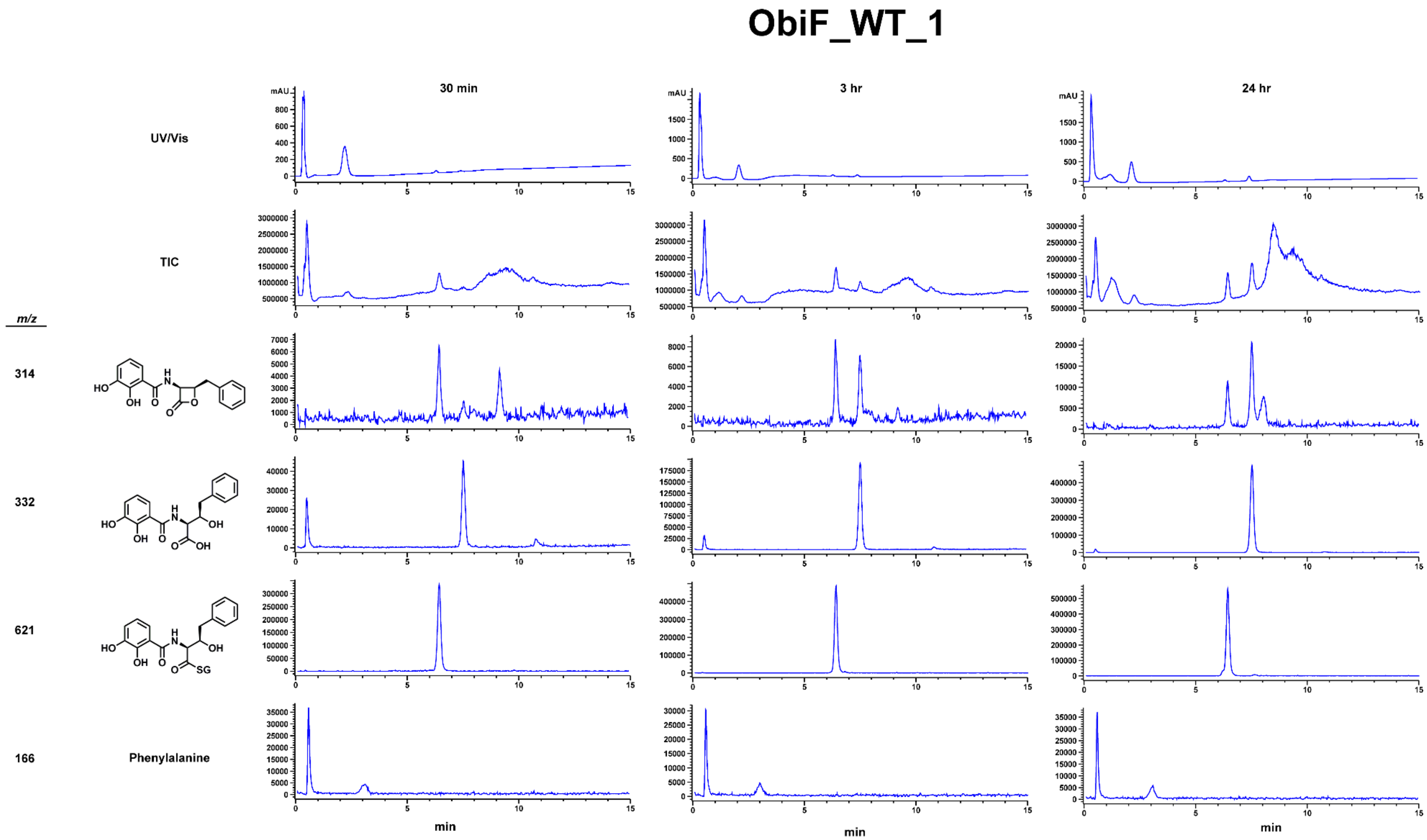

ObiF_WT_2

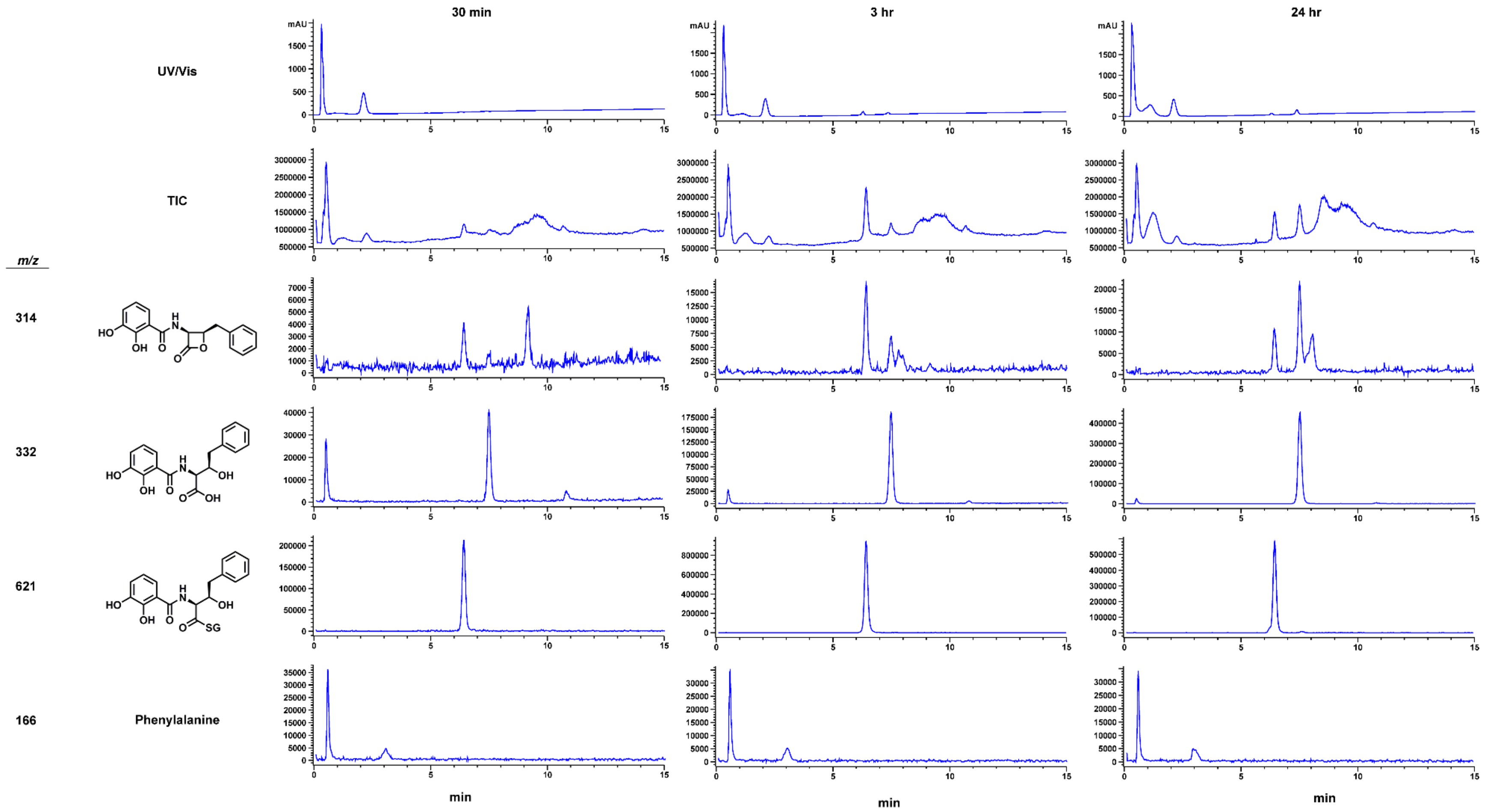

30 min
3 hr
24 hr
UV/Vis
TIC
m/z
314
332
621
166
Phenylalanine
mAU
min

ObiF_D1177A_1

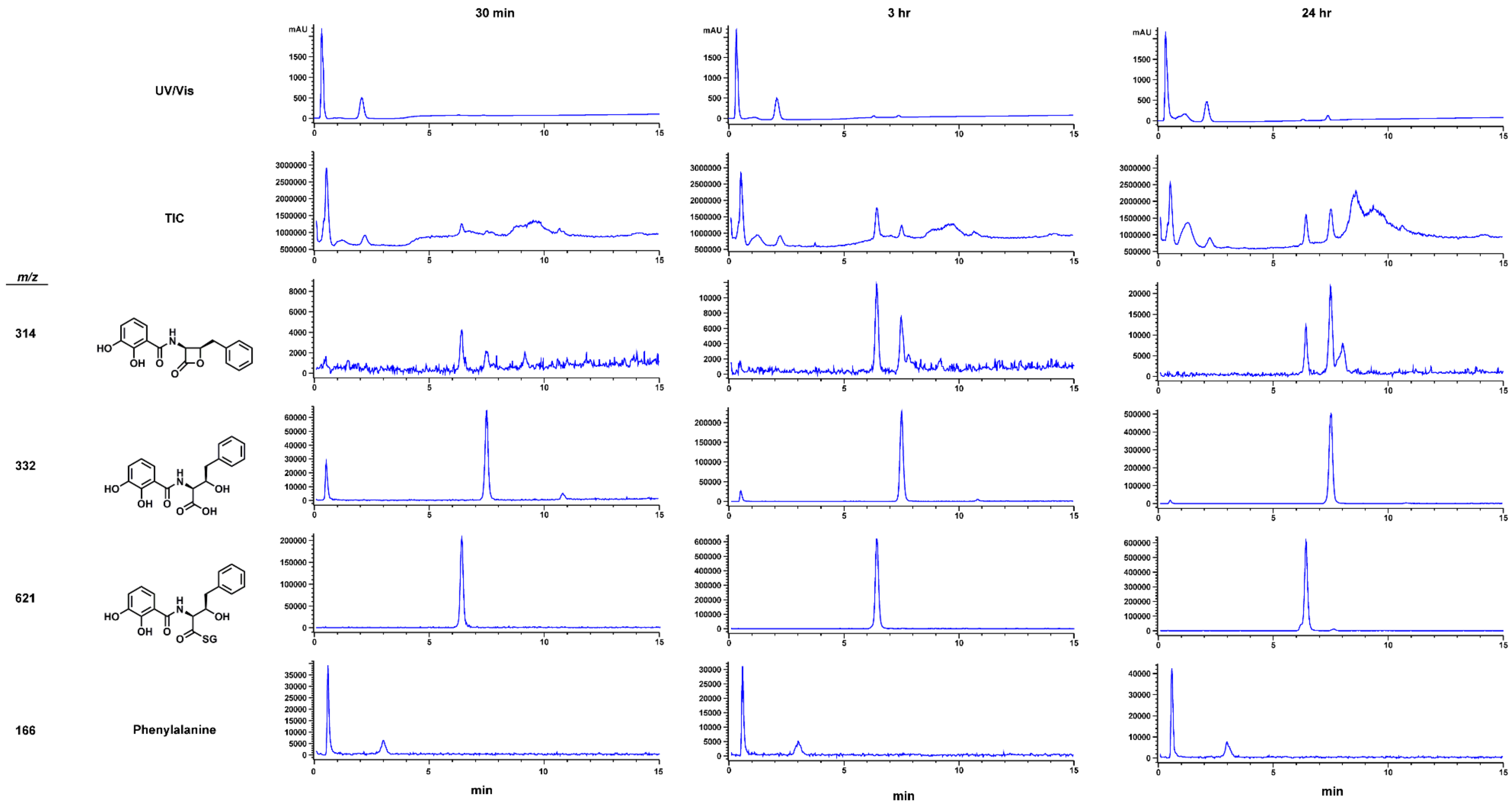

ObiF_D1177A_2

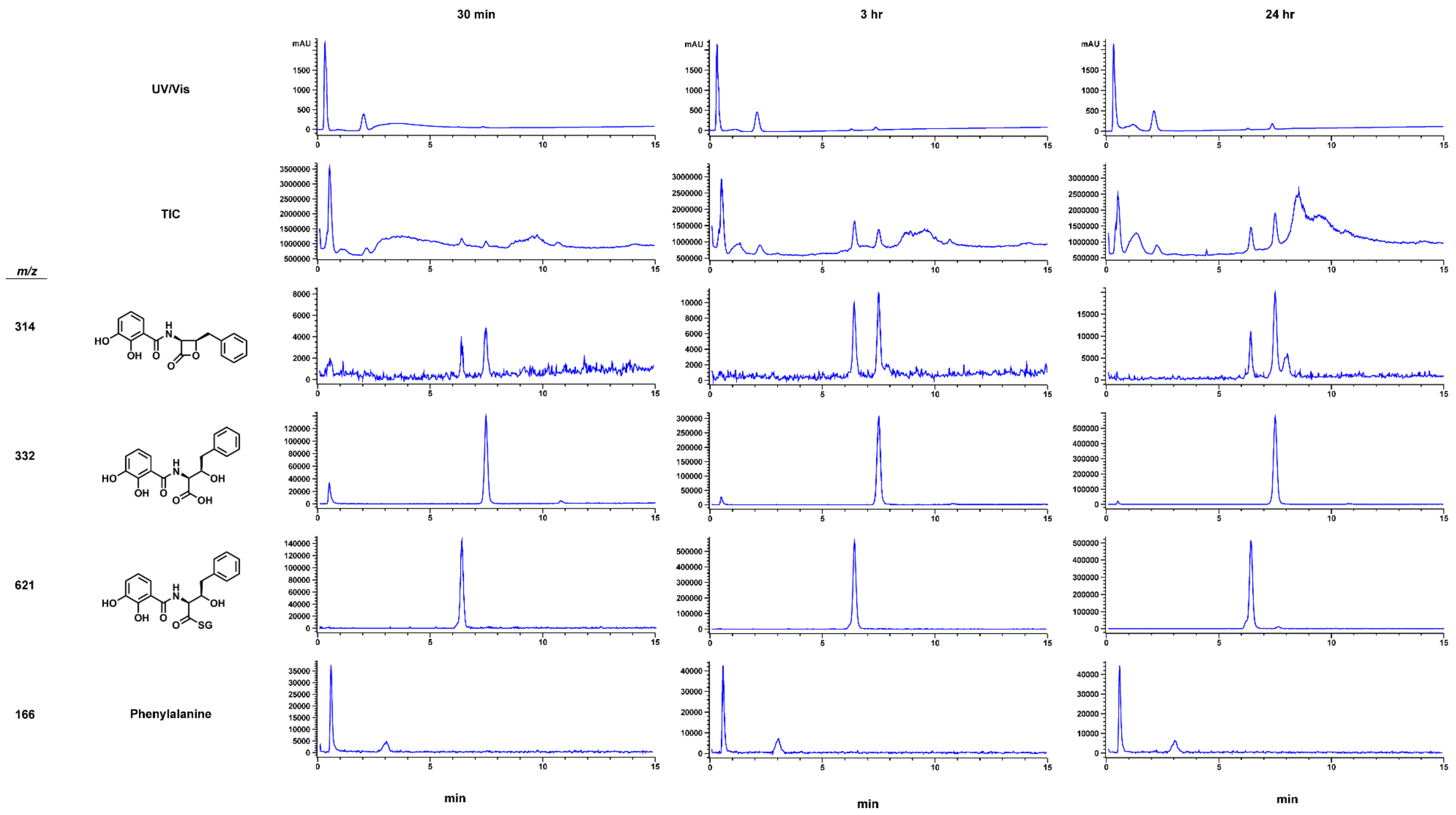

ObiF_R1263A_1

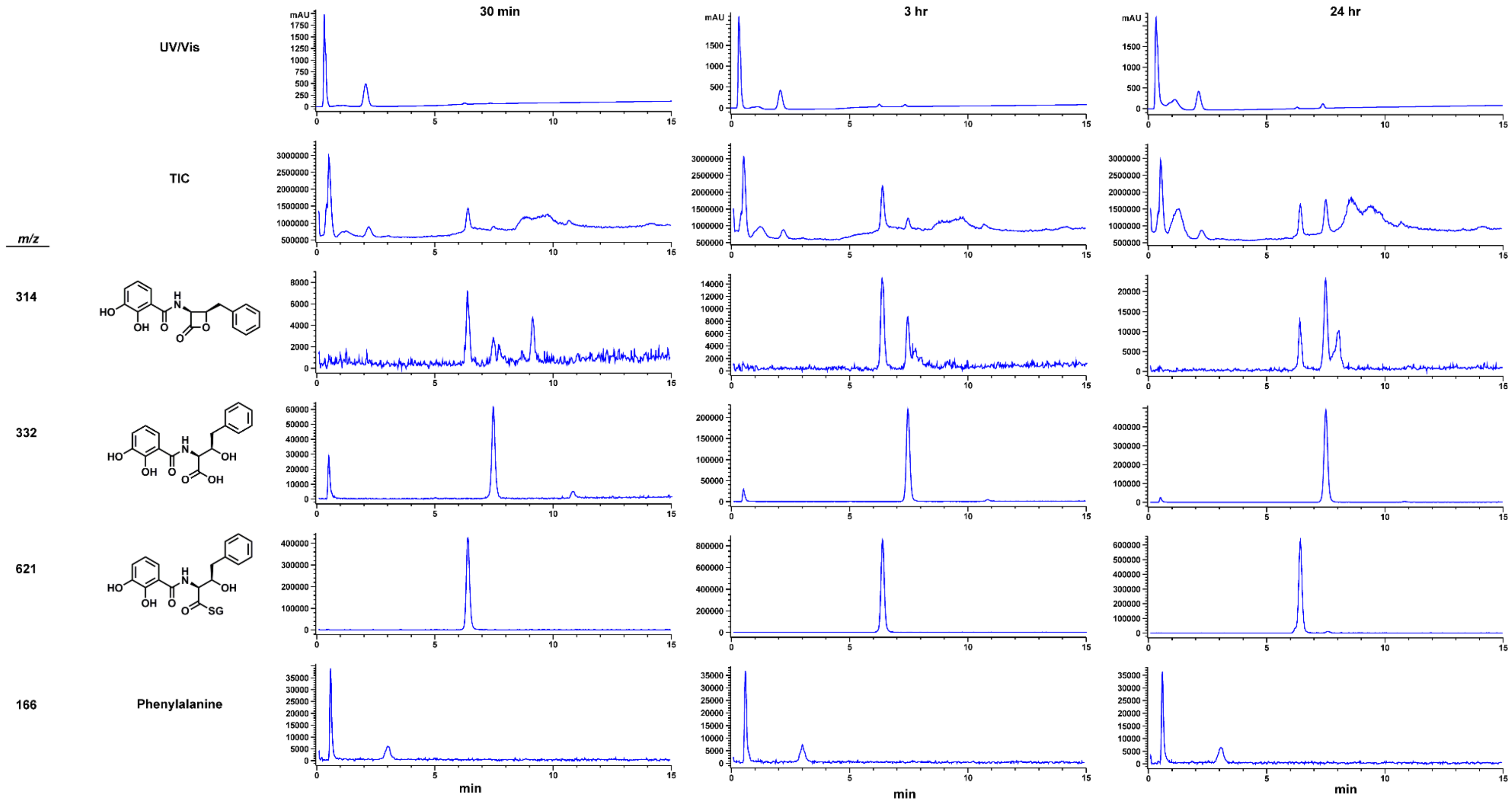

ObiF_R1263A_2

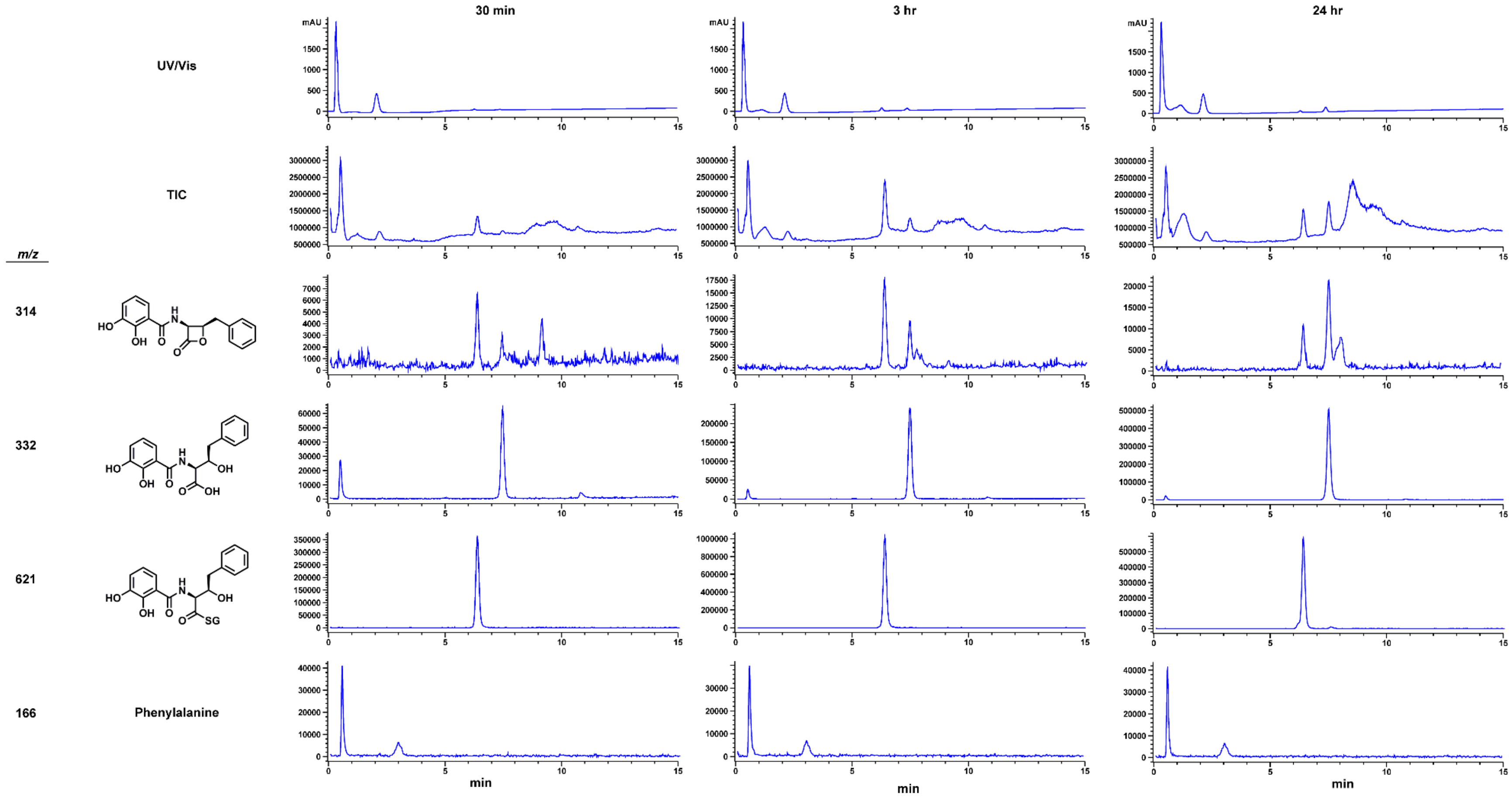

ObiF_D1254A_1

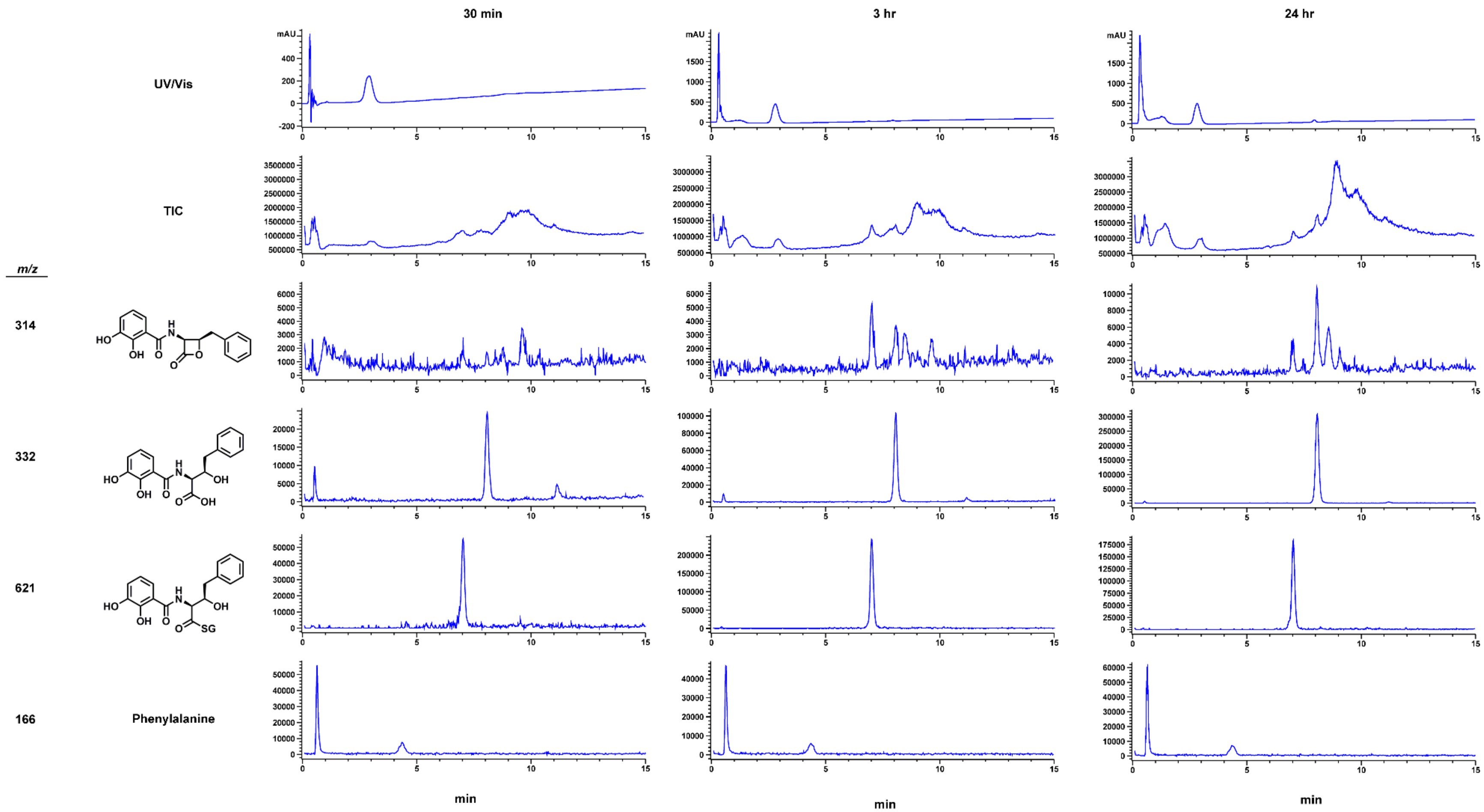

ObiF_D1254A_2

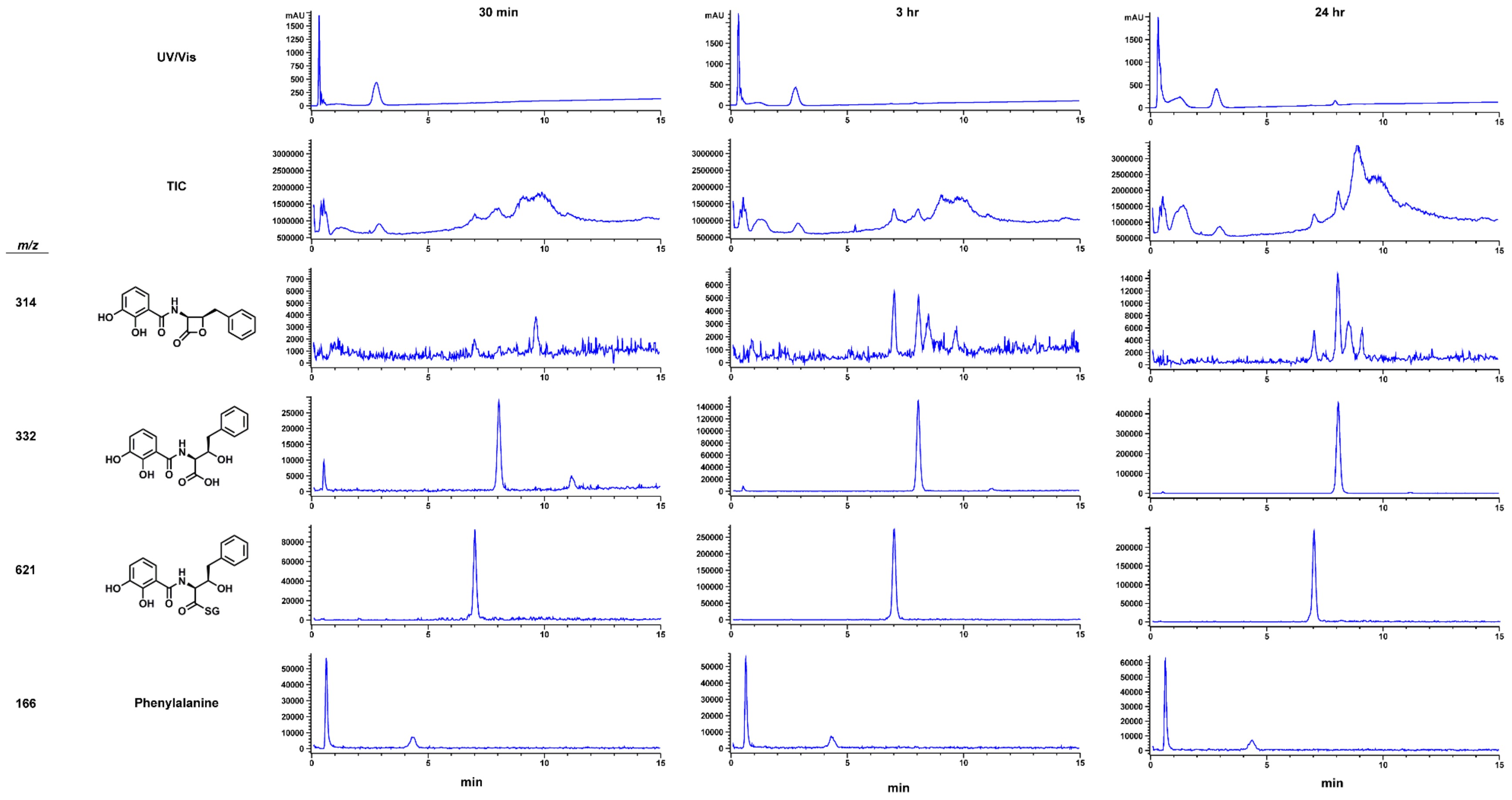

ObiF_G1173A_D1254A_1

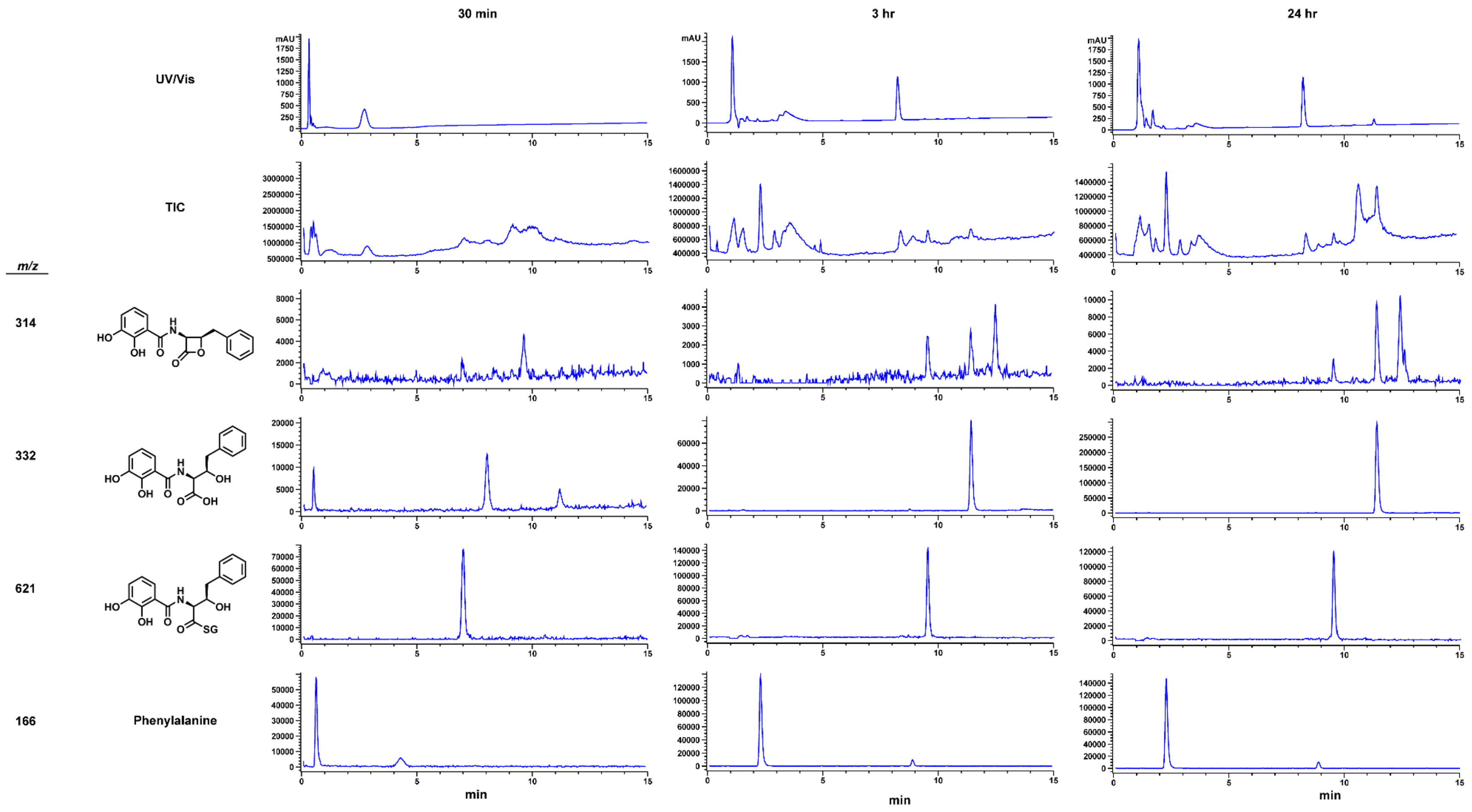

ObiF_G1173A_D1254A_2

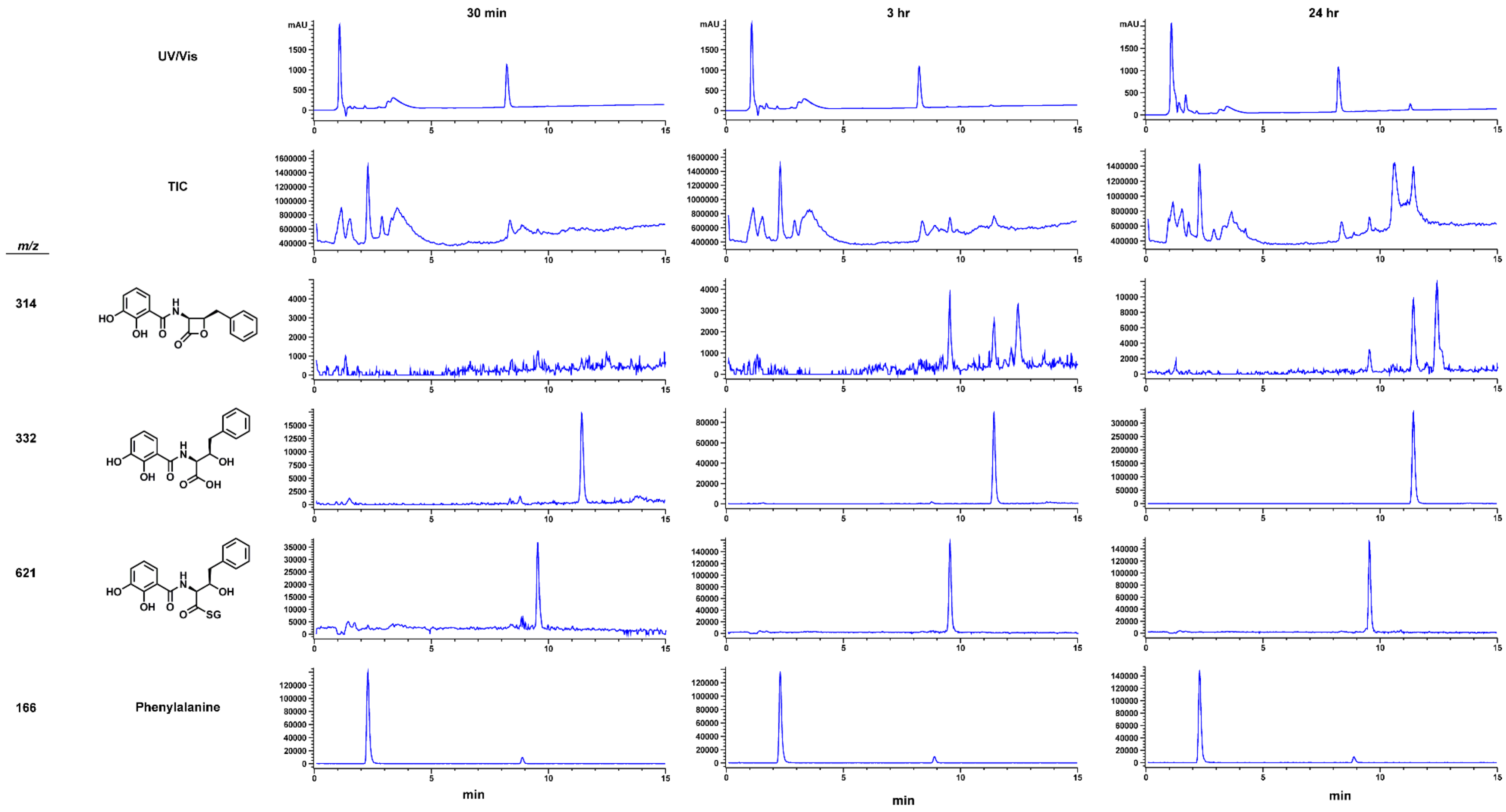

ObiF_R1190A_1

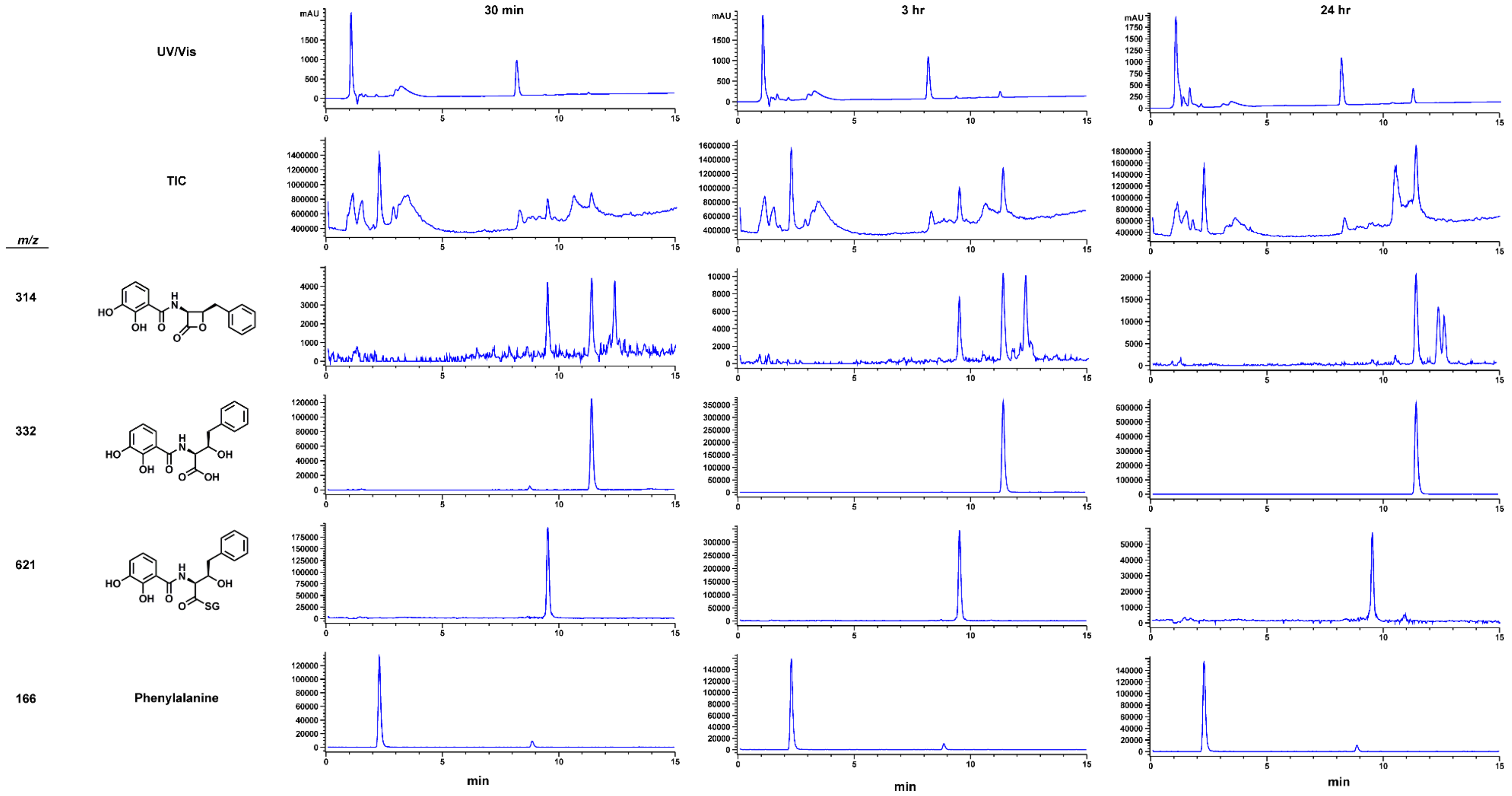

ObiF_R1190A_2

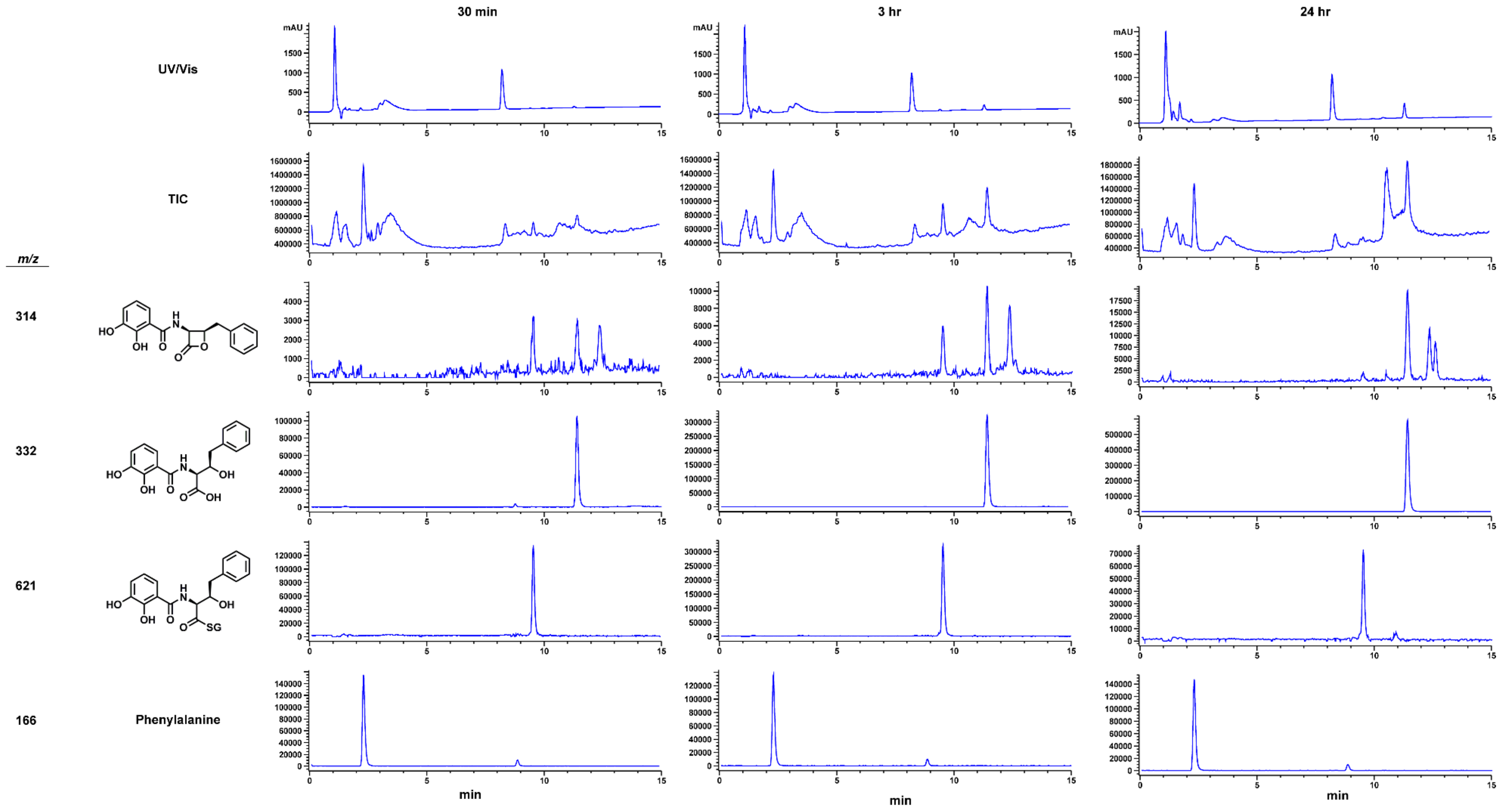

ObiF_H1284A_1

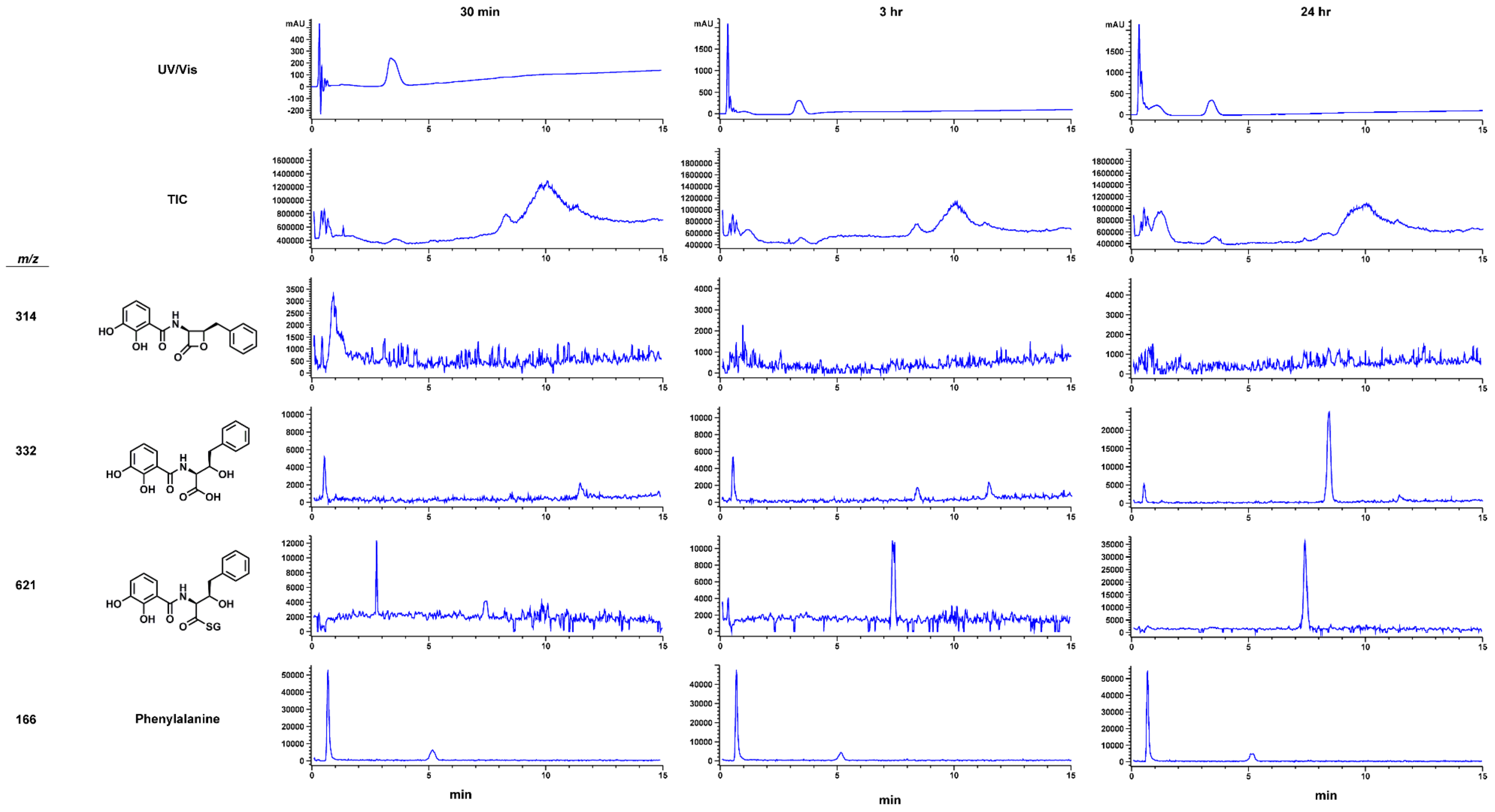

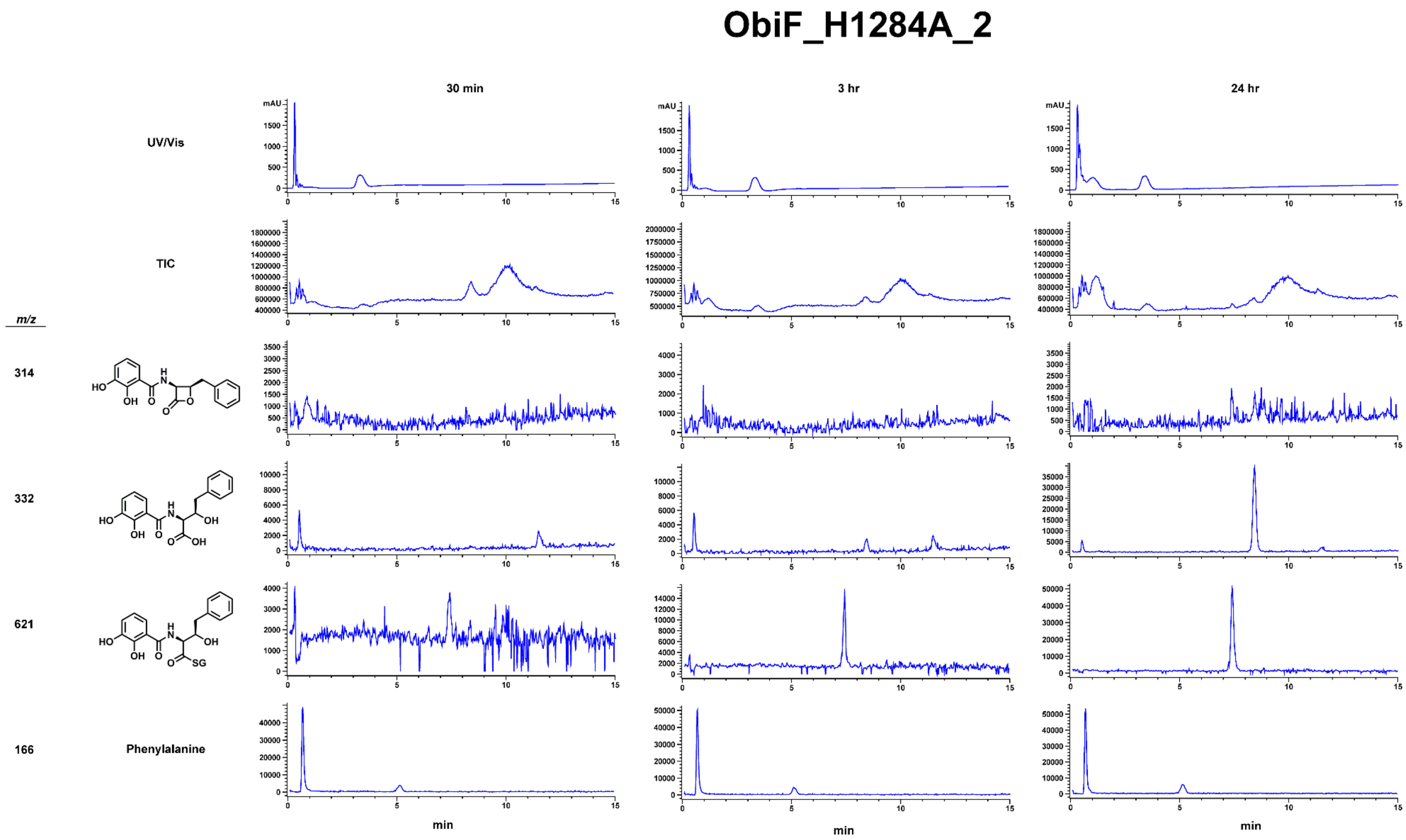

ObiF_H1284A_2
30 min
3 hr
24 hr
UV/Vis
TIC
m/z
314
332
621
166
Phenylalanine
min

ObiF_D1254A_H1284A_1

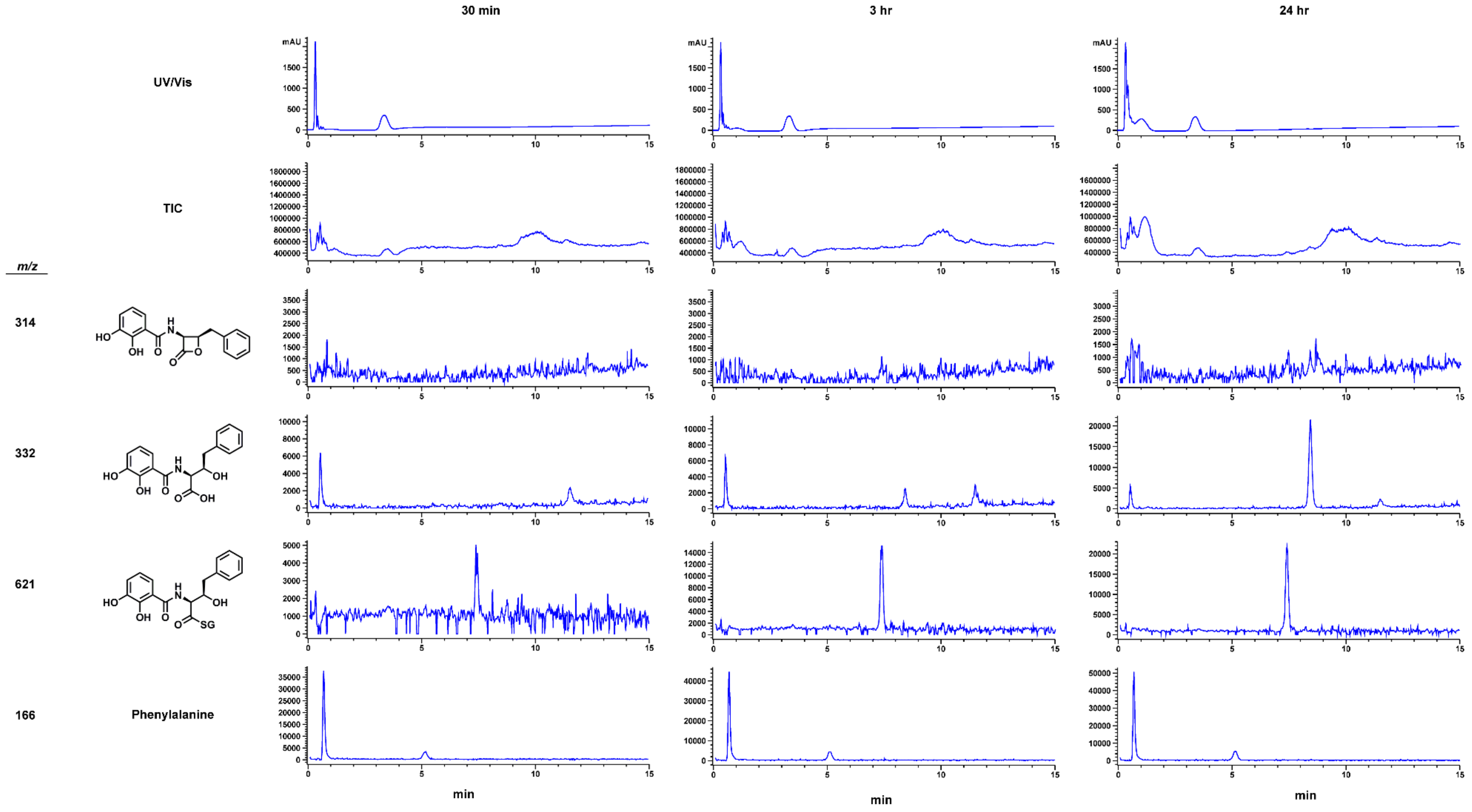

ObiF_D1254A_H1284A_2

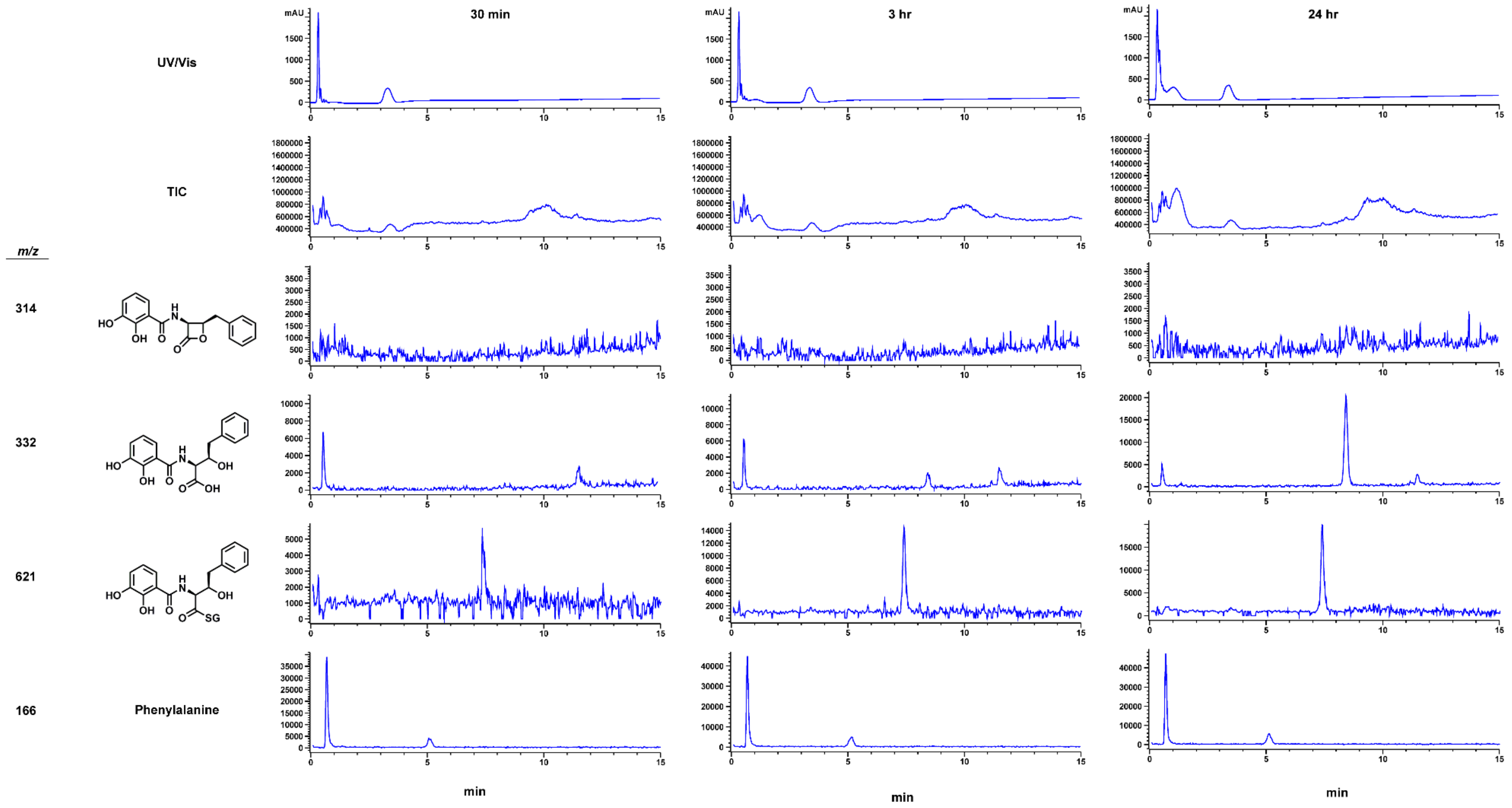

ObiF_G1173L_1

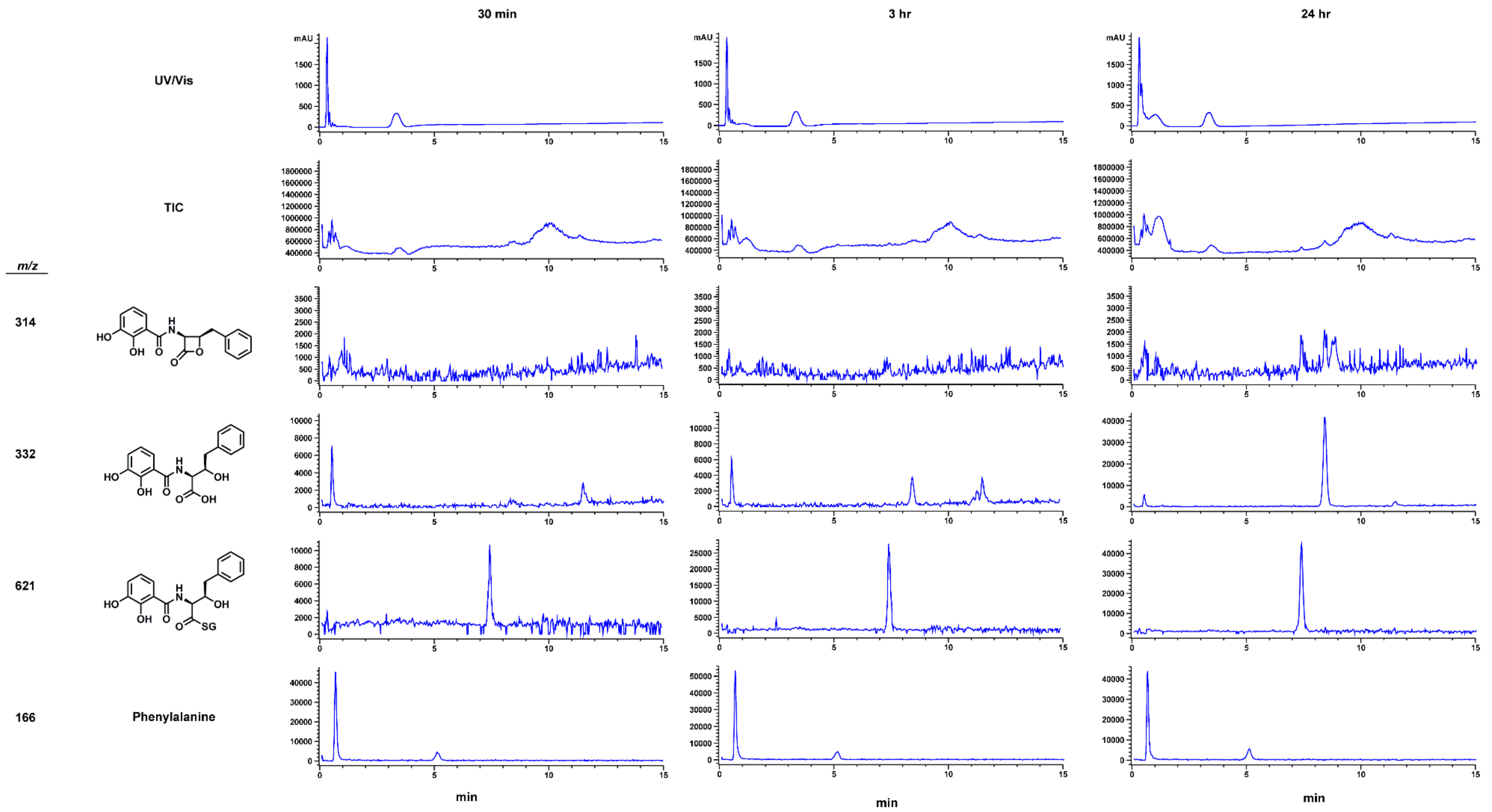

ObiF_G1173L_2

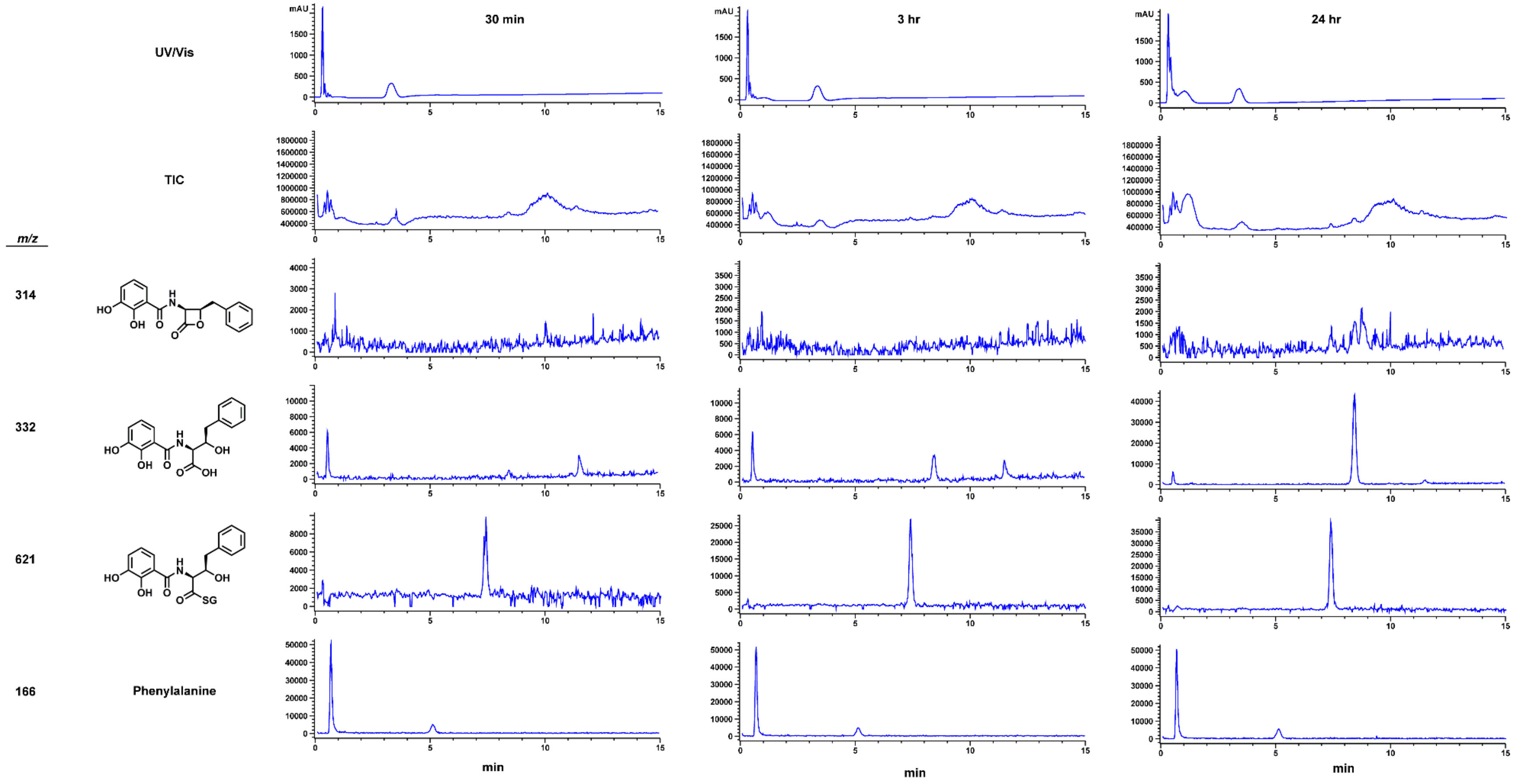

obiF$_1$_A841E_1

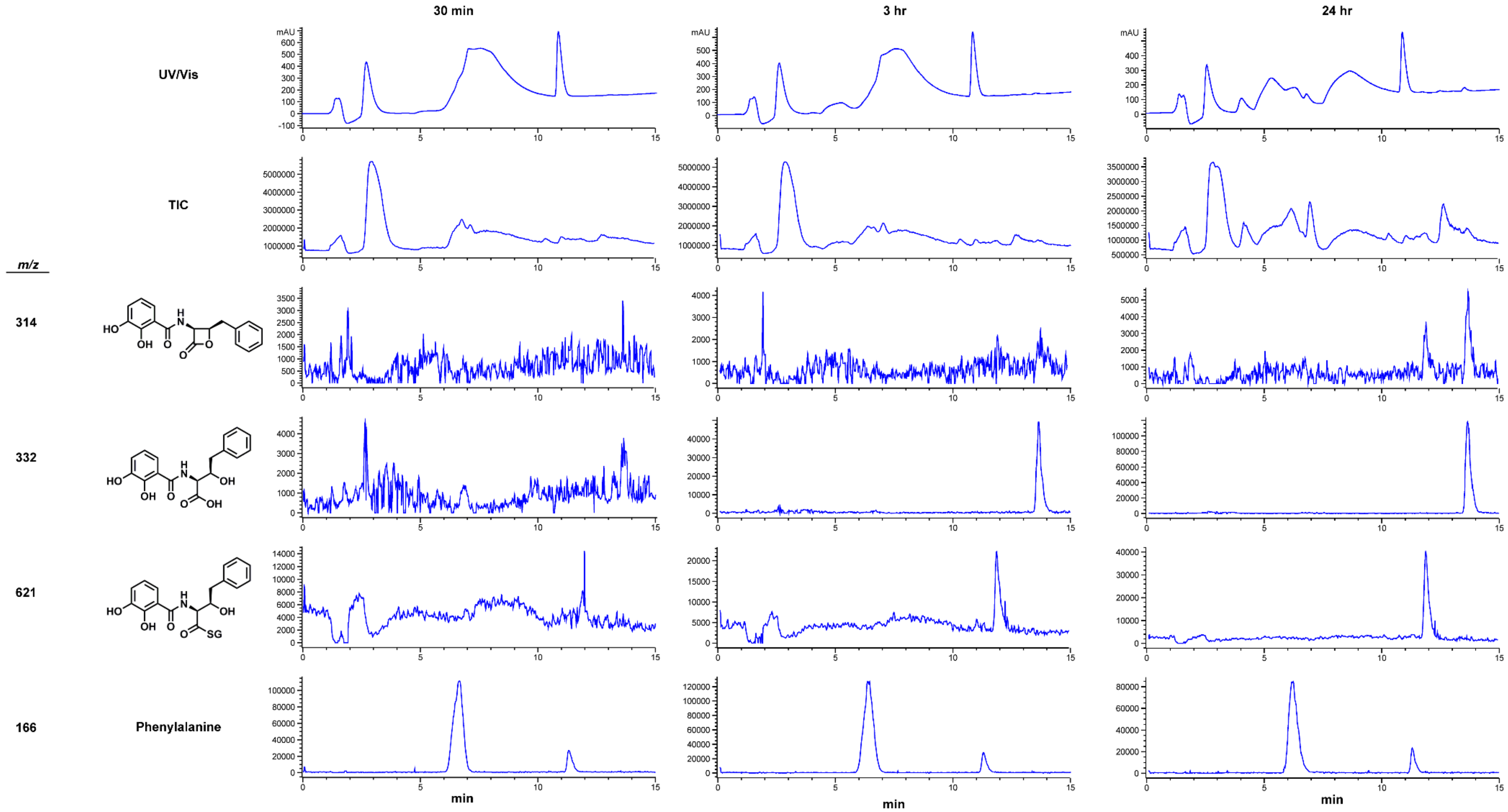

obiF$_1$_A841E_2

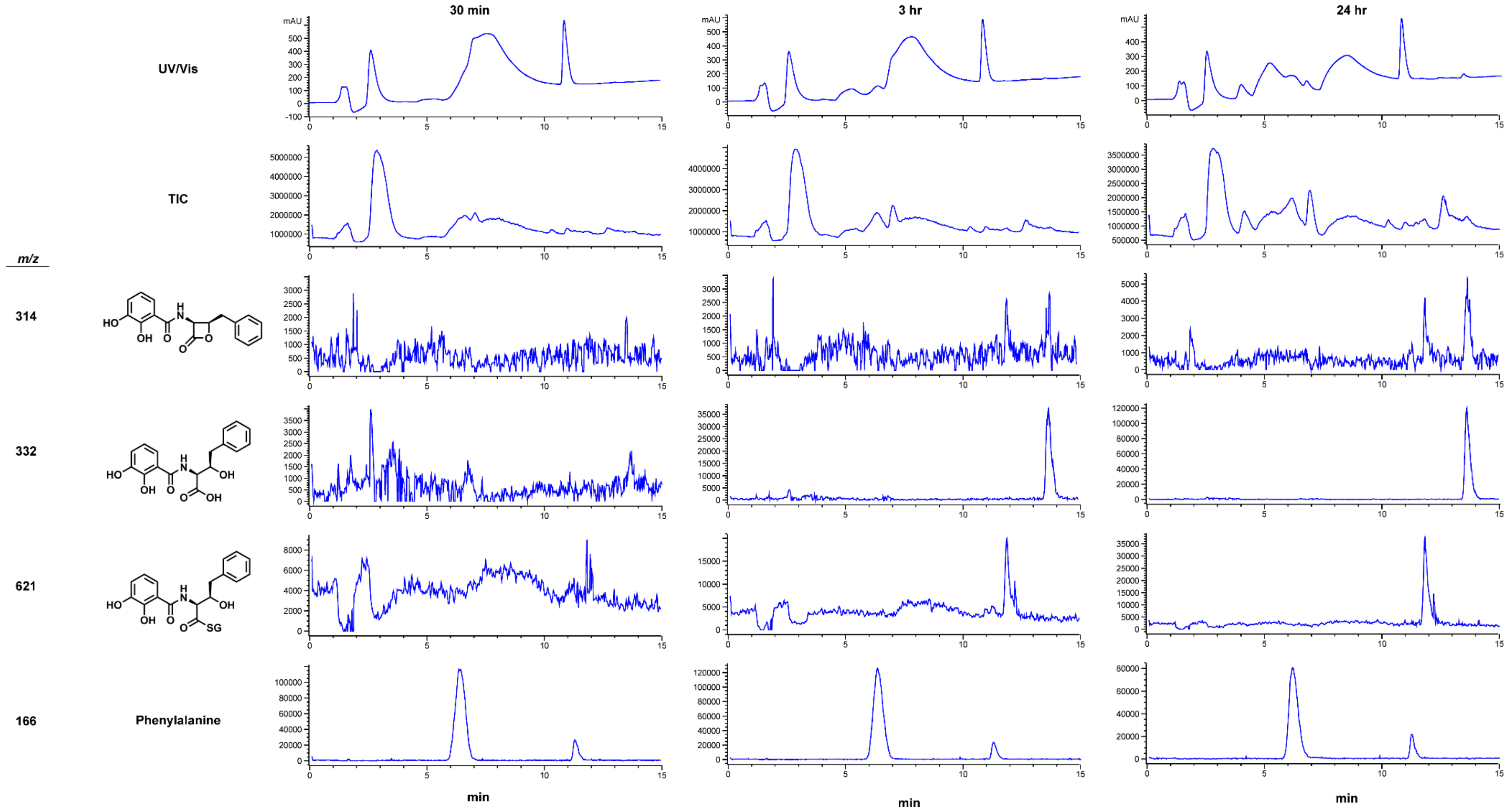

ObiF_C1146S_1

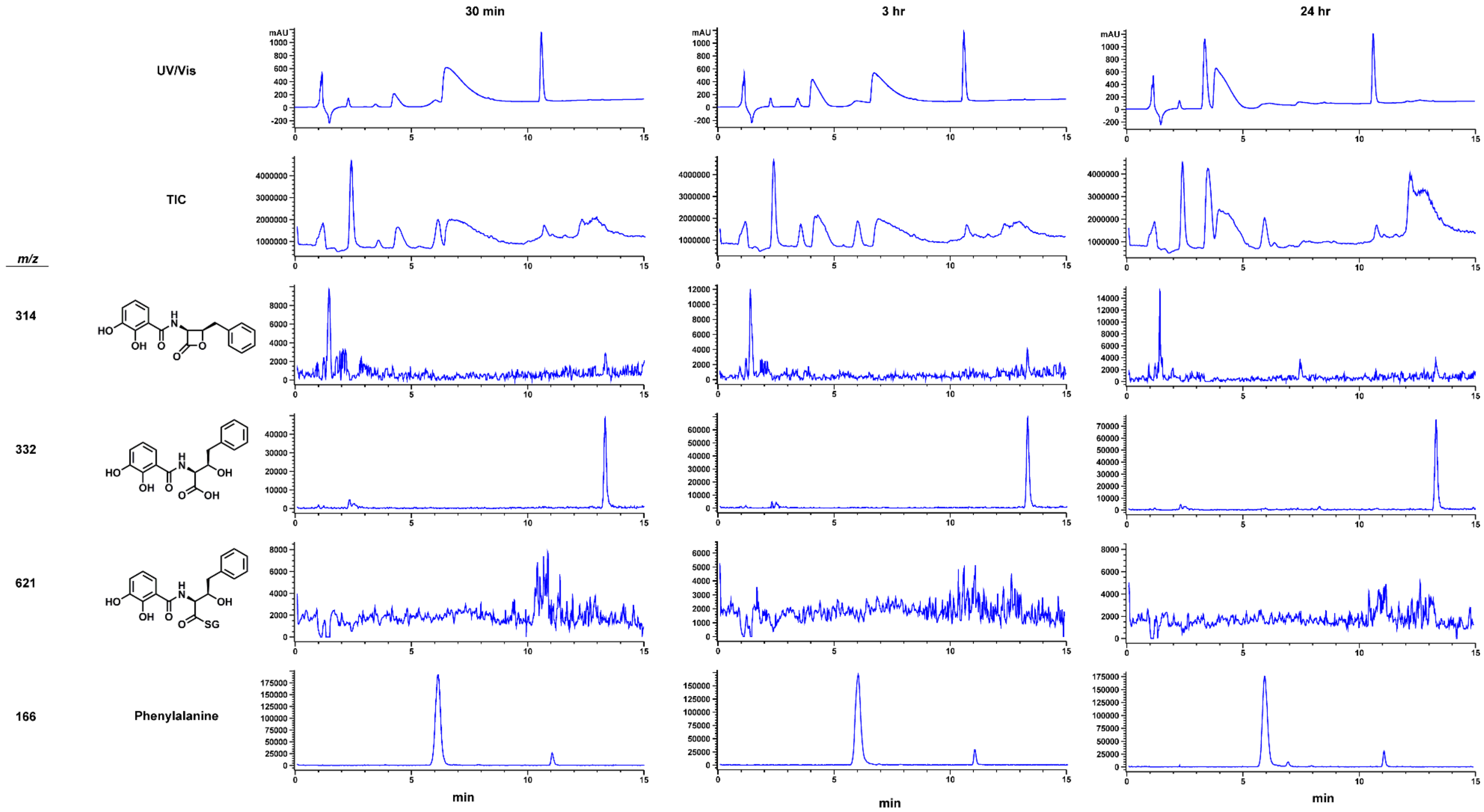

ObiF_C1146S_2

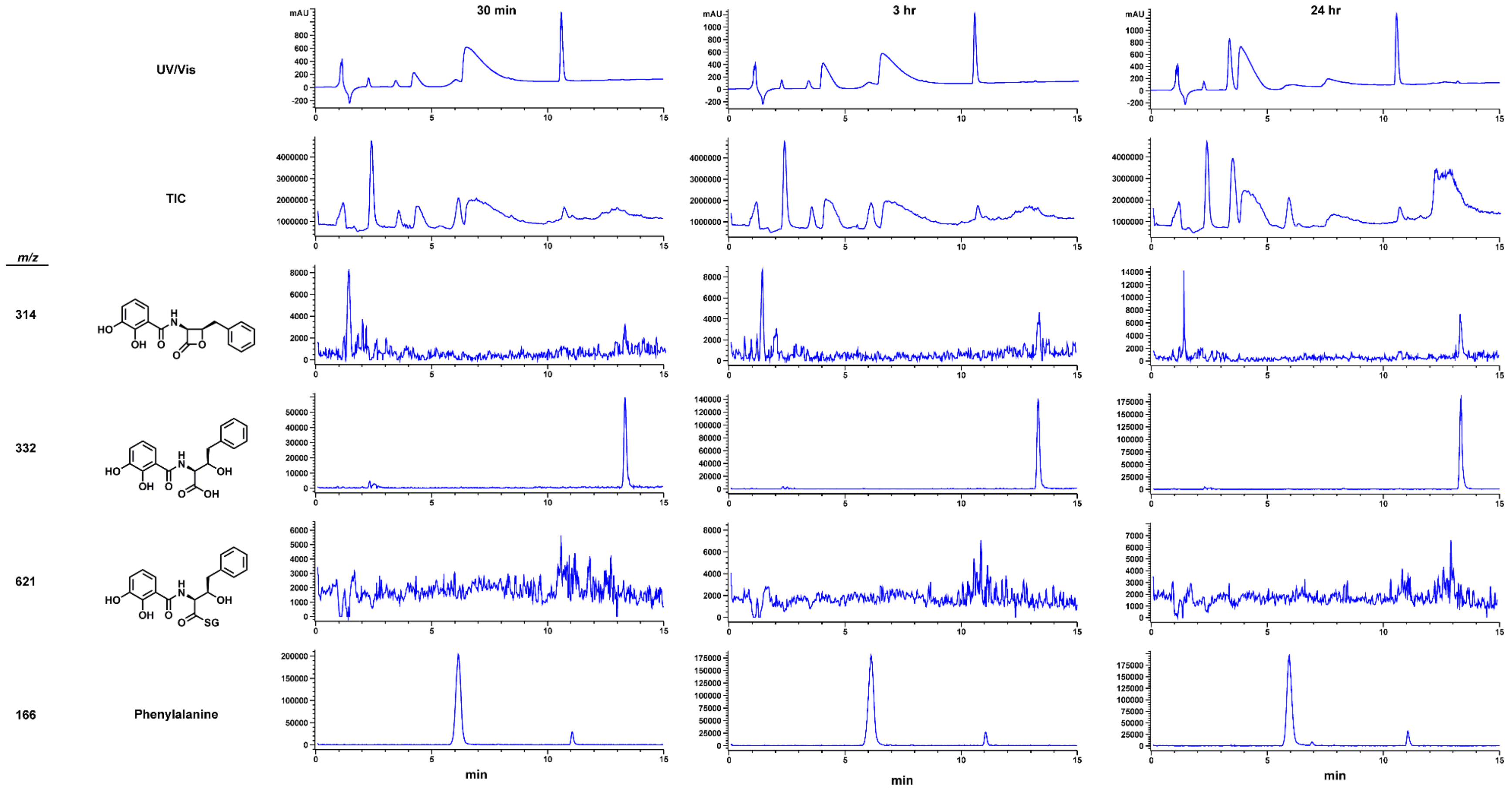

obiF$_1$_C1146A_1

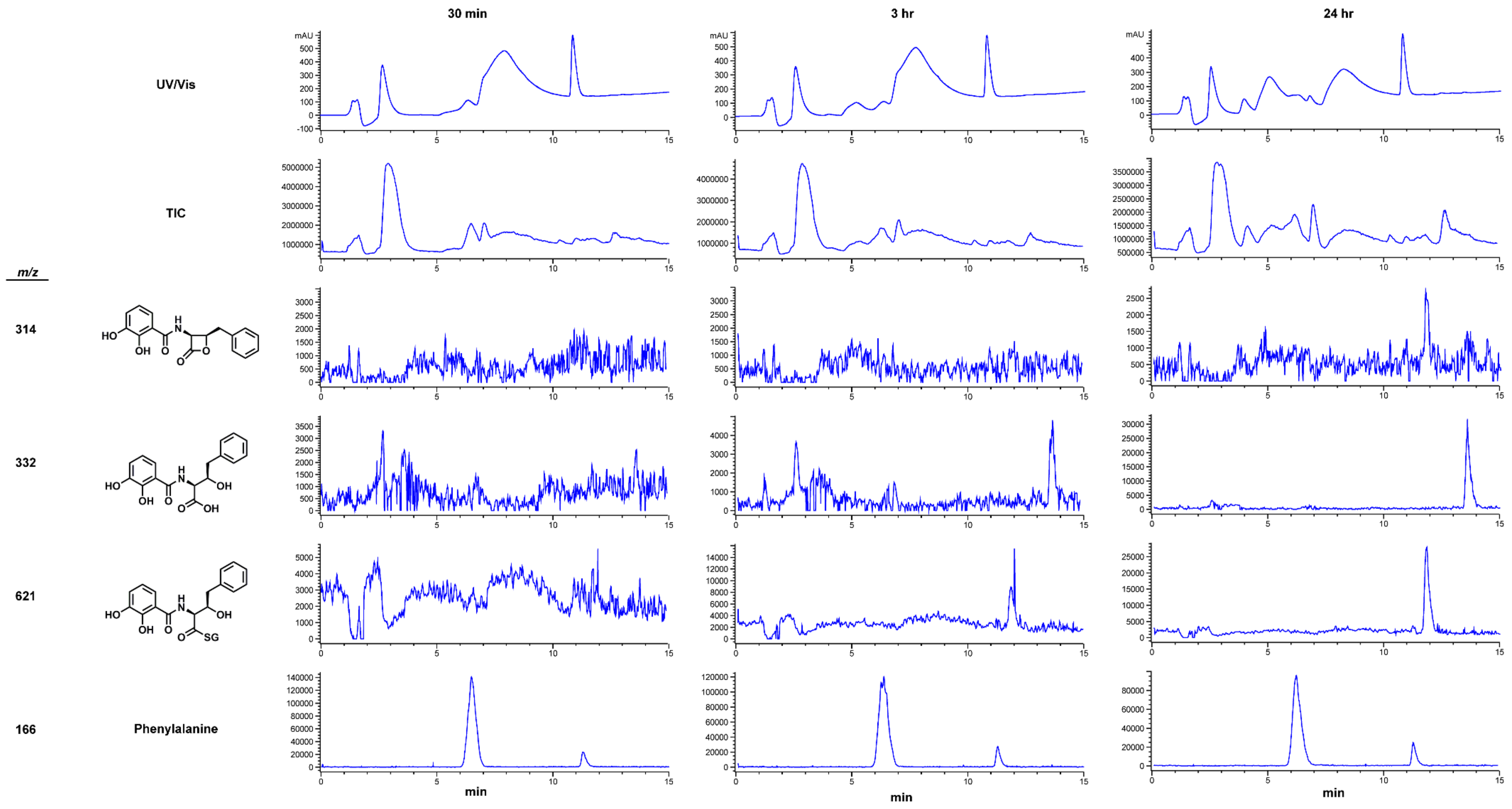

obiF$_1$_C1146A_2

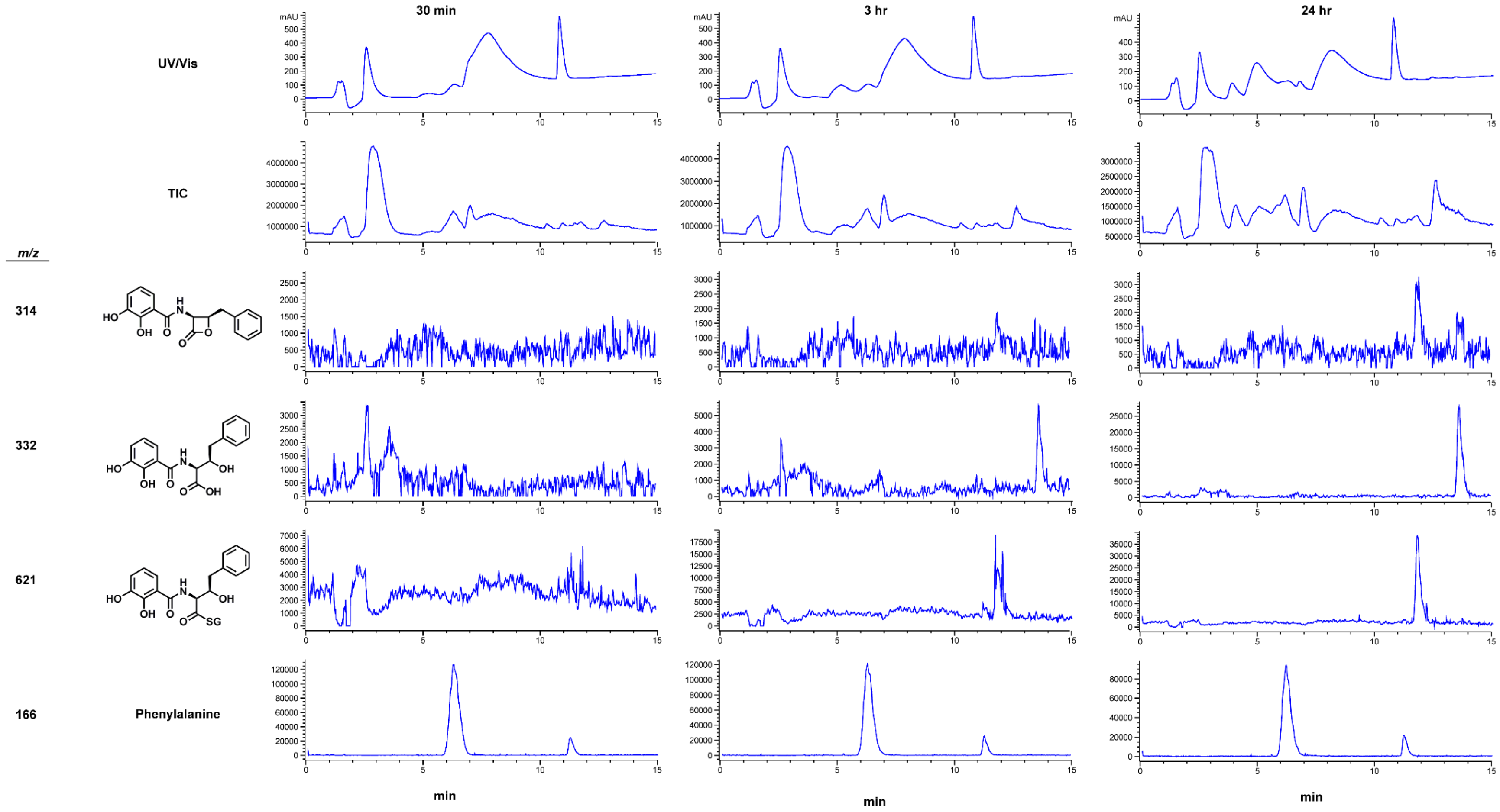

obiF$_1$_del1304_1

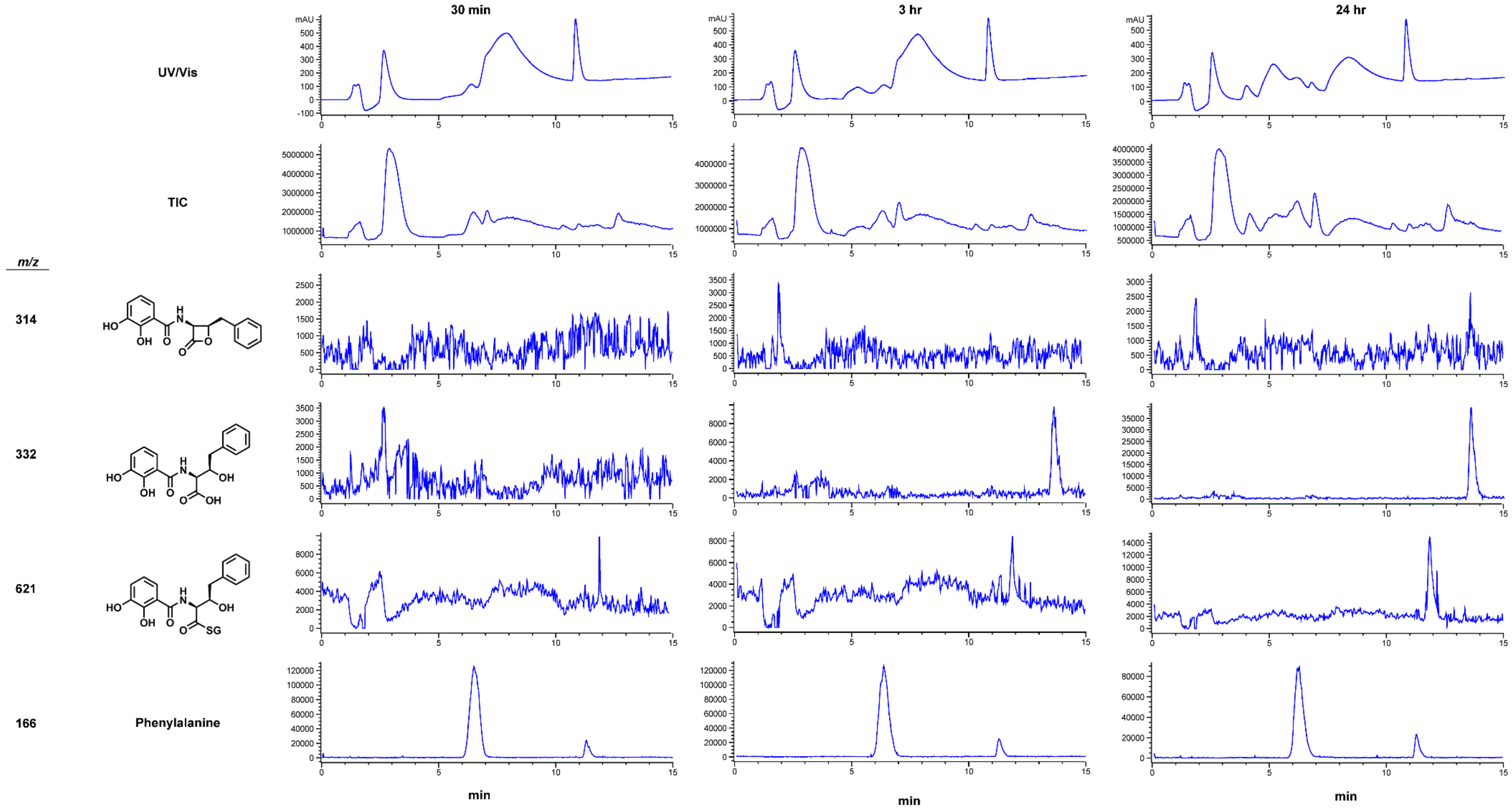

obiF$_1$_del 1304_2

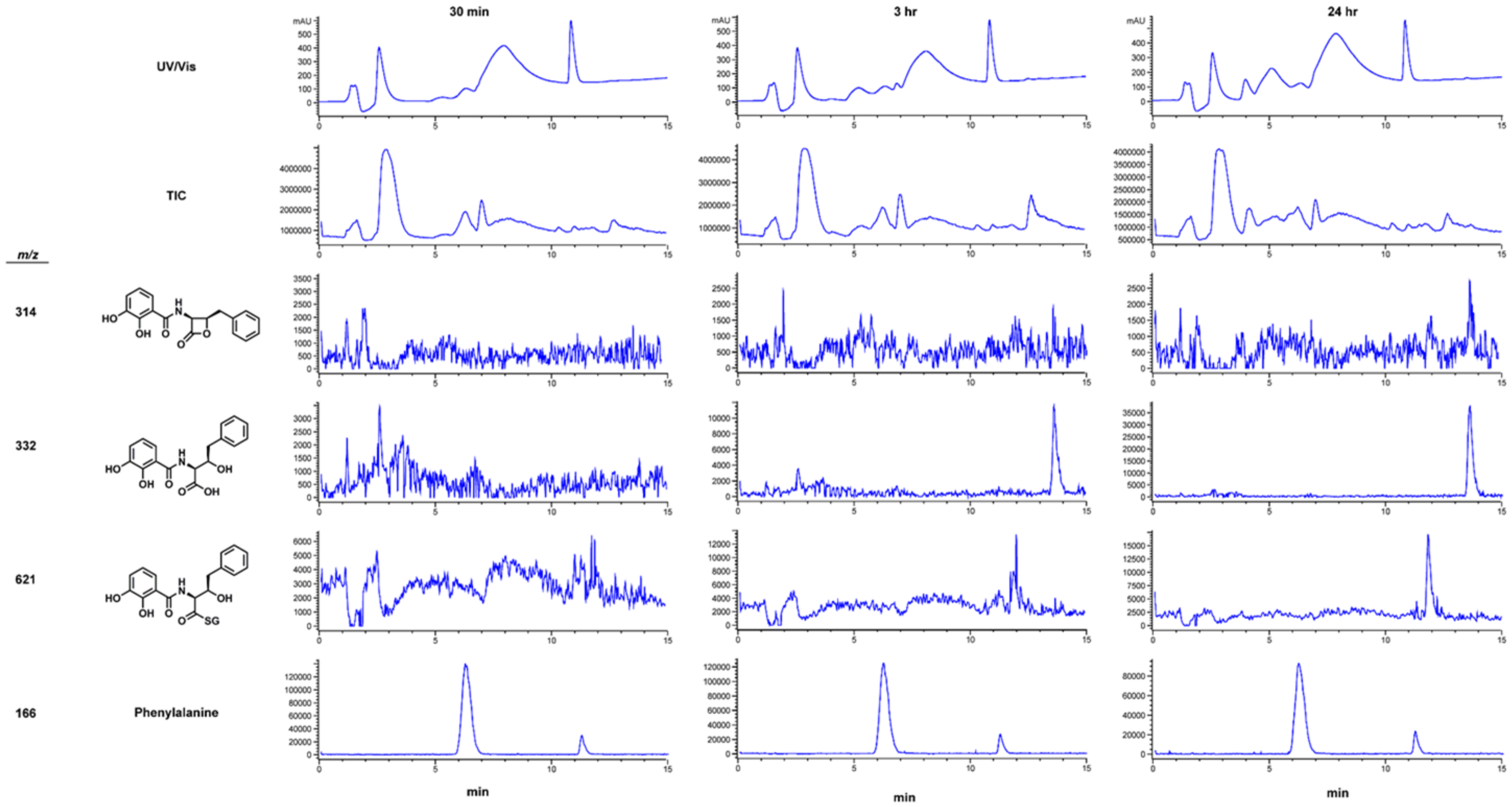

obiF$_1$_rbs 1303/1304_1

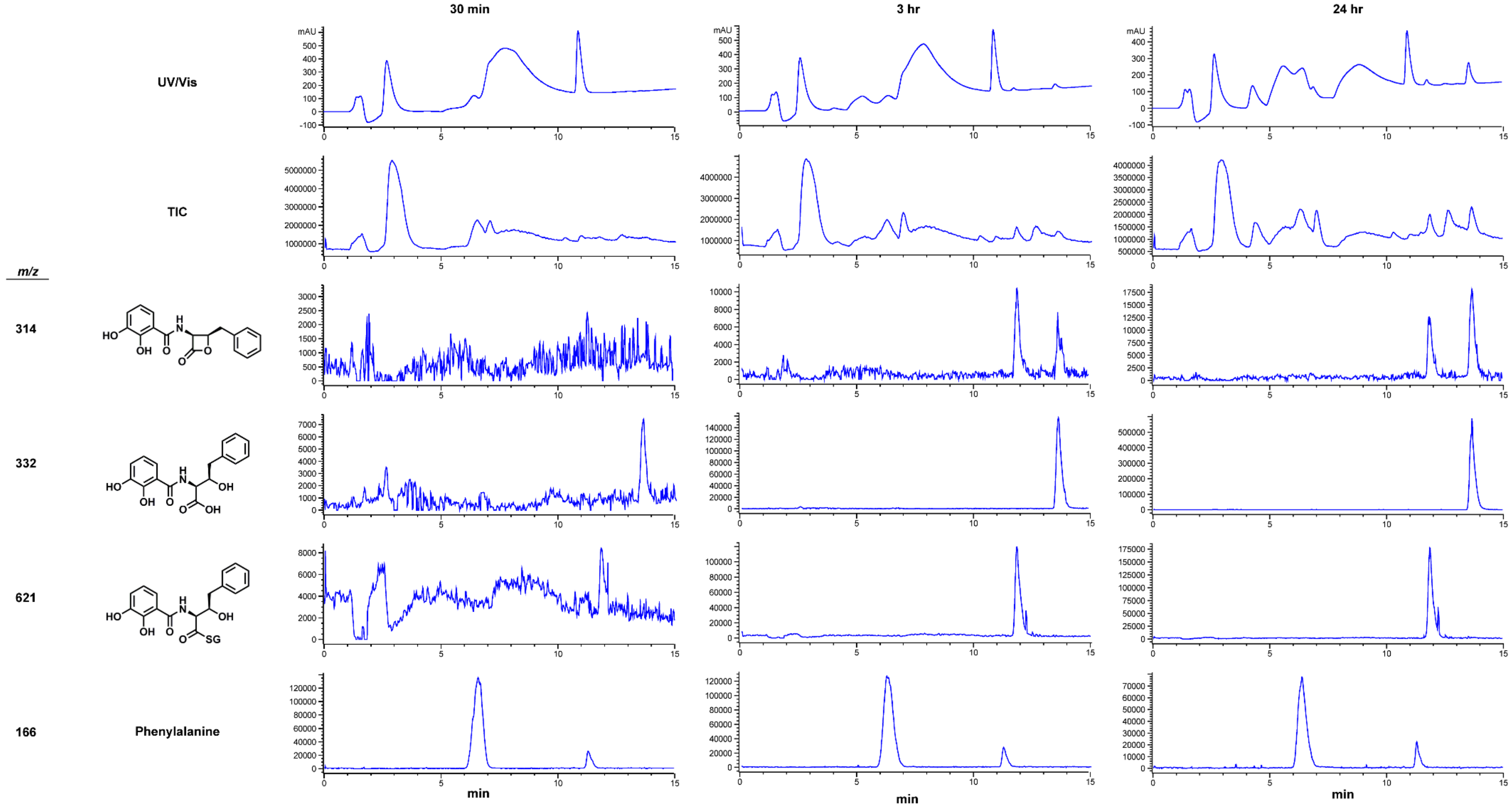

obiF$_1$_rbs 1303/1304_2

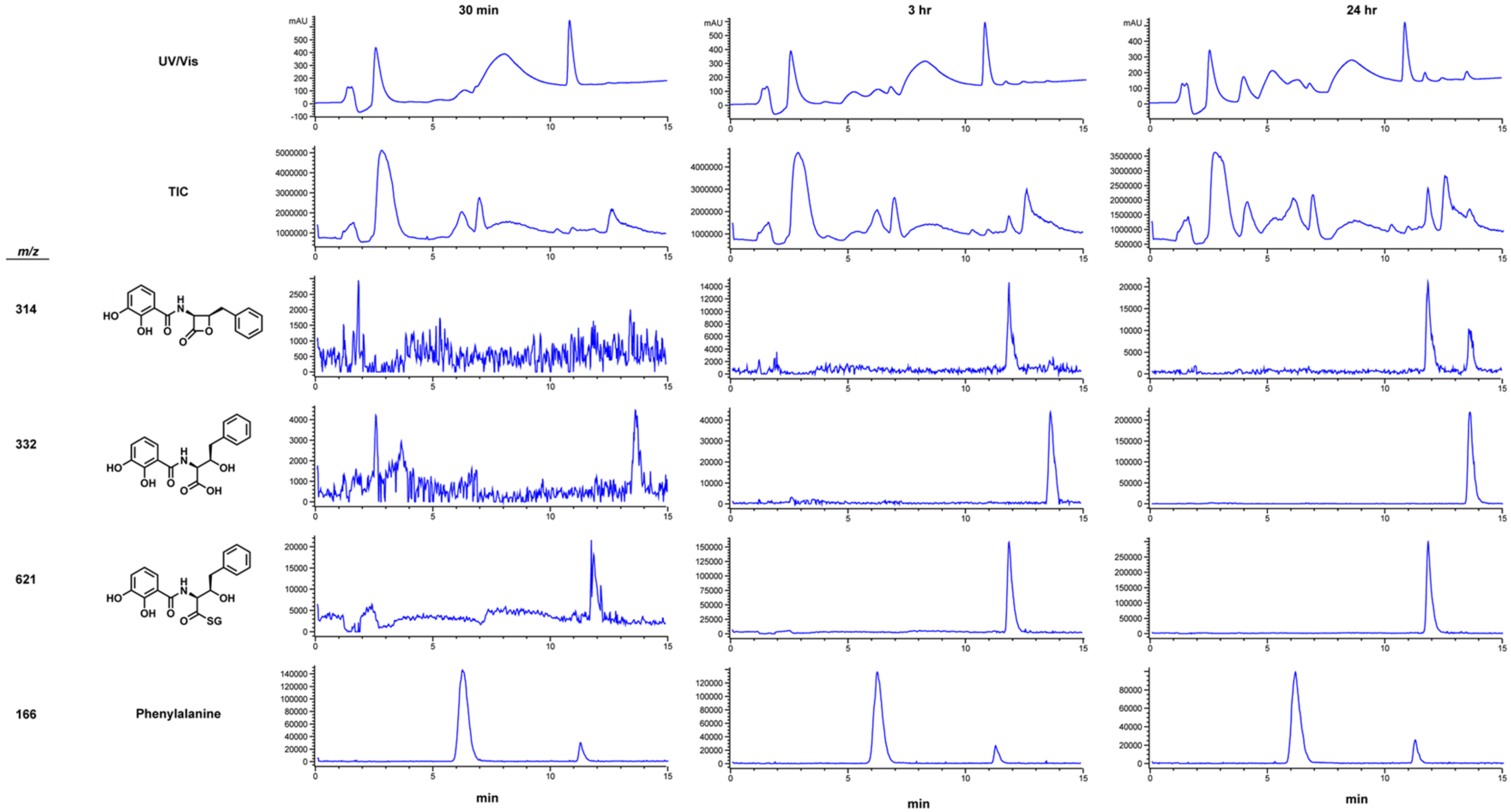

LCMS chromatograms of ObiF mutant assays for coupled ObiHDF1F2 enzyme reactions with 2,3-DHB (3a) and phenylacetaldehyde (1a) as substrates and variable concentrations of added ObiF1-MLP domain.

obiF$_1$_WT_1_MLP (0 µM)

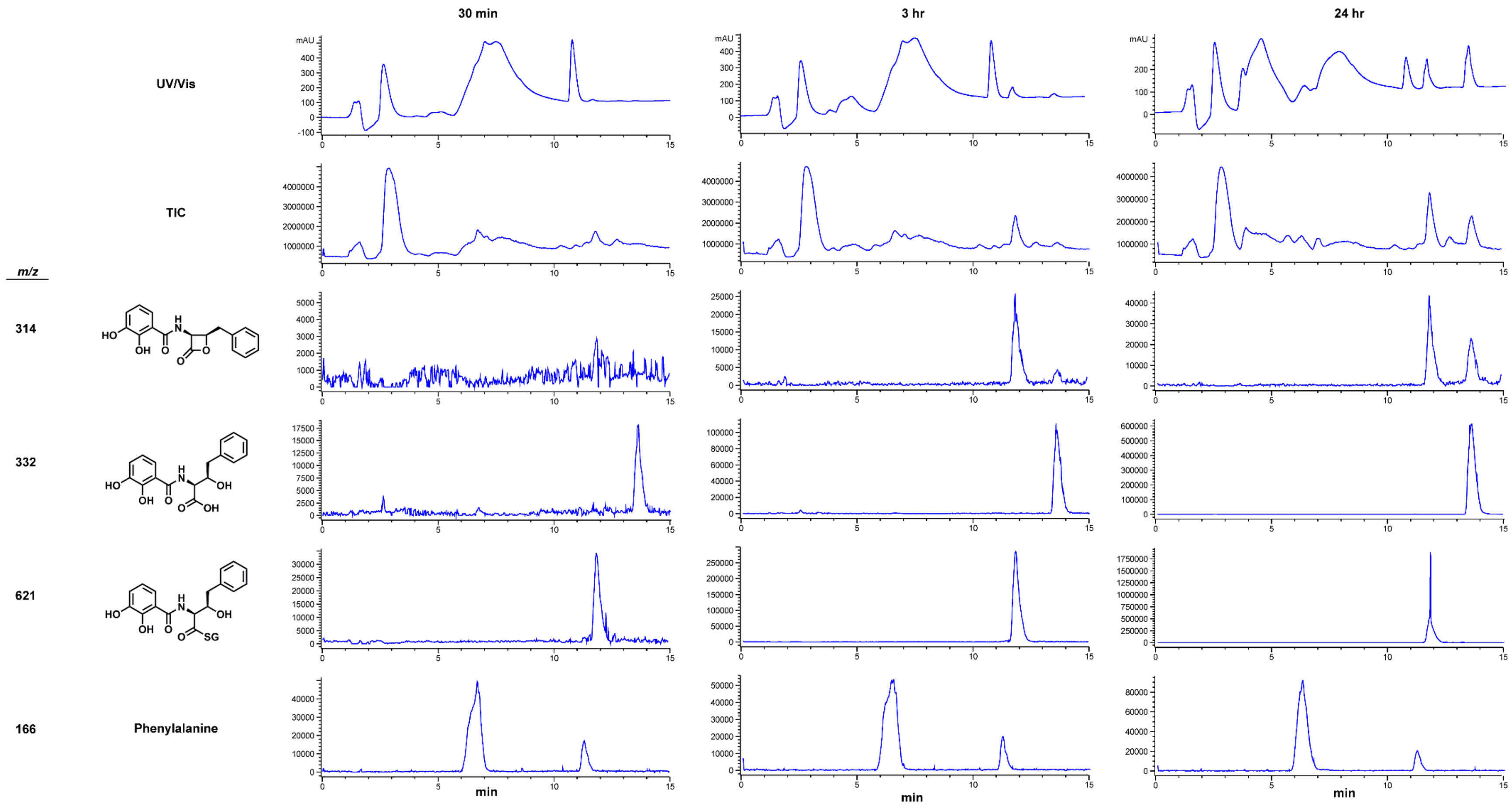

obiF$_1$_WT_2_MLP (0 μM)

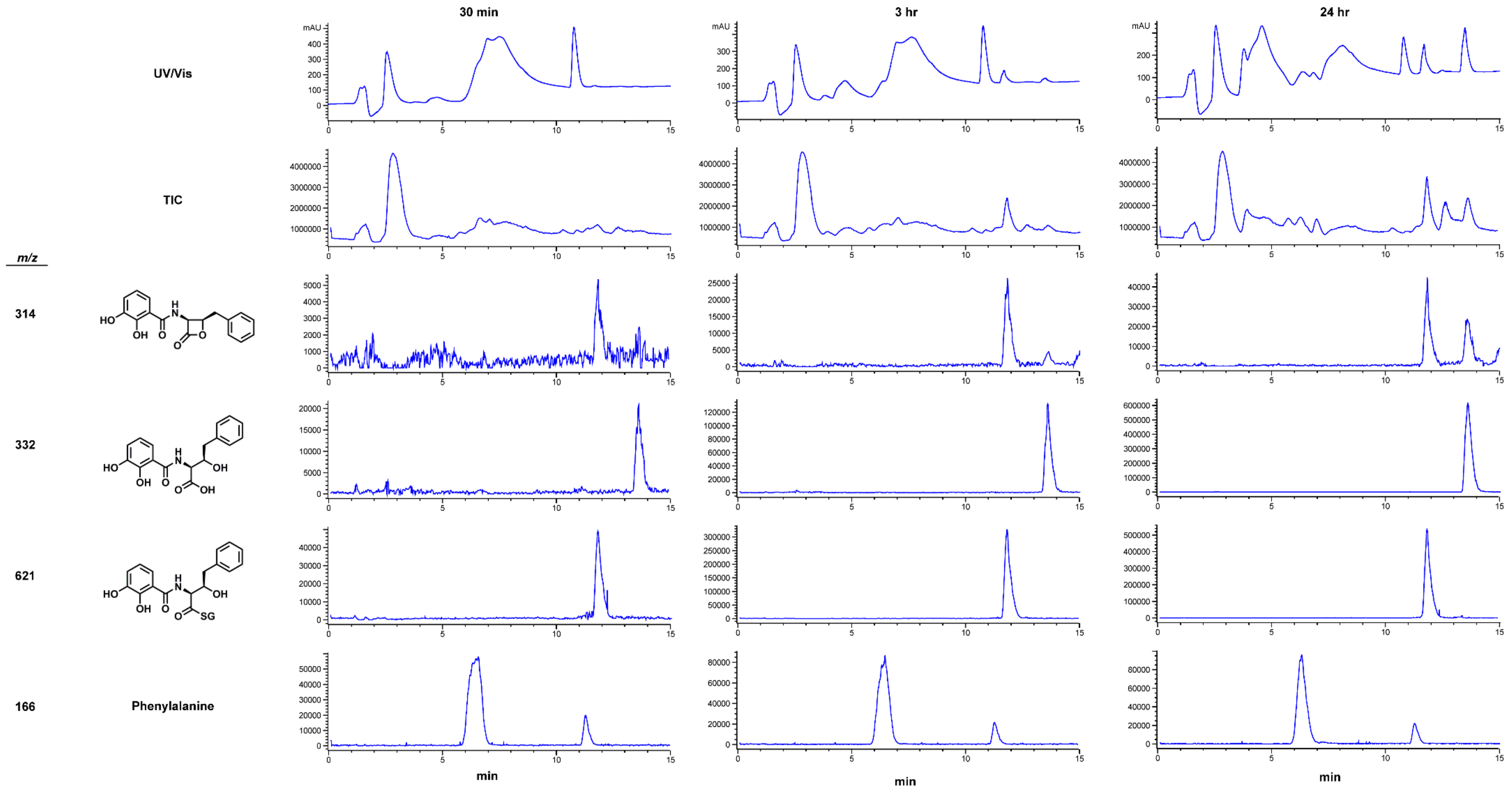

obiF$_1$_WT_3_MLP (0 μM)

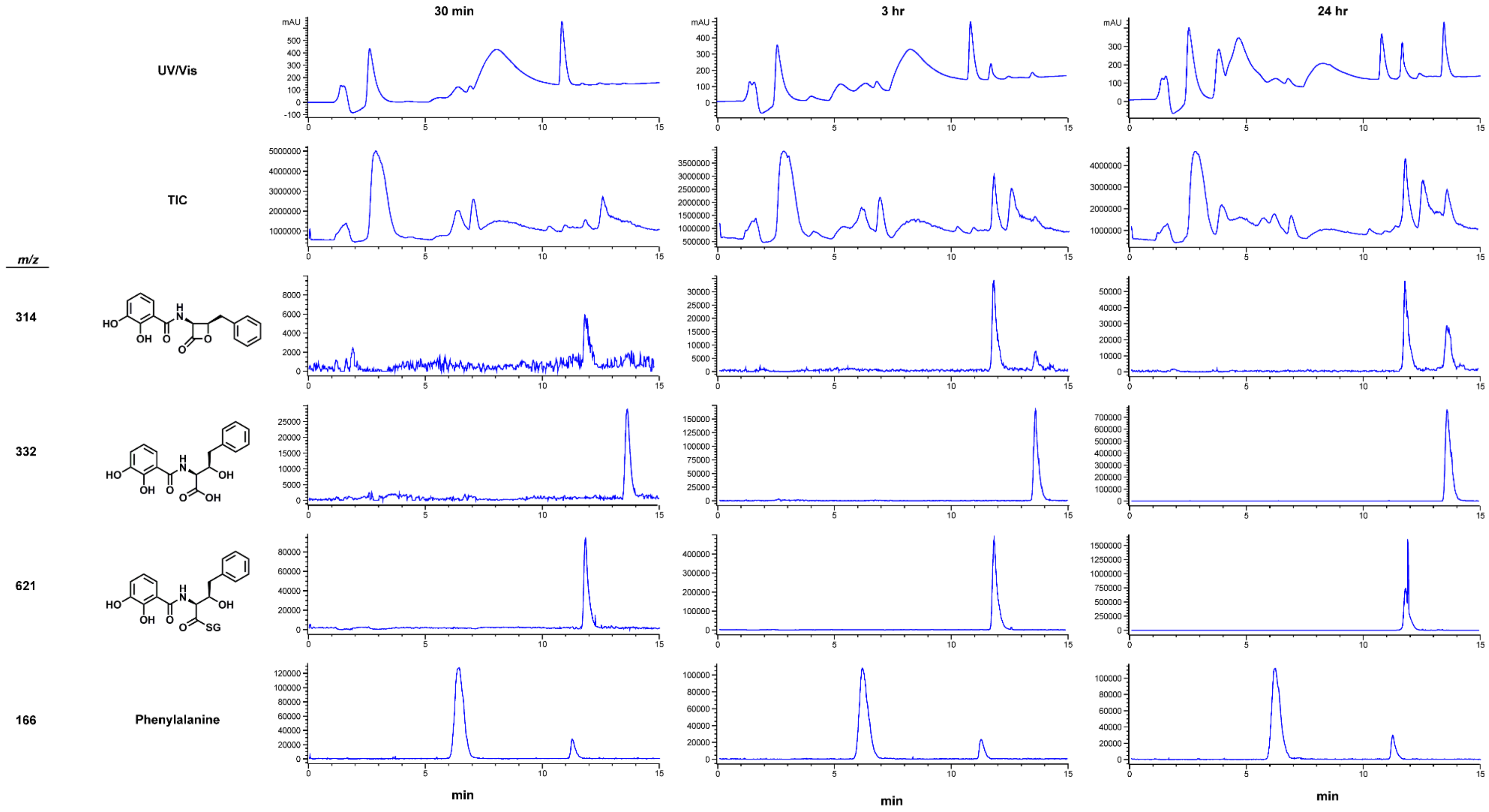

obiF$_1$_WT_4_MLP (0 µM)

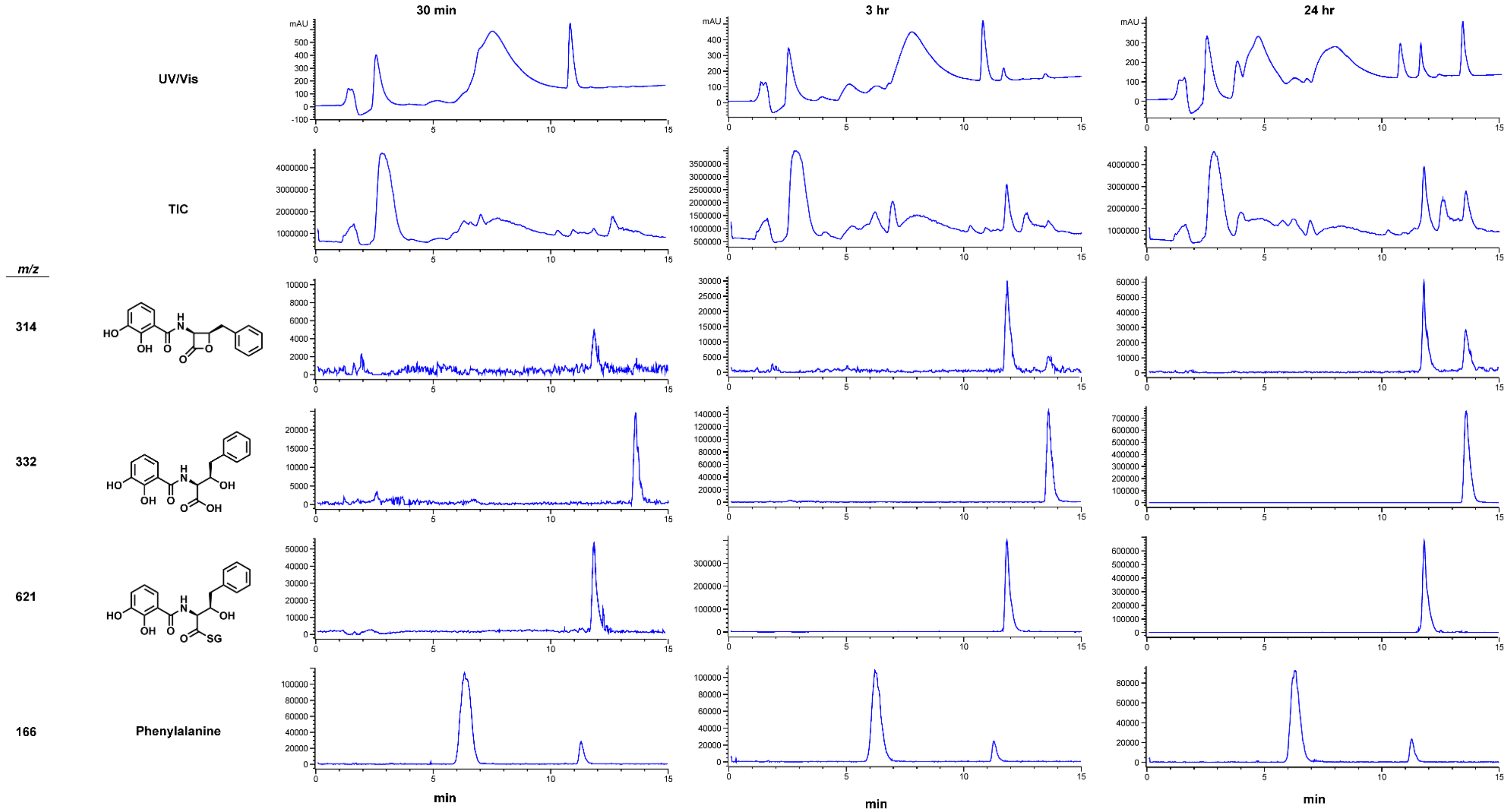

obiF$_1$_WT_1_MLP (1 μM)

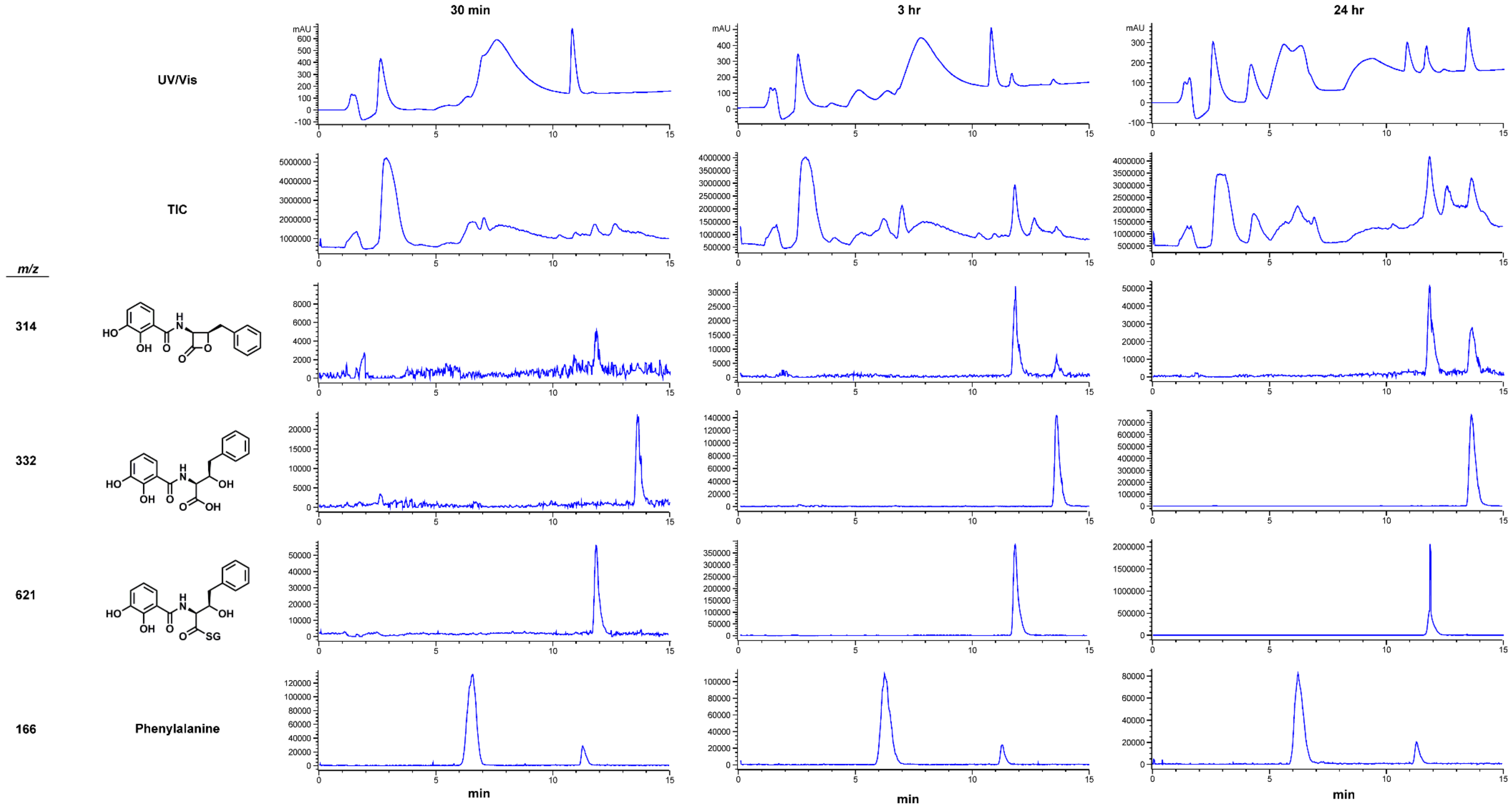

obiF$_1$_WT_2_MLP (1 μM)

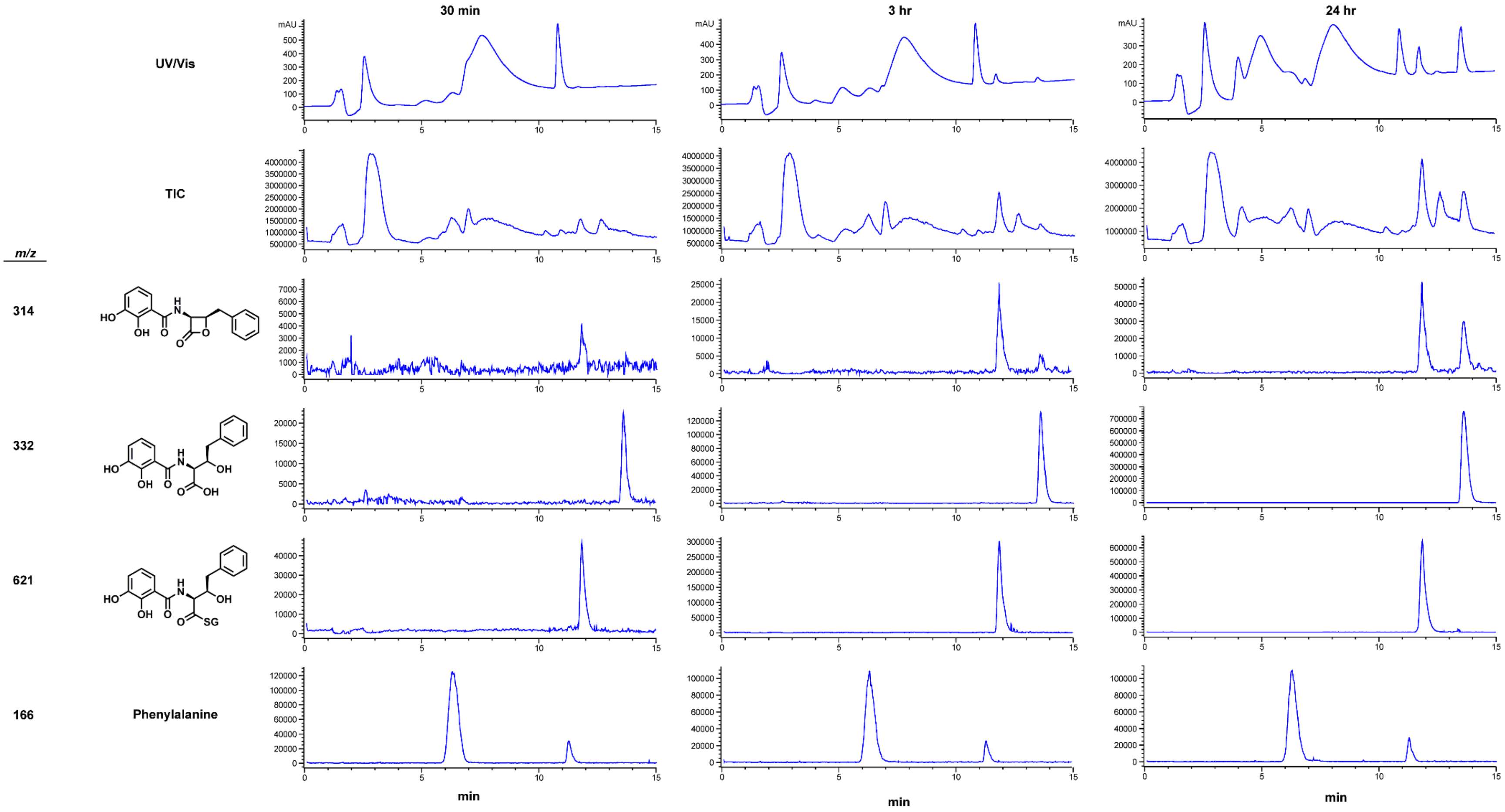

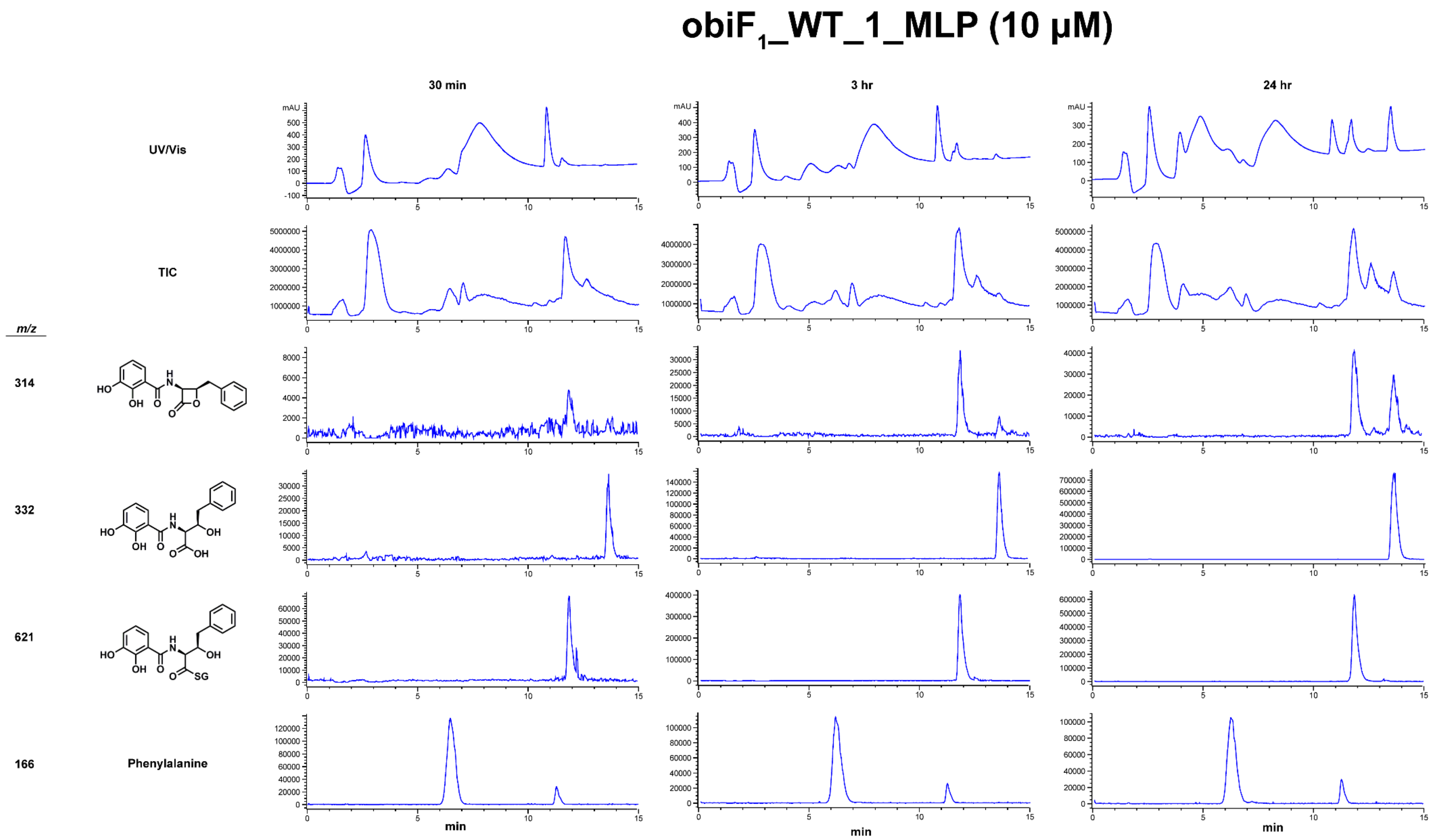

obiF1_WT_1_MLP (10 μM)
30 min
3 hr
24 hr
UV/Vis
TIC
m/z
314
332
621
166
Phenylalanine
mAU
min

obiF$_1$_WT_2_MLP (10 μM)

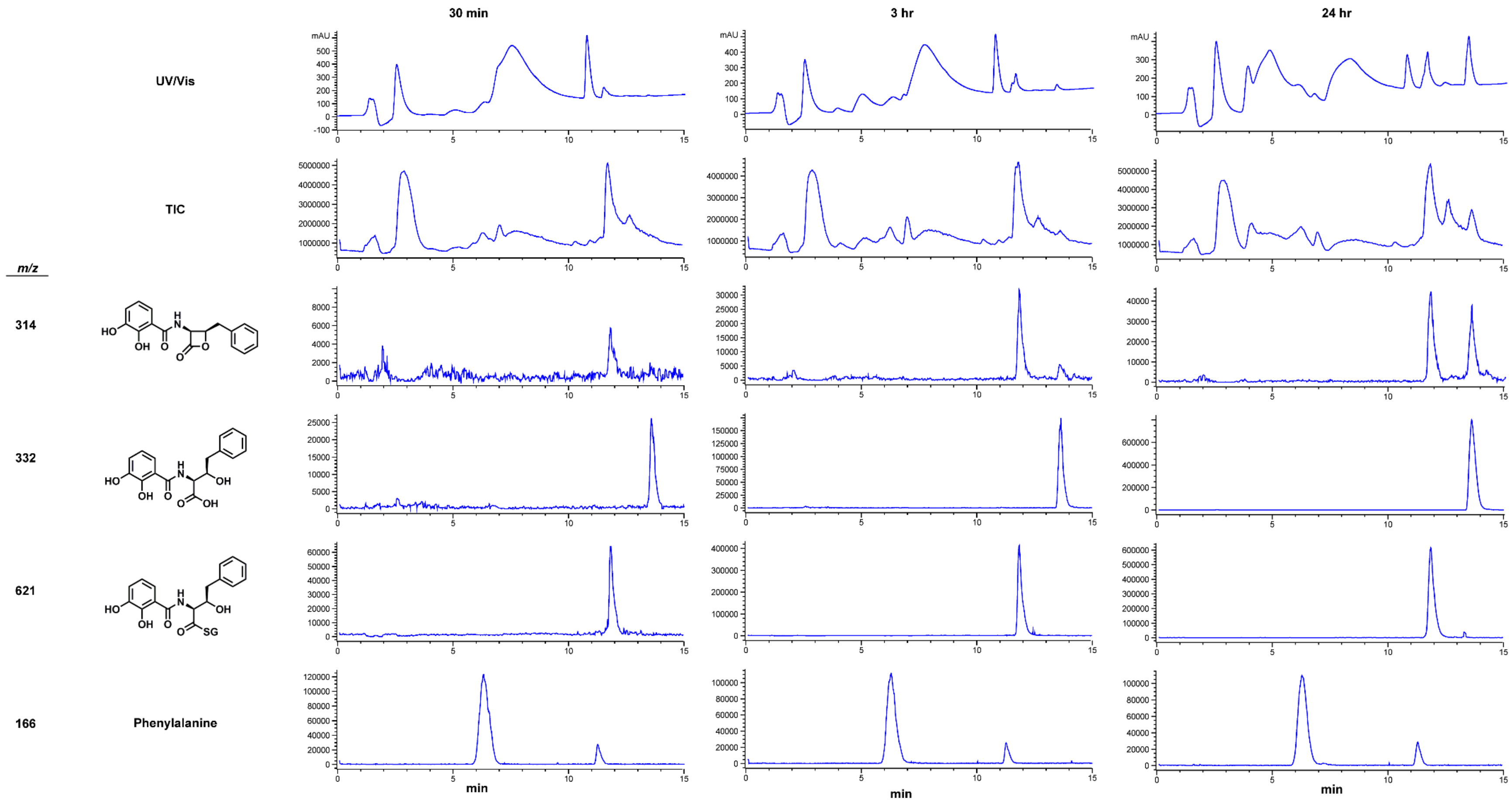

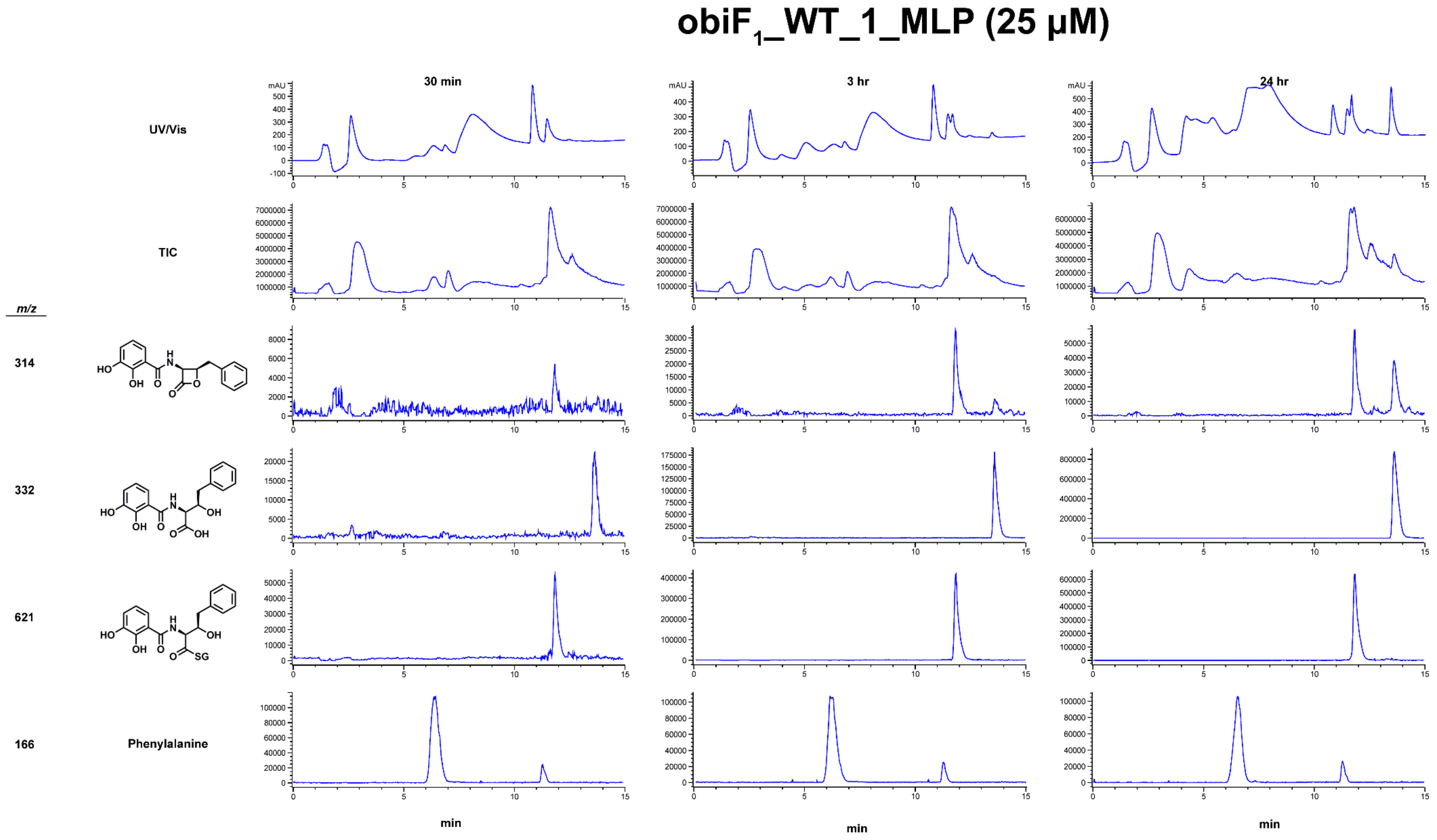
obiF$_1$_WT_1_MLP (25 μM)
30 min
3 hr
24 hr
UV/Vis
TIC
m/z
314
332
621
166
Phenylalanine
mAU
min

obiF$_1$_WT_2_MLP (25 μM)

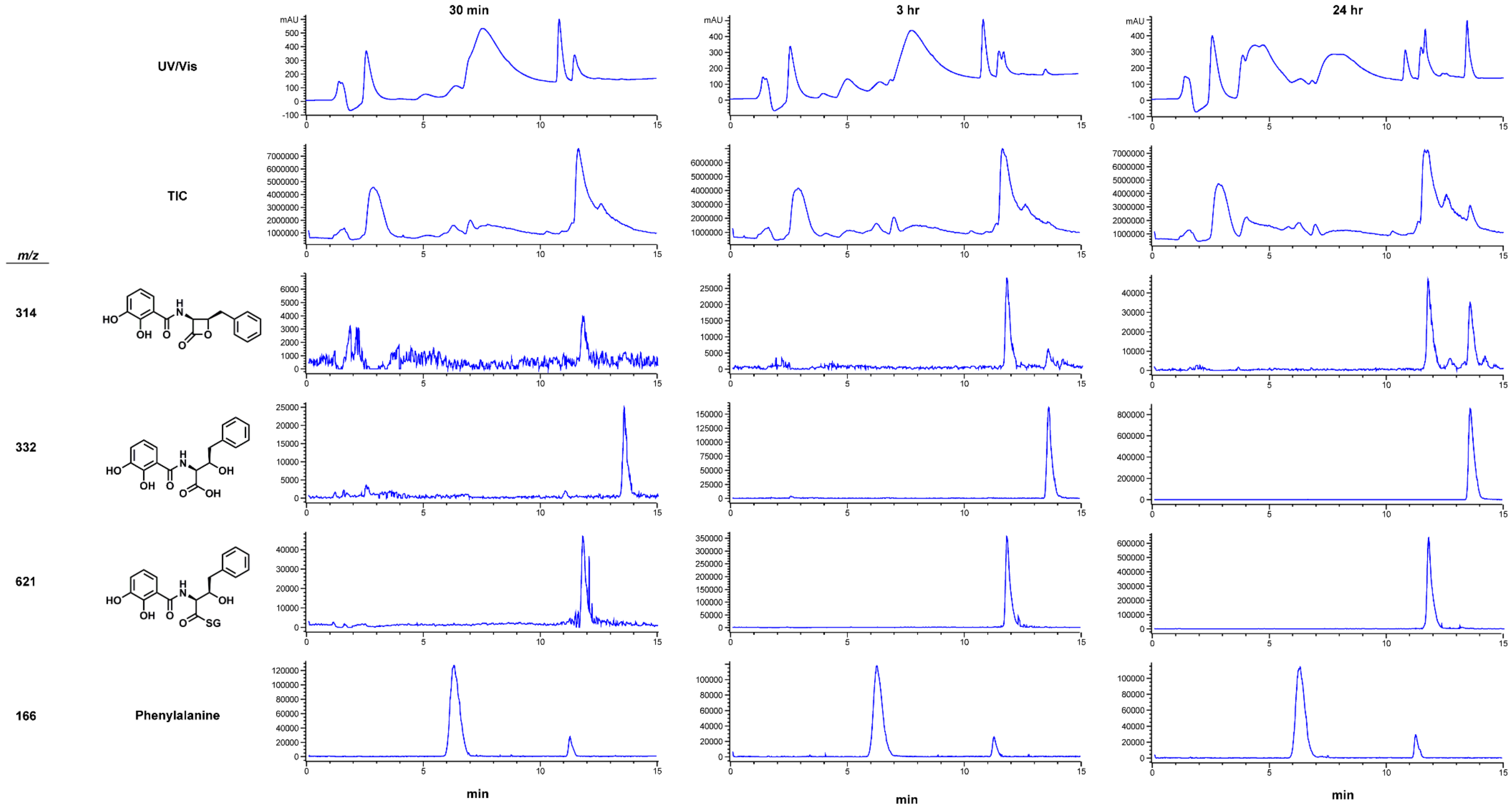

obiF$_1$_del_1_MLP (1 μM)

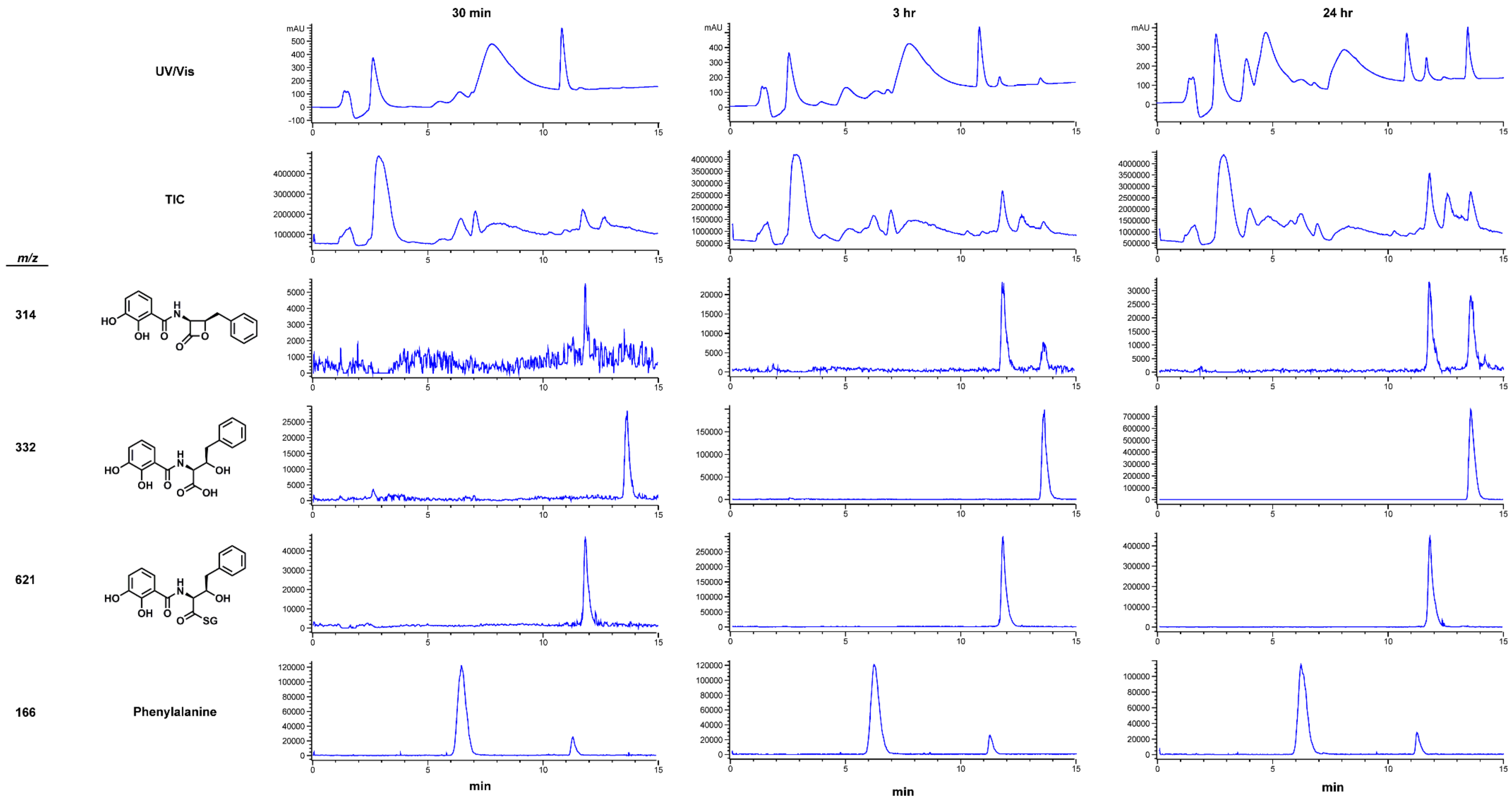

obiF$_1$_del_2_MLP (1 μM)

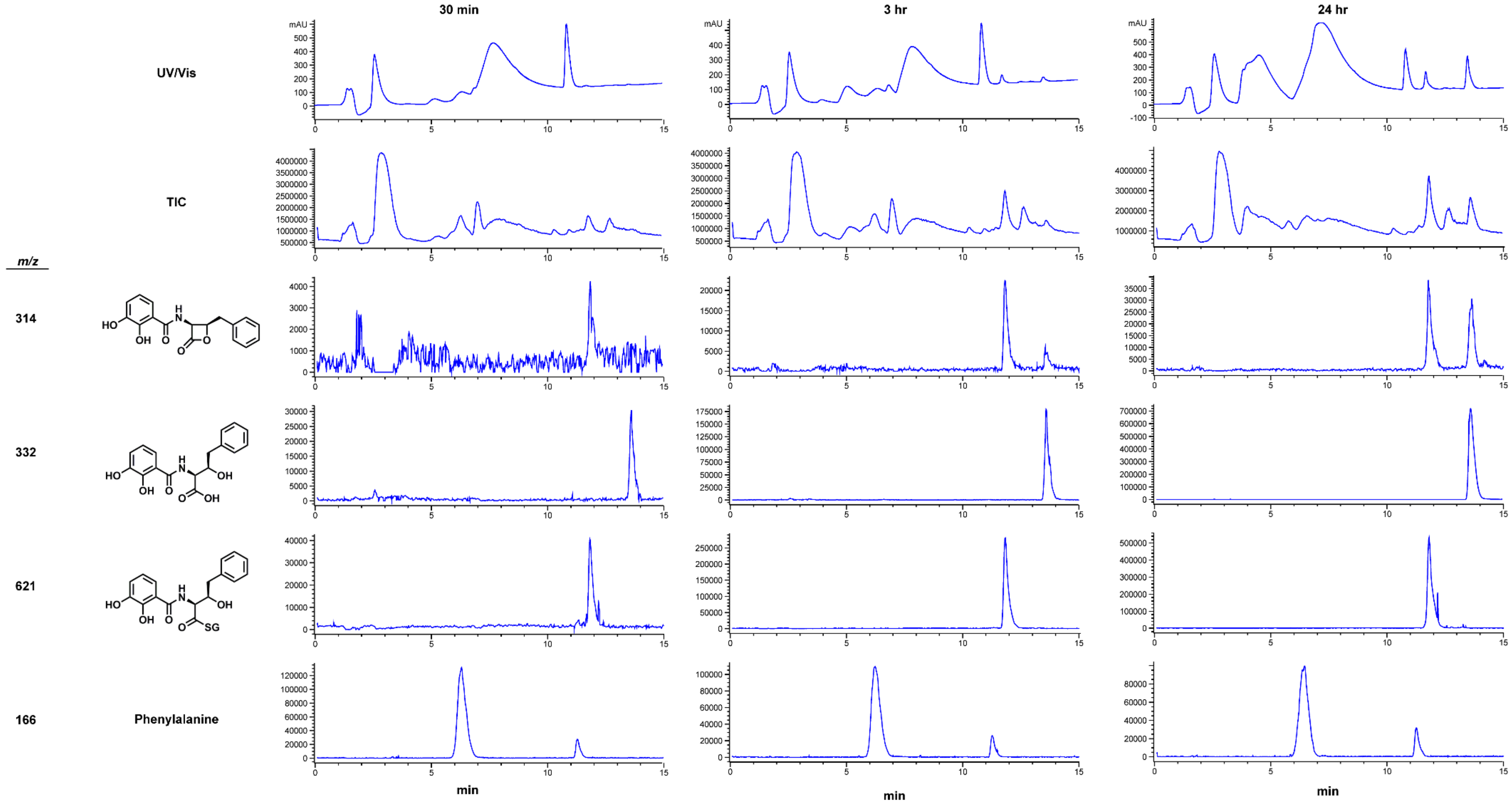

obiF$_1$_del_1_MLP (10 μM)

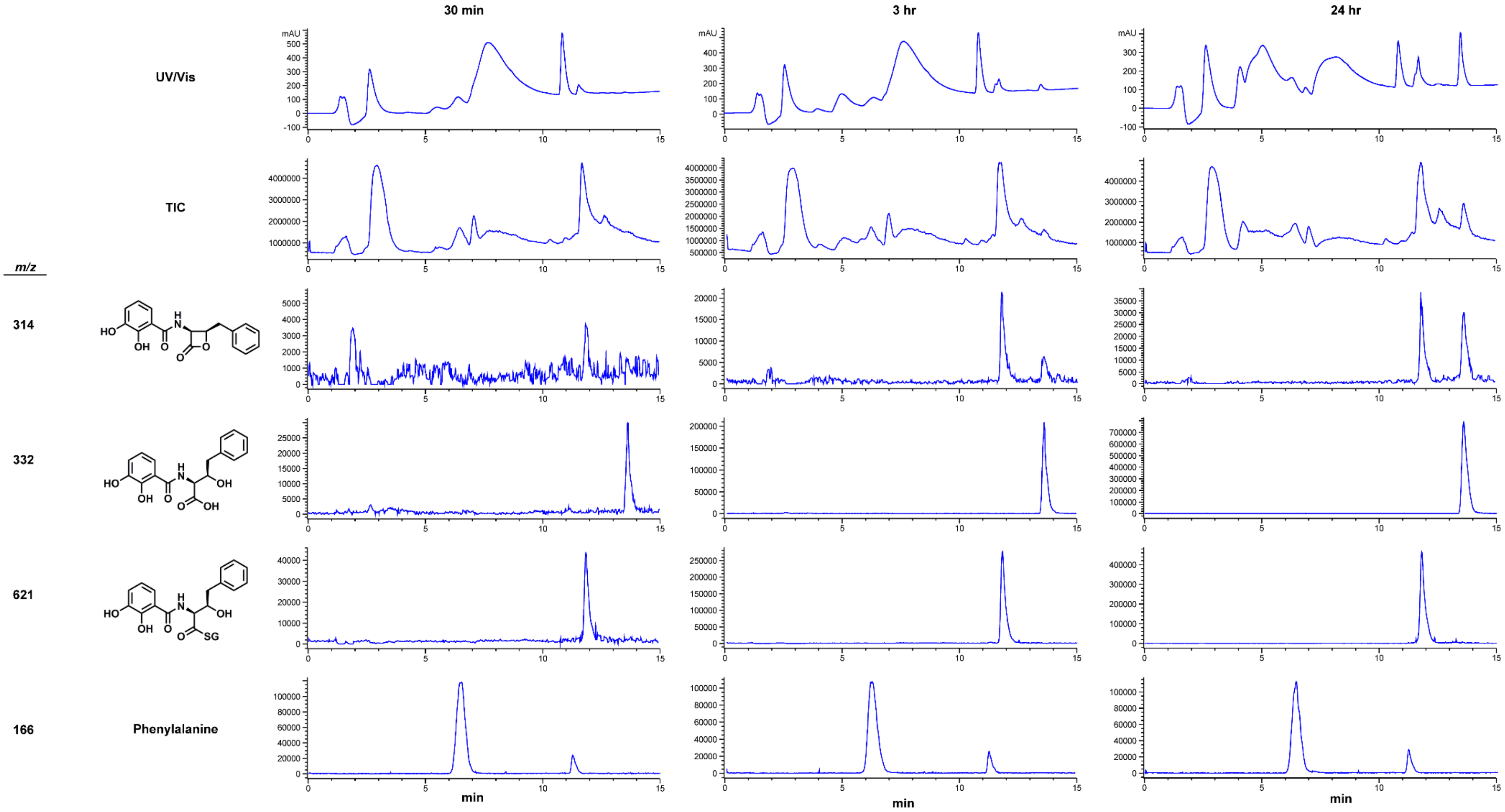

obiF$_1$_del_2_MLP (10 μM)

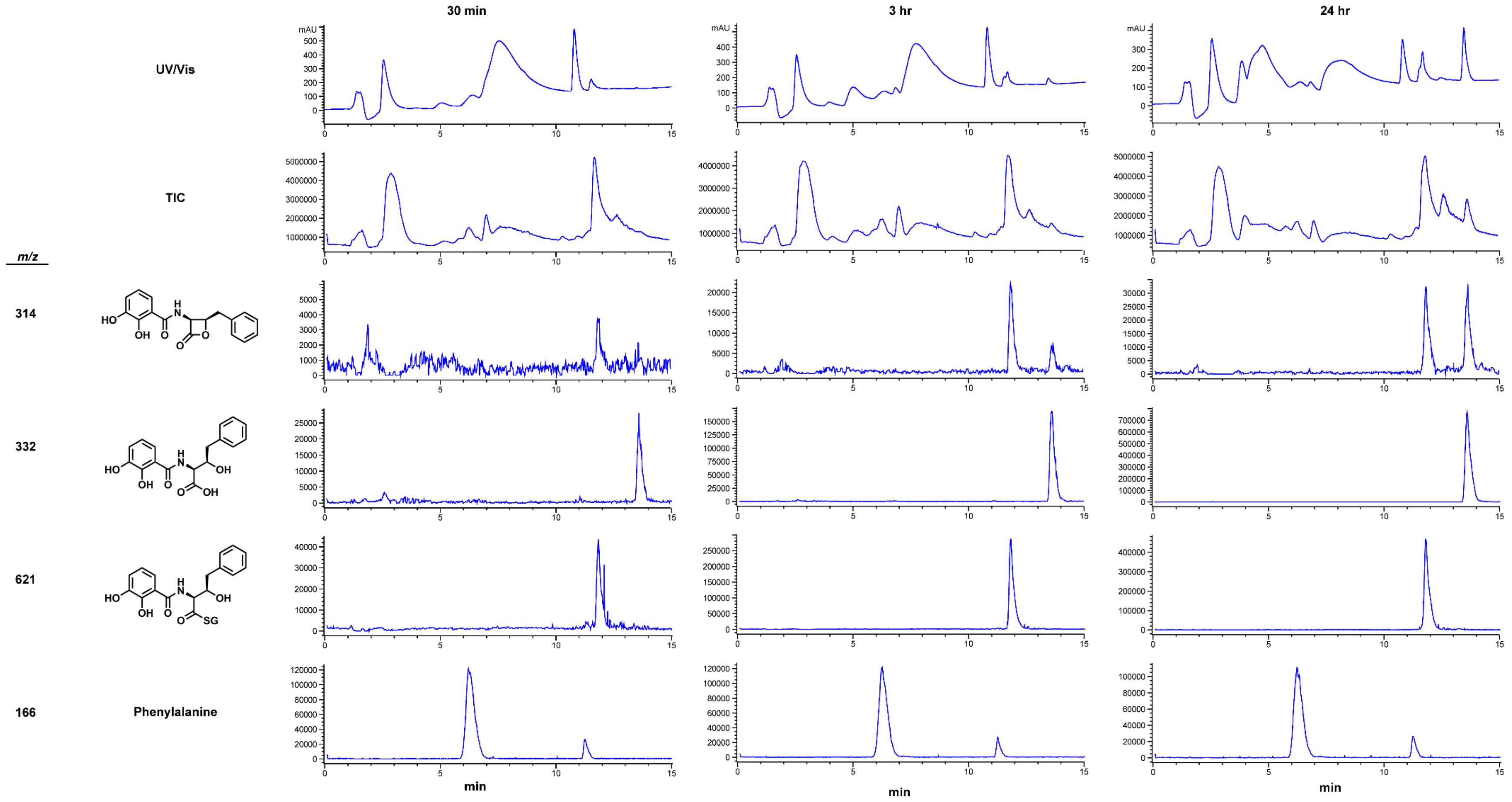

obiF$_1$_del_1_MLP (25 µM)

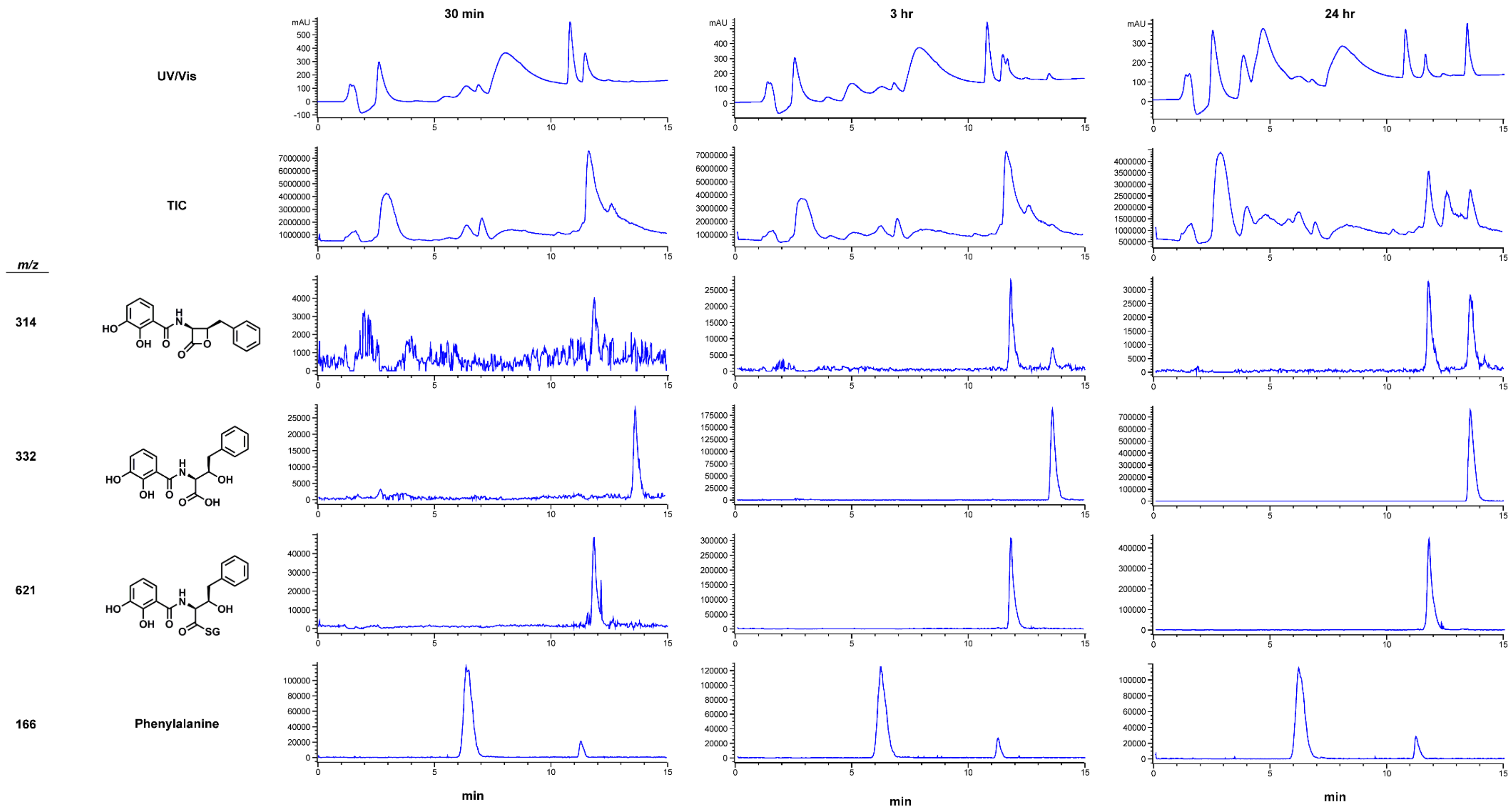

obiF$_1$_del_2_MLP (25 μM)

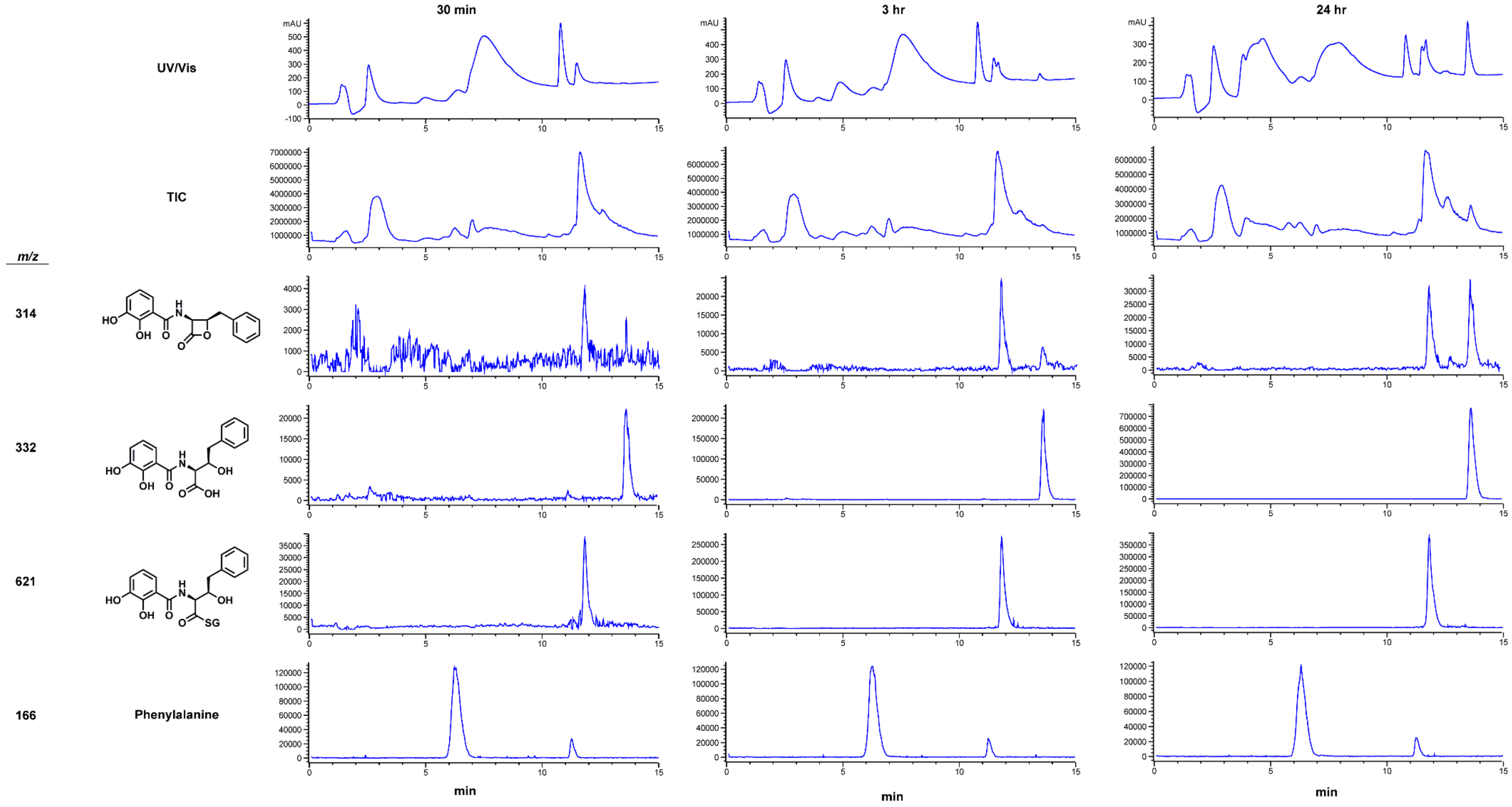

obiF$_1$_rbs_1_MLP (1 µM)

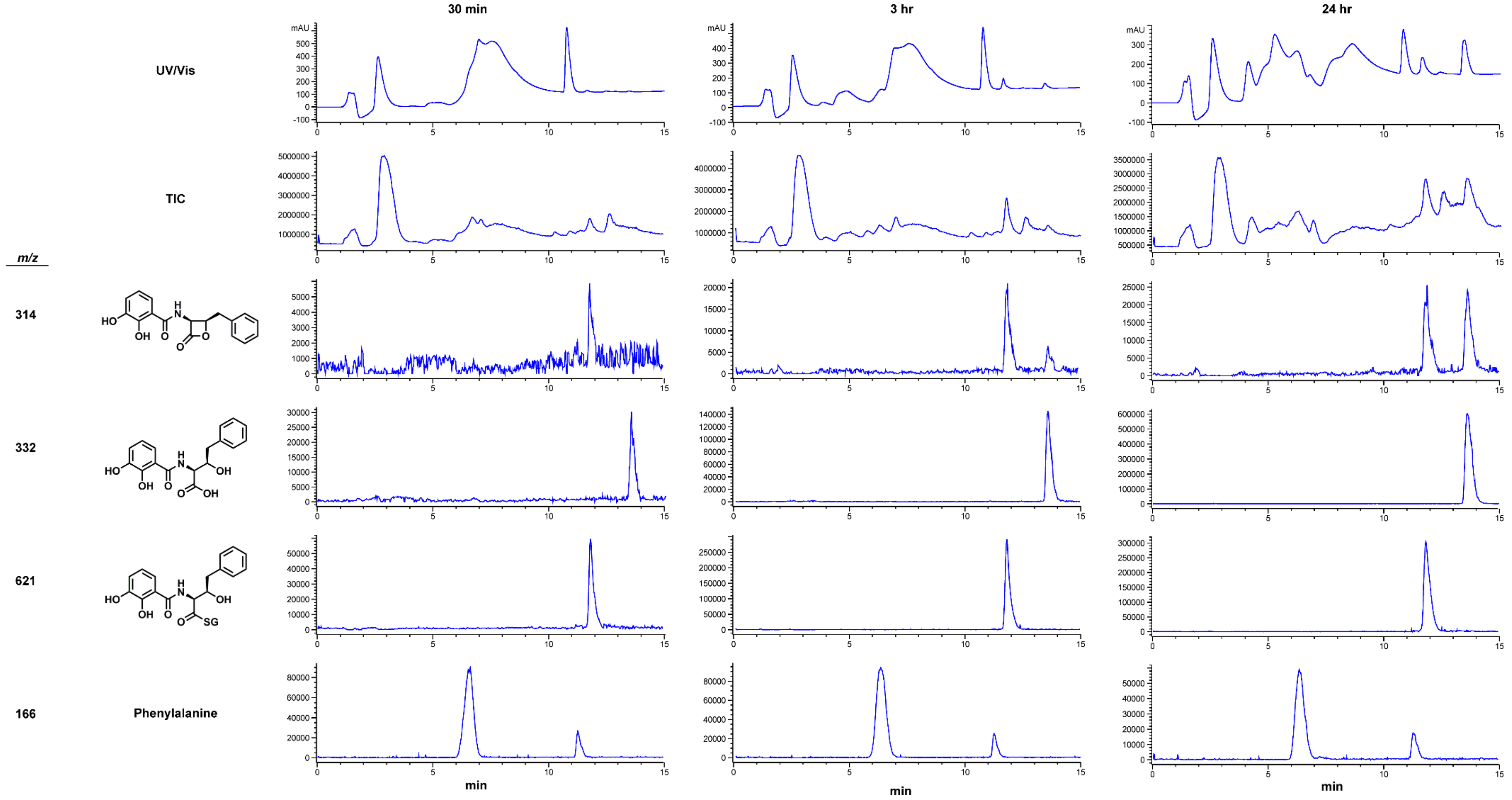

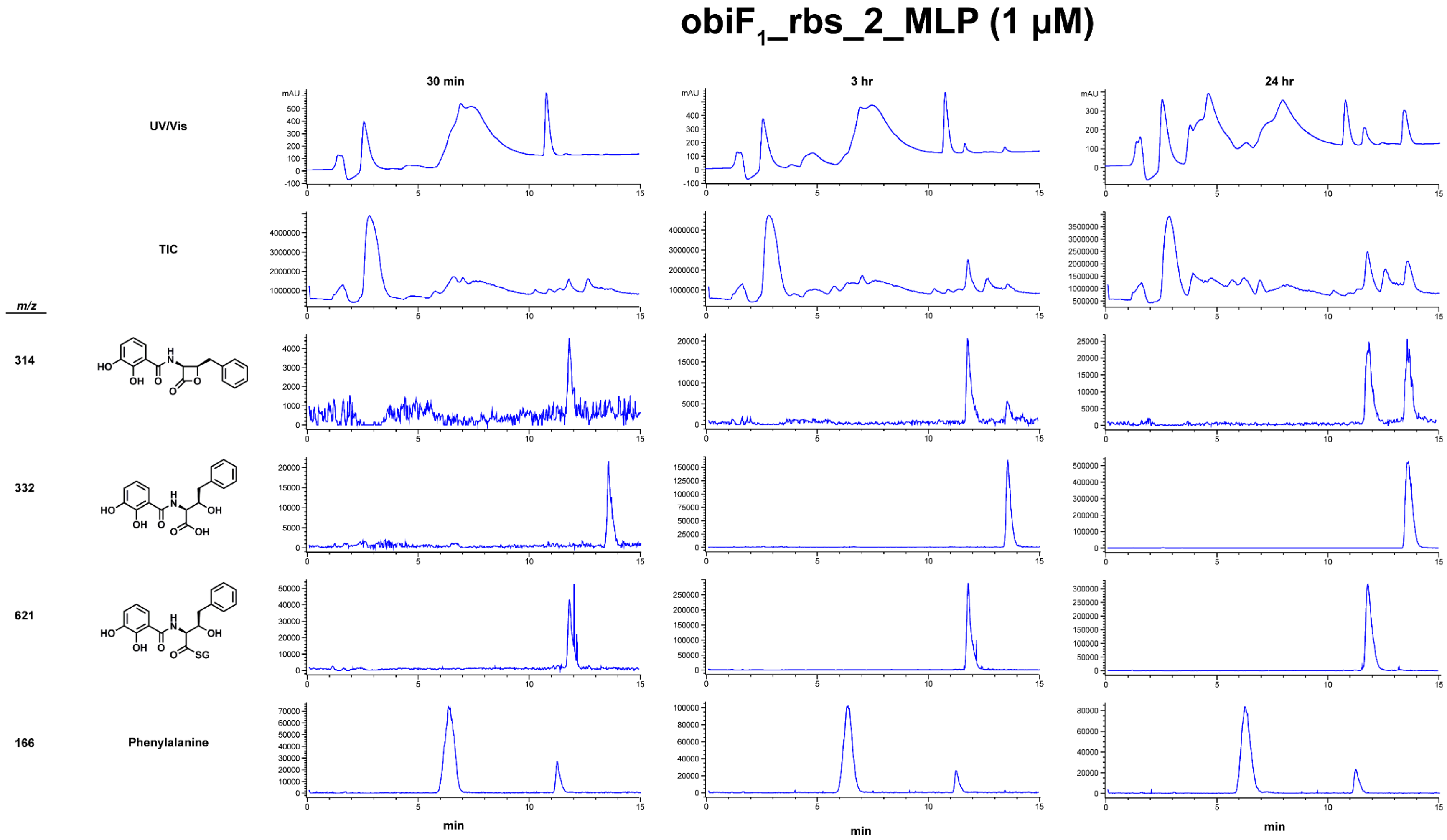
obiF$_1$_rbs_2_MLP (1 μM)
30 min
3 hr
24 hr
UV/Vis
TIC
m/z
314
332
621
166
Phenylalanine
mAU
min

obiF$_1$_rbs_1_MLP (10 μM)

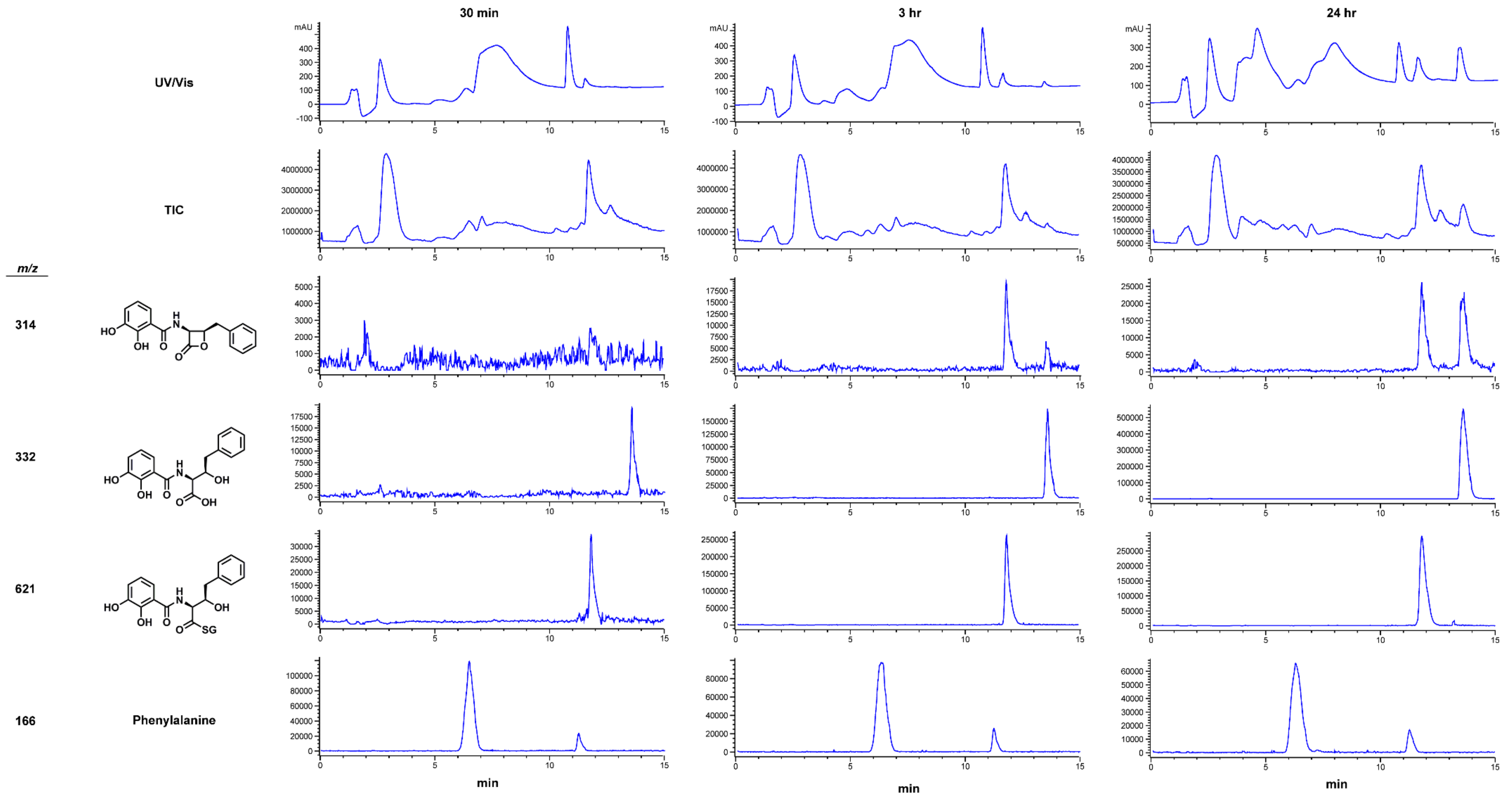

obiF$_1$_rbs_2_MLP (10 μM)

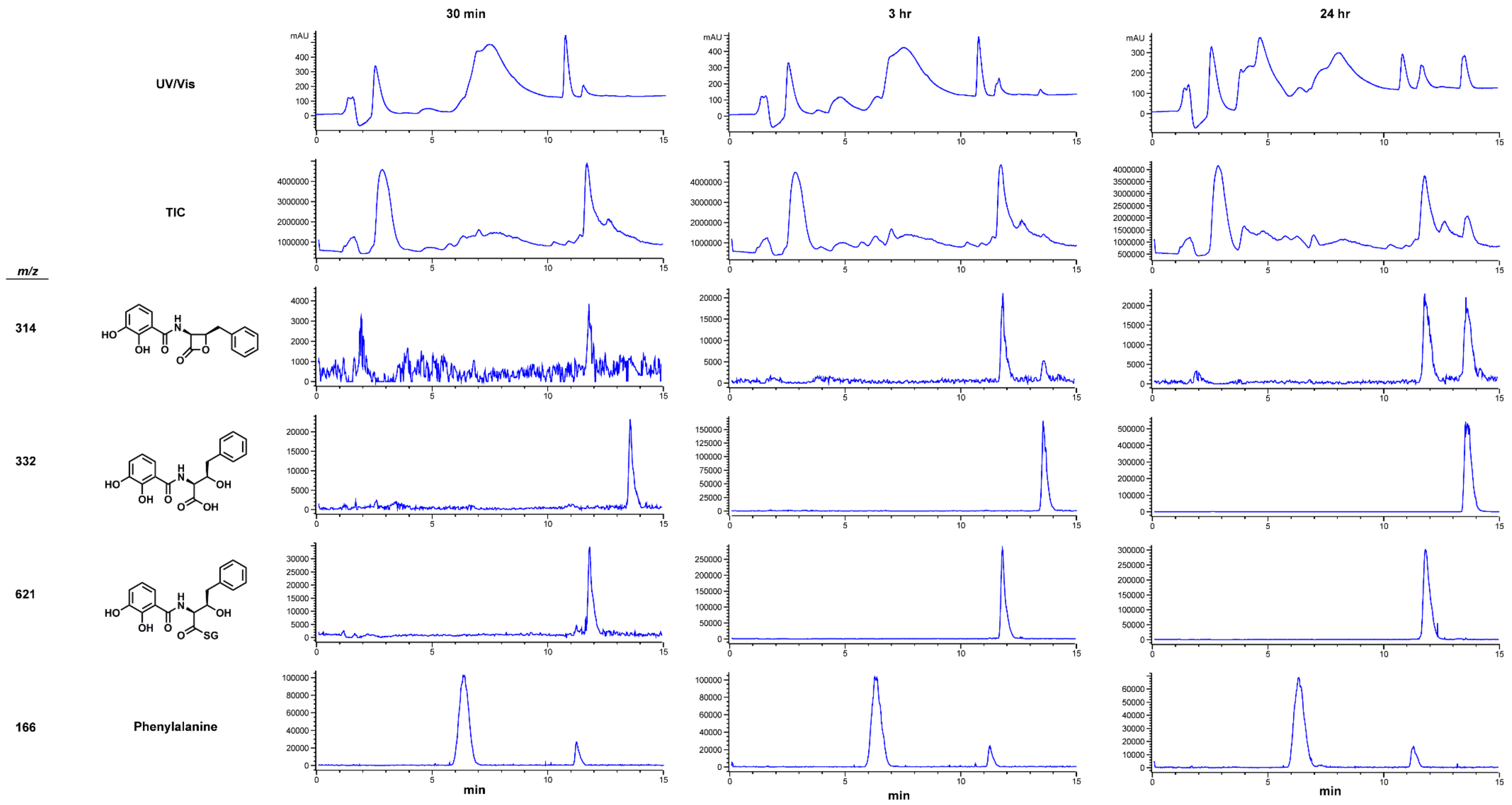

obiF$_1$_rbs_1_MLP (25 μM)

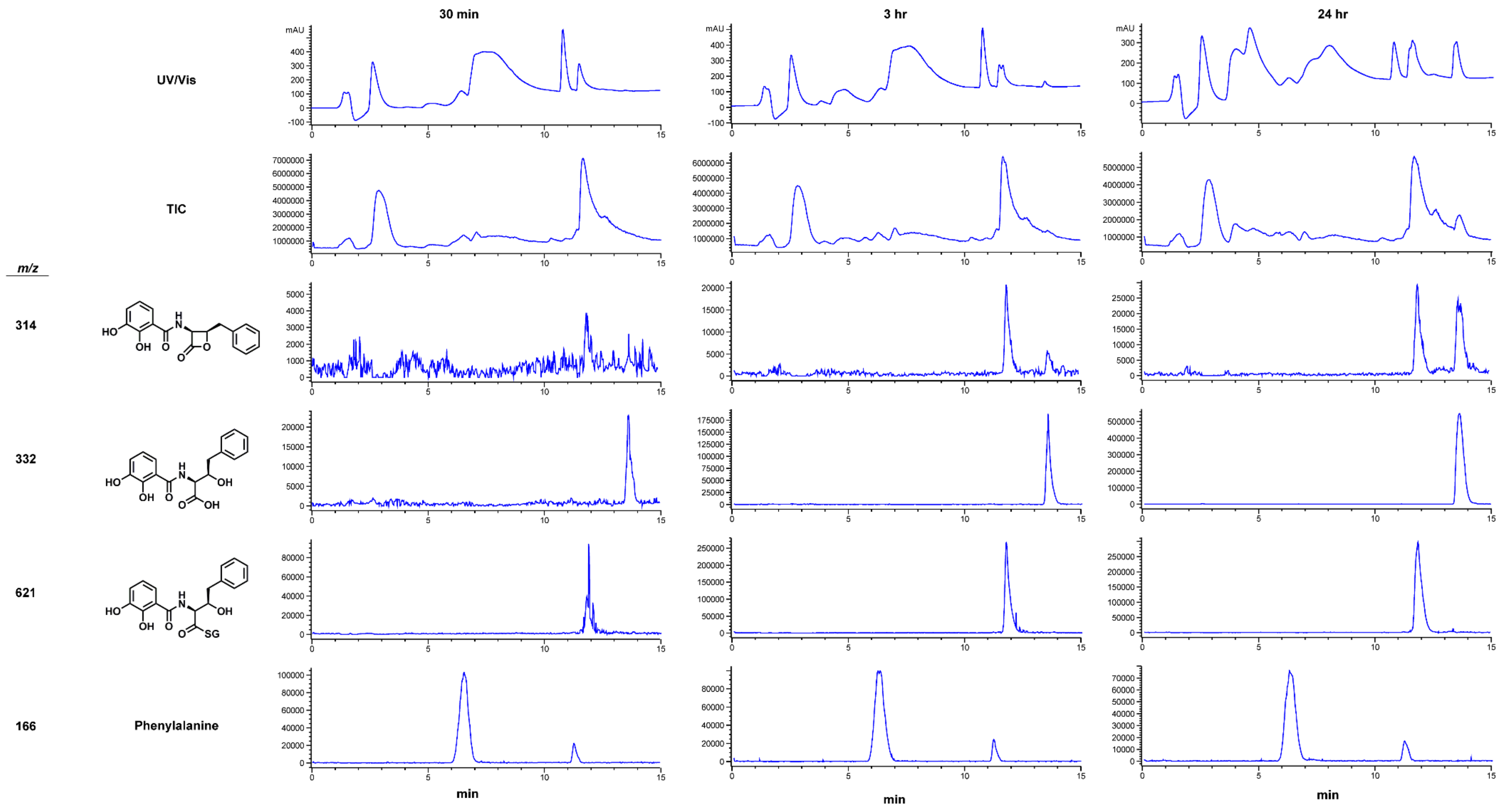

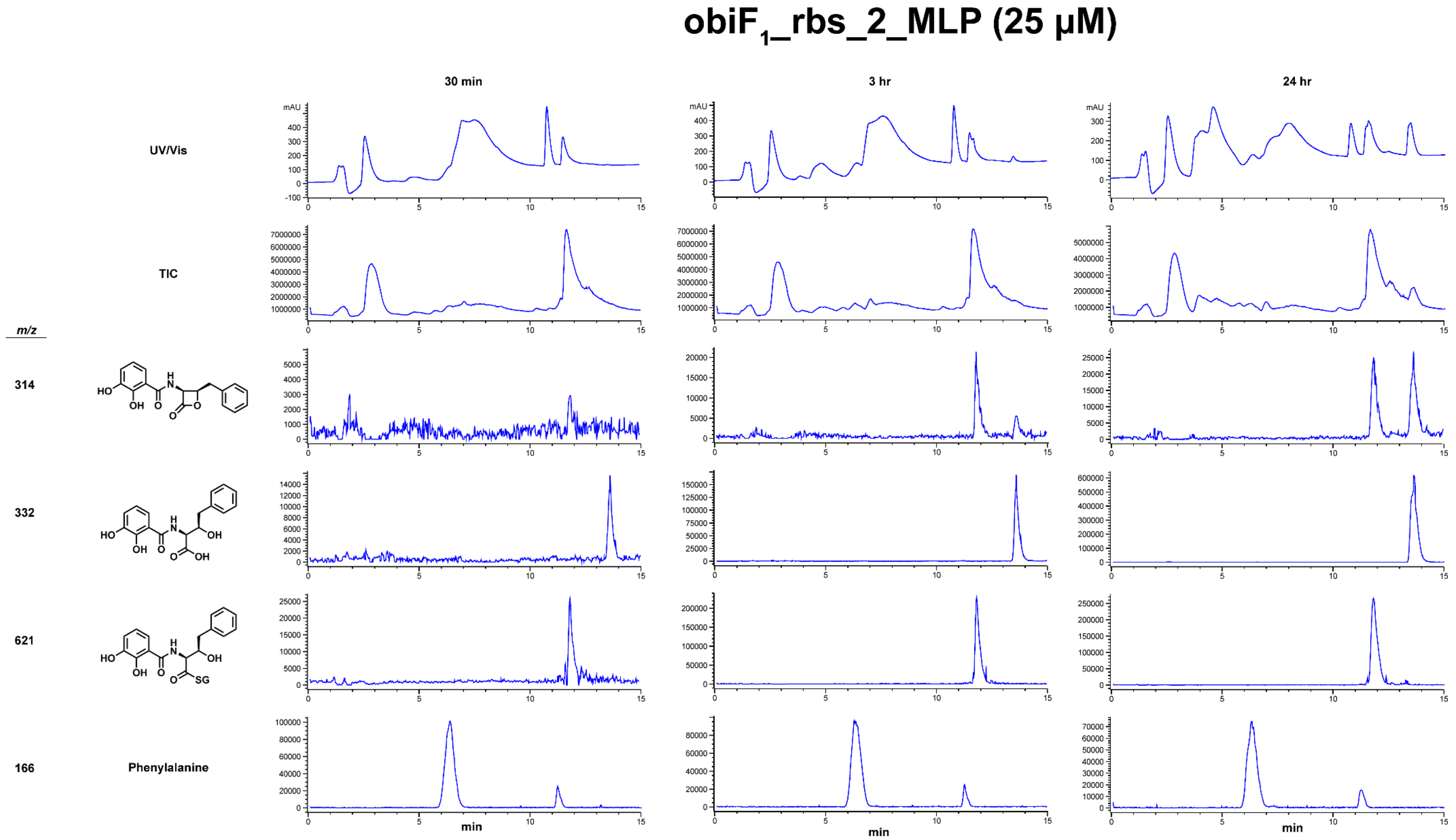

obiF1_rbs_2_MLP (25 μM)
30 min
3 hr
24 hr
UV/Vis
TIC
m/z
314
332
621
166
Phenylalanine
mAU
min

obiF$_1$_A841E_1_MLP (1 μM)

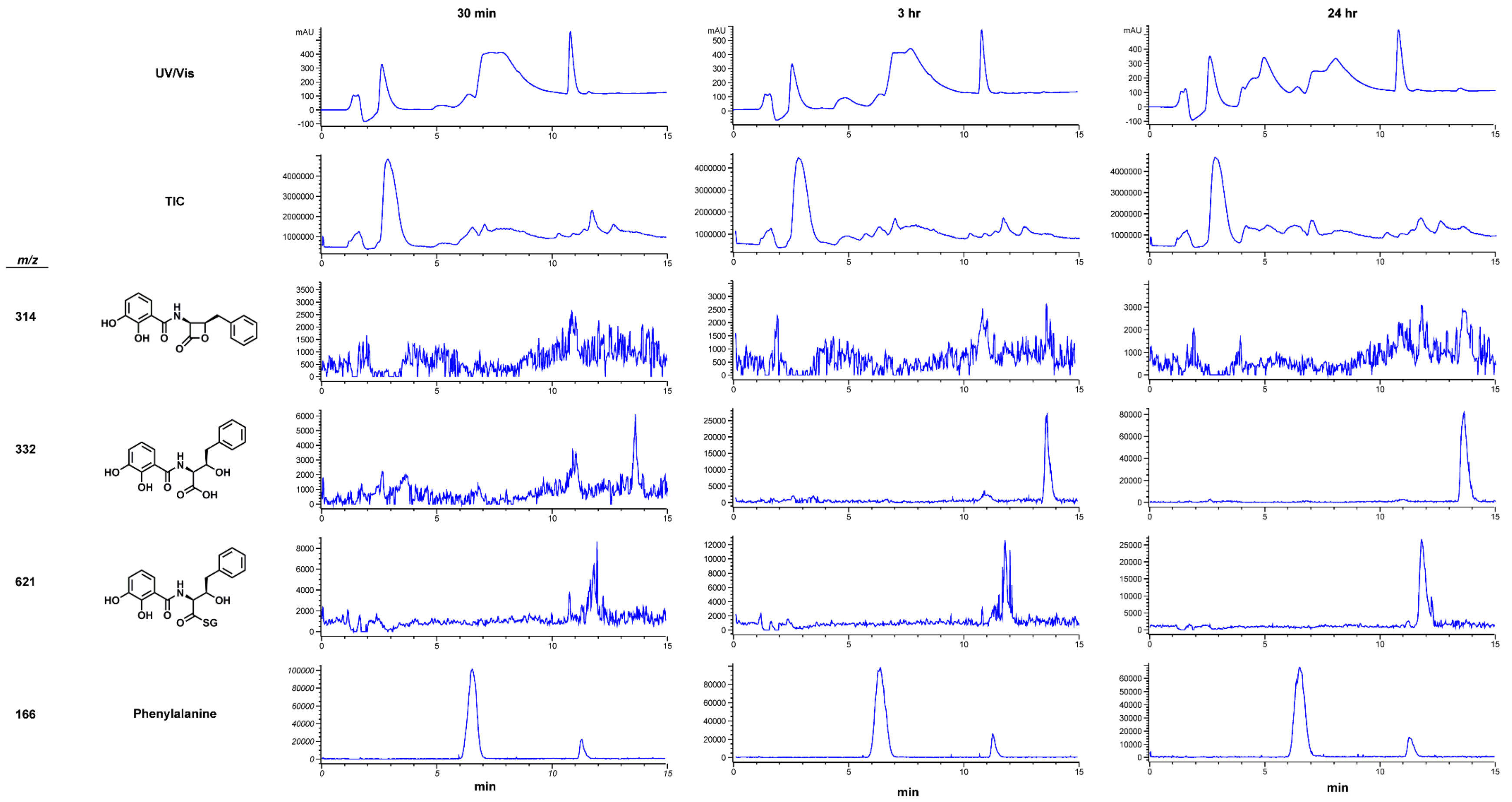

obiF$_1$_A841E_2_MLP (1 μM)

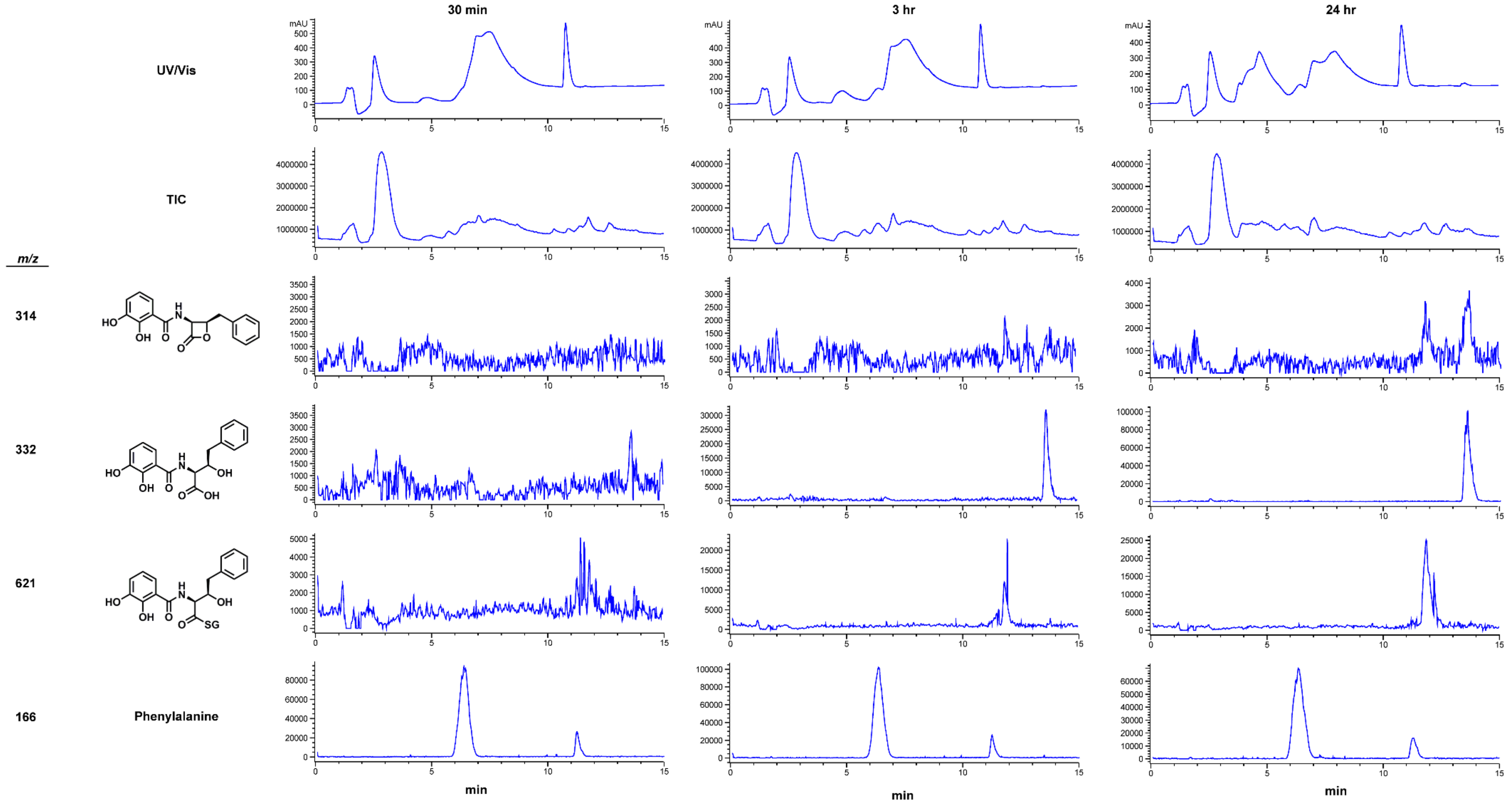

obiF$_1$_A841E_1_MLP (10 μM)

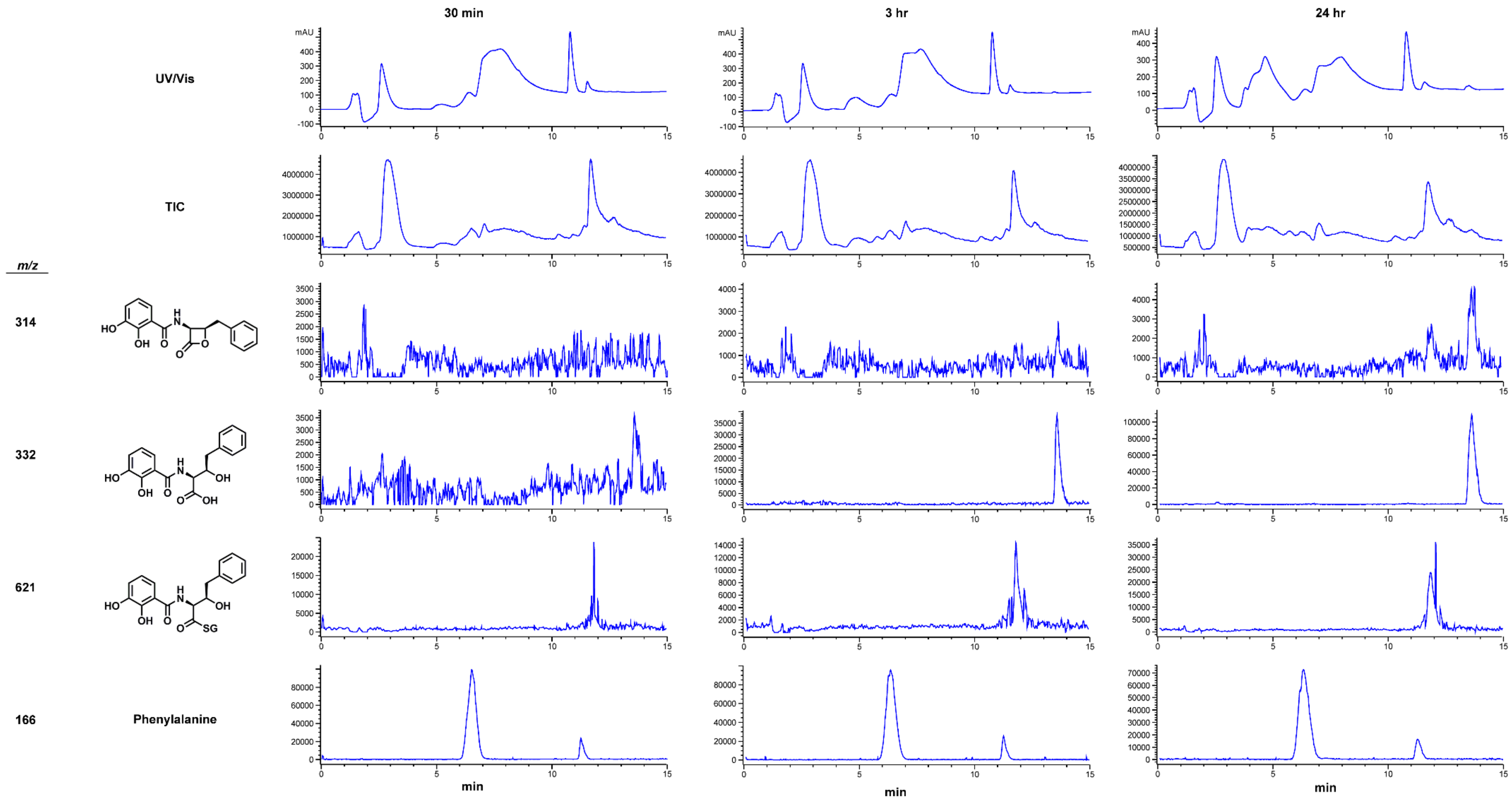

obiF$_1$_A841E_2_MLP (10 μM)

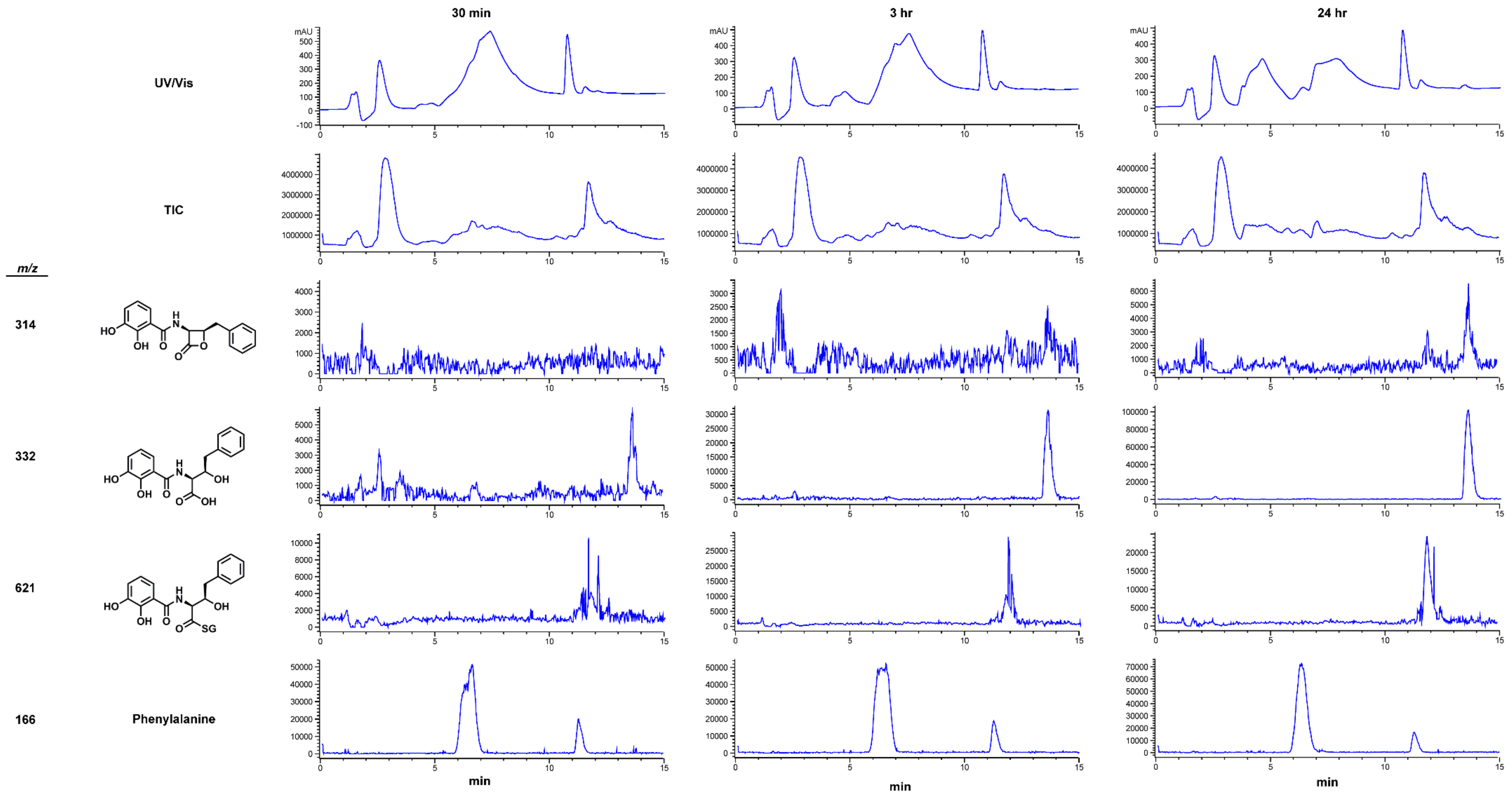

obiF$_1$_A841E_1_MLP (25 μM)

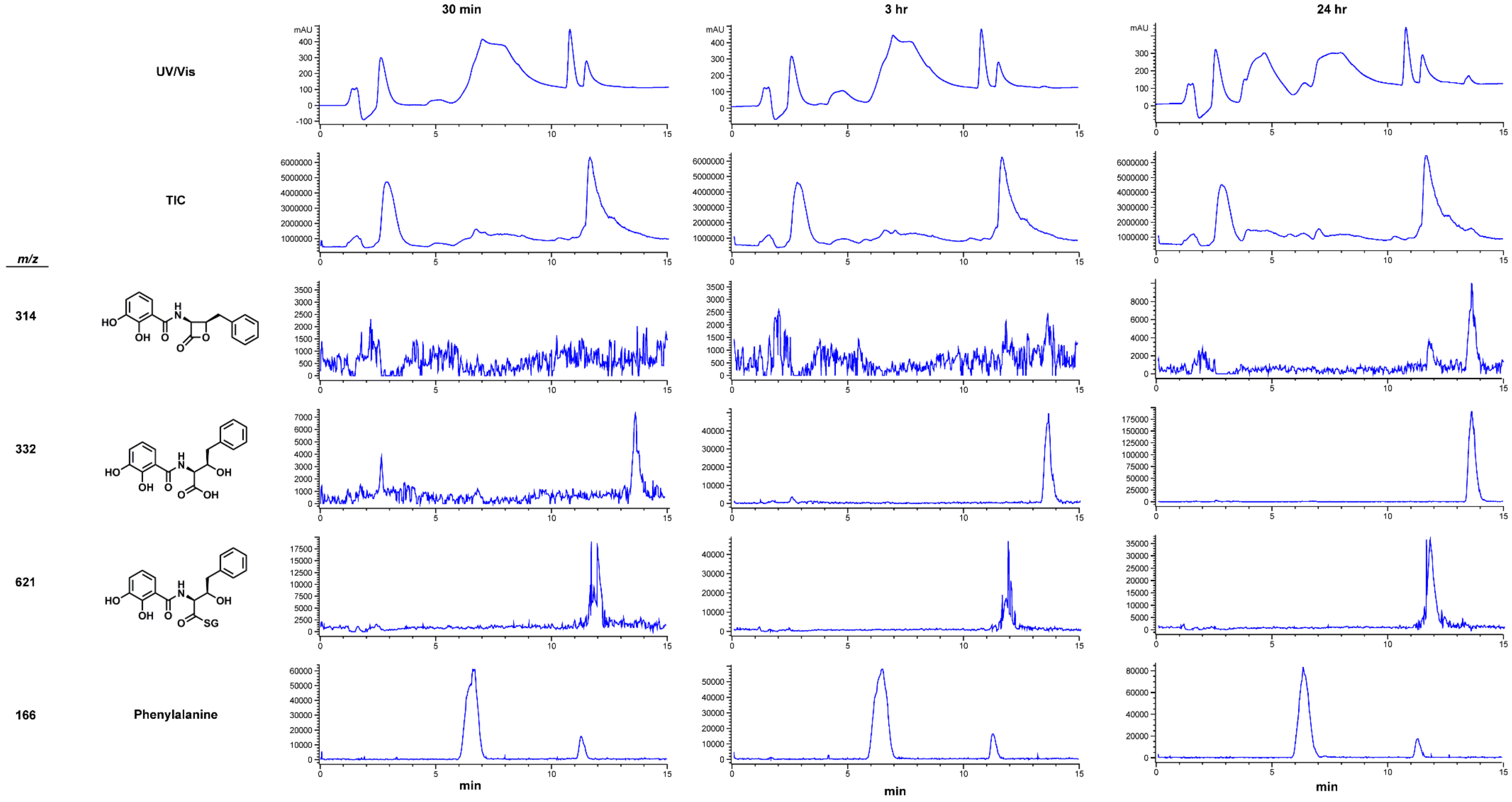

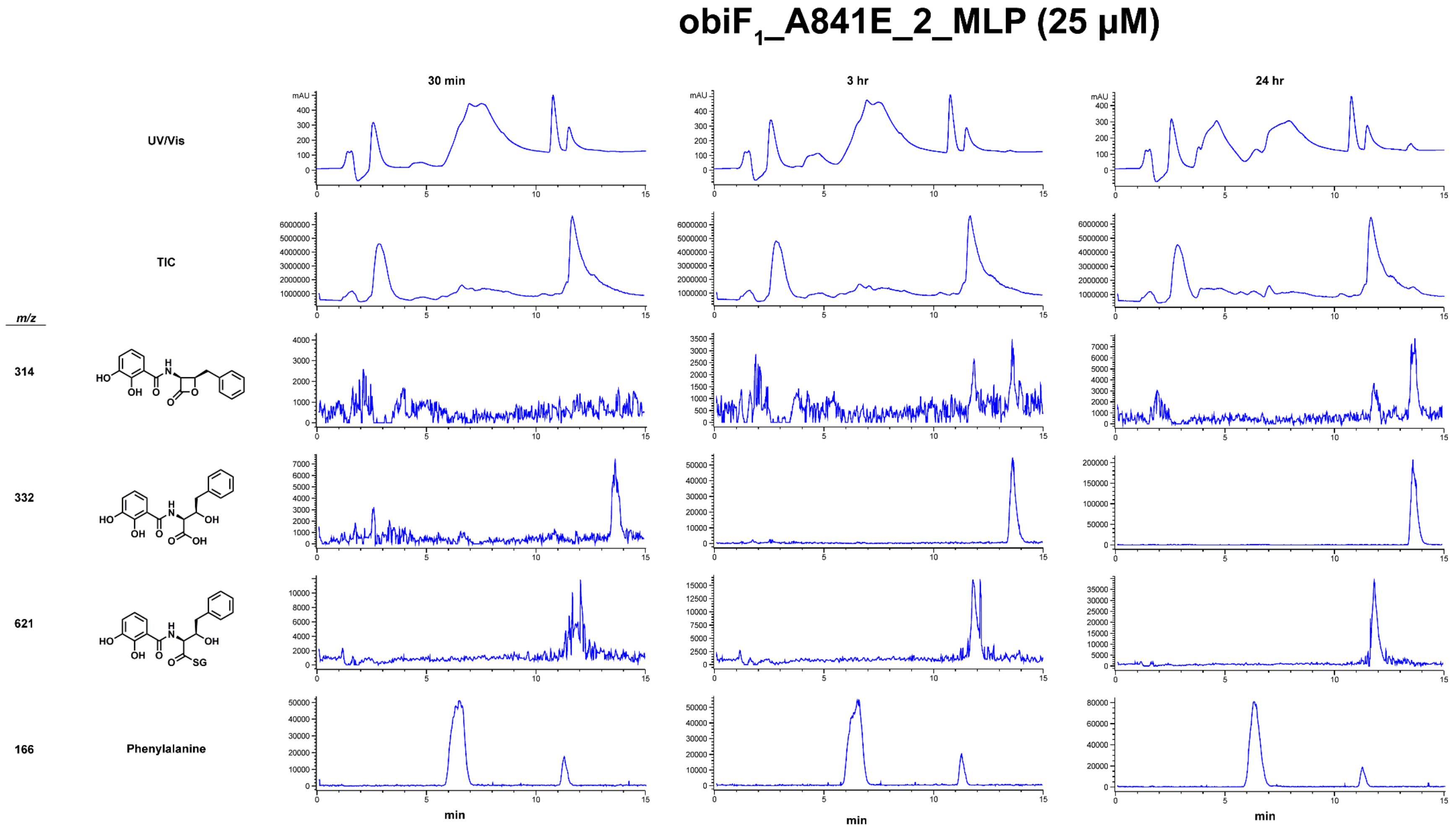

obiF1_A841E_2_MLP (25 μM)
30 min
3 hr
24 hr
UV/Vis
TIC
m/z
314
332
621
166
Phenylalanine
min
